# Liuwei Dihuang Pills Inhibit Podocyte Injury and Alleviate IgA Nephropathy by Directly Altering Mesangial Cell-Derived Exosome Function and Secretion

**DOI:** 10.3389/fphar.2022.889008

**Published:** 2022-07-11

**Authors:** Xiaodong Zhu, Xiaogang Shen, Bo Lin, Jiaxi Fang, Juan Jin, Qiang He

**Affiliations:** ^1^ Bengbu Medical College, Bengbu, China; ^2^ Urology and Nephrology Center, Department of Nephrology, Zhejiang Provincial People’s Hospital (Affiliated People’s Hospital, Hangzhou Medical College), Hangzhou, China; ^3^ Zhejiang Chinese Medical University, Hangzhou, China

**Keywords:** IgA nephropathy, Liuwei Dihuang pills, podocytes, PI3K/Akt/mTOR signalling pathway, rapamycin, exosome

## Abstract

**Background:** Immunoglobulin A nephropathy (IgAN) is the most common glomerular disease worldwide. Its pathological features include IgA immune complex deposition, accompanied by mesangial cell proliferation and mesangial matrix expansion. This study was conducted to investigate the effects of Liuwei Dihuang pills (LWDHW) on IgAN in mice and human podocytes, as well as to determine their underlying mechanisms of action.

**Methods:** For *in vitro* experiments, podocytes were exposed to the human mesangial cell culture medium supernatant of glomerular cells treated with aggregated IgA1 (aIgA1) and LWDHW-containing serum. Cell viability and the proportion of positive cells were evaluated using CCK-8 and flow apoptosis kits, respectively. The cells were collected for western blot analysis. Twenty-four mice with IgAN induced by oral bovine serum albumin administration combined with tail vein injection of staphylococcal enterotoxin B were randomly divided into four groups of six mice each: untreated model group, model + LWDHW group, model + rapamycin group, and model + LWDHW + rapamycin group. The normal control group contained six mice. The red blood cell count in the urine, urine protein, blood urea nitrogen, serum creatinine, and IgA deposition were determined, and TUNEL and western blotting were performed in the mouse kidney tissues.

**Results:** *In vitro* experiments showed that LWDHW promoted autophagy by regulating the PI3K/Akt/mTOR signalling pathway and improved the damage to podocytes caused by the aIgA1-treated mesangial cell supernatant. This study demonstrates the effectiveness of LWDHW for treating IgAN. In the animal experiments, LWDHW significantly reduced the urine red blood cell count, serum creatinine and urea nitrogen contents, and 24 h urinary protein function and improved IgA deposition in the kidney tissues, glomerular volume, glomerular cell proliferation and polysaccharide deposition, and glomerular cell apoptosis. The pills also reversed the changes in the LC3II/I ratio and p62 content in the kidney tissues. The combination of LWDHW and rapamycin showed stronger inhibitory effects compared to those of LWDHW or rapamycin alone.

**Conclusion:** LWDHW may improve regulation of the PI3K-Akt-mTOR pathway and inhibit autophagy in podocytes, as well as alleviate IgA nephropathy by directly altering mesangial cell exosomes.

## Introduction

Immunoglobulin A (IgA) nephropathy (IgAN) is the most common form of primary glomerulonephritis worldwide (Wyatt and Julian, 2013) and accounts for approximately 10%–20% of all primary glomerulonephritis cases in the United States. In some European countries, its prevalence reaches approximately 20%–30%. The prevalence of this disease is highest in some developed Asian countries, at approximately 40%–50% (Woo et al., 2010; Lai et al., 2016). The clinical features of IgAN often include asymptomatic proteinuria and haematuria, with proteinuria among the most important prognostic indicators and results from derangement of the podocyte-basement membrane (glomerular basement membrane) glomerular compartment (Novak et al., 2015; Leung et al., 2018; Trimarchi and Coppo 2019). Because of its slow clinical procession, approximately 30%–40% of patients with IgAN develop end-stage kidney disease within 20–30 years of diagnosis (Lai et al., 2016). The main pathological feature of IgAN is an IgA1 glycosylation abnormality resulting from binding of IgA1 to O-sugar chain-deficient galactose (galactose-deficient IgA1). This abnormally glycosylated IgA1 is recognized by IgG and/or IgA1 homotype anti-sugar chain autoantibodies, resulting in the formation of circulating immune complexes that are deposited on the glomerular mesangium (Tomana et al., 1997; Tomana et al., 1999; Lai et al., 2016). The podocytopathic changes result from initial alterations in the mesangial area (Trimarchi and Coppo 2019). The relationship between podocytes and mesangial cells has been described in studies of IgAN wherein the medium transferred from mesangial cells treated with gd-IgA1 induced epithelial-to-mesenchymal transition in podocytes; the PI3-K/Akt pathway was involved in this process. Additionally, medium transfer from human mesangial cells (HMCs) stimulated with gd-IgA1 increased the expression of TNF-α and CTGF, as well as the expression of TNF-α receptors on podocytes to increase podocyte apoptosis (Ebefors et al., 2021).

Autophagy is a highly conserved intracellular catabolic process (Bhatia and Choi 2020) that is precisely regulated to maintain intracellular stability (Liu et al., 2016). In recent years, an increasing number of studies has shown that autophagy plays an important role in maintaining the renal dynamic balance in both healthy and disease conditions. The most typical regulatory signalling pathway for autophagy is the PI3K/Akt/mTOR signalling pathway. Autophagy inhibition in the presence of mTOR stimulation may contribute to podocyte injury in progressive glomerular disease (Weide and Huber 2011; Liu et al., 2017; Chen et al., 2019). Rapamycin, an mTOR inhibitor, alleviated impaired autophagy in podocytes under stimulated conditions of IgAN, thereby reducing podocyte apoptosis and reducing proteinuria and pathological damage in mice with IgAN (Liang et al., 2017; Liu et al., 2017).

As a classical Chinese prescription medicine, Liuwei Dihuang pills (LWDHW) are widely used in China and Europe. LWDHW is composed of six Chinese crude herbs, *Rehmannia glutinosa* Libosch. (Scrophulariaceae family), *Cornus officinalis* Sieb. (Cornaceae family), *Dioscorea opposite* Thunb. (Dioscoreaceae family), *Alisma orientale* (G. Samuelsson) Juz (Alismataceae family), *Poria cocos* (Schw.) Wolf (Polyporaceae family), and *Paeonia suffruticosa* Andrews (Paeoniaceae family) at an 8:4:4:3:3:3 ratio (Cheng et al., 2019). Approximately 80.3% and 64.2% of Chinese and European practitioners, respectively, have prescribed LWDHW (Williamson et al., 2013). In China, LWDHW is widely used to treat various diseases caused by “kidney-yin deficiency” and was first recorded during the Song dynasty. Although LWDHW has been extensively used for the clinical prevention and treatment of various diseases affecting the immune, endocrine, digestive, respiratory, urinary, and nervous systems, its efficacy must be evaluated in research studies and clinical trials. Its effects against chronic inflammation, oxidative stress, and diabetes-related kidney diseases have been confirmed (He et al., 2007; Perry et al., 2014). In addition, LWDHW elicits beneficial effects against renal conditions such as polyuria, dysuria, and oedema. Long-term LWDHW use was shown to improve serum protein concentrations and oedema in patients with renal failure (Xu et al., 2017; Cheng et al., 2019). However, the efficacy of LWDHW for treating IgAN has not been widely examined. Therefore, we evaluated the molecular mechanisms underlying the effects of LWDHW against IgAN in IgAN mice and podocytes. We predicted that LWDHW directly alters mesangial cell-derived exosome function and secretion, thereby inhibiting aIgA1-treated mesangial cell supernatant (MCS)-induced podocyte injury and alleviating IgAN. This study provides a theoretical and experimental basis for IgAN follow-up treatment using LWDHW.

## Materials and Methods

### Preparation of Drug-Containing Serum

To concentrate the LWDHW, 24 Liuwei Dihuang pills (concentrated pills, Chinese medicine, Z43020145, Jiuzhitang, Changsha, China) were dissolved in distilled water and concentrated to 20 ml. Male Sprague–Dawley rats (weight: 280–300 g) of specific pathogen-free grade were randomly divided into two groups: blank group and LWDHW group (LWDHW). An intragastric dose of 20 ml/kg/day was administered to the rats (10 ml/kg twice daily). The blank group was treated with normal saline, whereas the LWDHW group was treated with concentrated liquid LWDHW (the 10 times clinical equivalent dose) for three consecutive days. Blood was collected at 1–2 h after the last administration from the rat eyeballs under aseptic conditions. Serum (control or LWDHW-containing serum) was isolated and inactivated by heating in water a bath at 56°C for 30 min. This serum was further sterilized by filtration though a 0.22 μm filter and stored at −20°C until use.

### Cell Culture

Human primary podocytes were purchased from Huatuobo (HTX2426, OTWO, Shenzhen, China) and cultured in RPMI1640 (PM150110, Procell, Wuhan, China) supplemented with 20 µ/ml γ-interferon, 10% foetal bovine serum (164210-500, Procell), and 1% penicillin/streptomycin (PB180120, Procell) at 33°C in a 5% CO_2_-containing environment. The cells were further cultured at 37°C for 10 days to induce podocyte maturation.

HMCs derived from human glomerular mesangial cells were purchased from Procell (CL-0619) and cultured in Dulbecco’s Modified Eagle Medium (Procell, PM150210) supplemented with 10% foetal bovine serum (164210-500, Procell, China) and 1% penicillin/streptomycin (PB180120, Procell). The cells were subcultured at 37°C in a 5% CO_2_ atmosphere.

### Collection of MCS

Human IgA1 proteins (ab91020, Abcam, Cambridge, United Kingdom) were heated on a dry plate at 65°C for 150 min to obtain aIgA1 (Xia, Liu et al., 2020). Human glomerular HMCs were treated with a solvent or 25 μg/ml IgA1, and further treated with control or drug-containing serum simultaneously. After 24 h, the HMCs were transferred into fresh medium without the solvent, aIgA1, control serum, or drug-containing serum, and the supernatant (MCS) was collected after 24 h of culture.

### Exosome Extraction

After the HMCs adhesion, they were treated with the solvent or 25 μg/ml aIgA1 for 24 h; the solution was replaced with a fresh blank medium, and the supernatant (MCS) was collected after 24 h. Exosomes were extracted using an exosome extraction kit from Wako (293-77601, Japan).

### CCK-8 Assay

Following human podocyte adhesion, differentiation, and maturation, the supernatants of the different mesangial cell cultures were treated with MCS. After 24 h, cell viability was evaluated in each group using a CCK-8 detection kit (20150520, Qihai Biotec, Shanghai, China) according to the manufacturer’s instructions. The absorbance of each well at 450 nm was determined using a Multiskan MK3 Enzyme Mark device (Spectramax M3, Molecular Devices, Sunnyvale, CA, United States).

### Determination of Apoptosis Rates *via* Flow Cytometry

Podocytes treated with different MCS types or left untreated for 24 h were evaluated using a flow apoptosis kit (KGA101, Jiangsu Kaiji Biotech, Yixing, China). Briefly, when the cell fusion rate reached 80%–90%, the cells were digested for 2–4 min with 2.5 g/L trypsin at a cell suspension rate of approximately 90%, cell digestion was stopped by adding serum, and obtain the cell suspension. The cell suspension was filtered through 100-mesh sieves and then centrifuged at 2,000 rpm for 5 min. The supernatant was discarded, and a cell suspension of 2 × 10^5^ cells/ml was prepared in phosphate-buffered saline (PBS). According to the manufacturer’s instructions, dye was added to the sample tubes; the solutions were mixed and incubated at 4°C overnight in the dark. PBS (1 ml) was added to each tube and centrifuged at 2,000 rpm for 10 min to wash off unlabelled antibodies, and the supernatant was discarded. PBS (0.5 ml) was added to each tube to resuspend the cells, and the proportion of positive cells was determined using a flow cytometer (AccuriC6, BD Biosciences, Franklin Lakes, NJ, United States).

### Western Blotting

An appropriate number of cells was collected and washed with PBS; lysate (P0013B, Biyuntian, Shanghai, China) containing phenylmethylsulfonyl fluoride (329-98-6, Nanjing Wohong, Nanjing, China) was added to the sample, and the cells were cleaved during incubation on ice for 30 min. The cell fragments and lysate were transferred to a 1.5 ml centrifuge tube, and the supernatant was obtained by centrifugation at 12,000 rpm for 15 min at 4°C. The concentration of the protein lysate was determined using a Bio-Rad Protein Assay Kit II (5000002, Hercules, CA, United States). The protein lysate was mixed with 5× sample buffer, separated by sodium dodecyl sulphate-polyacrylamide gel electrophoresis, and then transferred onto polyvinylidene fluoride membranes (no. 162-0177, Bio-Rad). The membranes were blocked with 4% milk containing 0.1% tween and anti-LC3 antibody (1:500, 12135-1-AP; Proteintech, Rosemont, IL, United States), anti-p62 antibody (1:500, 18420-1-AP; Proteintech), anti-AKT antibody (1:500, ab8805; Abcam), anti-p-AKT antibody (1:1,000, ab8933; Abcam), anti-mTOR antibody (1:1,000, AF6308; Affinity Biosciences, Cincinnati, OH, United States), anti-p-mTOR antibody (1:1,000, AF3308; Affinity Biosciences), and anti-GAPDH antibody (1:2,500, ab9485; Abcam). After the membranes were washed three times with PBS containing 0.1% Tween (PBST), they were incubated for 2 h with horseradish peroxidase-labelled secondary antibodies (Dianova, Hamburg, Germany). The membranes were washed three times with PBST. An ECL developer (no. 170-5060, Bio-Rad) was dropped on the membranes, which were placed in a GelDoc imaging system (Bio-Rad) for image collection. Protein expression levels were normalized to those of the internal reference protein, GAPDH.

### Exosome Transmission Electron Microscopy

Exosomes were observed by staining with an Exosome Electron Microscopy Analysis Kit (Weihui Bio, E1610, Beijing, China). First, 2–3 drops of the exosome suspension were placed on a parafilm membrane, and an electron microscopy (EM) net was placed on the surface of exosome suspension. The EM net was allowed to absorb the exosomes for 10 min. Washing buffer was placed on a clean Parafilm membrane; the EM net was transferred to the wash buffer and left standing for 30 s before washing with wash buffer. After repeating the washing steps, the EM solution was dropped on a clean sealing membrane, the EM net was transferred to the EM solution, and the samples were allowed to stand for 10 min and then rinsed twice. The EM mesh was transferred onto a filter paper and dried at room temperature, followed by scanning of the exosomes under a transmission electron microscope (JSM-IT300LV, JEOL, Tokyo, Japan).

### Animal Treatment

Thirty eight-week-old male Balb/c mice (20 ± 5 g) were administered 200 ml/kg of 0.1% bovine serum albumin (BSA)-containing acidified water (0.1% dilute hydrochloric acid) daily for the first 5 weeks. During week 6, the mice were intravenously administered 0.2 ml of 2 mmol/L phosphate buffer containing 0.1% BSA once daily for three consecutive days. At week 8, the mice were administered staphylococcal enterotoxin (SEB, 10503027, Thermo Fisher Scientific, Waltham, MA, United States) *via* tail vein injection once weekly for 3 weeks at increasing doses of 0.5 mg/kg, 0.6 mg/kg, and 0.8 mg/kg (Jia et al., 2007; He et al., 2010; Wang, Fu et al., 2013), during weeks 8, 9, and 10, respectively. After SEB injection at week 8, surviving model mice were divided into four groups of six mice each. Untreated male Balb/c mice (20 ± 5 g) were administered the same quantity of normal saline by injection and used as controls (*n* = 6). The specific groups were the 1) control group, 2) model group, 3) model + LWDHW group, 4) model + rapamycin group, and 5) model + LWDHW + rapamycin group. Mice in groups 1 and 2 were administered equal quantities of distilled water by intragastric administration, mice in groups 3 and 5 were administered a concentrated LWDHW water solution via intragastric administration, and mice in groups 4 and 5 were treated with rapamycin (53123-88-9, MedChemExpress, Monmouth Junction, NJ, United States). The intragastric dose of LWDHW was 10 ml/kg (clinical equivalent dose), administered twice daily. LWDHW was concentrated as follows: 24 Jiuzhitang Liuwei Dihuang pills (concentrated pills, Chinese medicine, Z43020145) were dissolved in distilled water and concentrated to 120 ml. Mice in the rapamycin-treated group were intraperitoneally administered 2 mg/kg rapamycin every 2 days (Xiao et al., 2014). After the mice were treated *via* intragastric administration of the traditional Chinese medicine for 4 weeks, the experiment was terminated, and the mice were analysed.

### Detection of Haematuria *via* Light Microscopy

Urine (10 μl) was collected from mice in each group to determine the urine red blood cell counts using a light microscope.

### Determination of Urinary Protein Content

The 24-h urine of mice was collected using a metabolic cage to evaluate the urinary protein content. The standard and the appropriate volumes of samples were added to a 96-well plate. A working solution of the BCA protein concentration determination kit (P0012S, Beyotime, Shanghai, China) was added to each well, and the plate was incubated at 37°C for 30 min. The absorbance at a wavelength of 562 nm was determined using an enzyme labelling instrument, and the protein concentrations of the samples were calculated based on the standard curve and sample volume.

### Determination of Serum Creatinine and Blood Urea Nitrogen Levels

Blood samples collected from the abdominal aortas of the mice were placed at room temperature (20–22°C) for 2 h, and then centrifuged at 3,500 rpm for 5 min. A serum creatinine detection kit (C011-1, Nanjing Jiancheng Bioengineering Institute, Nanjing, China) and urea nitrogen detection kit (C013-2, Nanjing Jiancheng Bioengineering Institute) were used to detect serum creatinine and urea nitrogen, respectively.

### Detection of IgA Deposition *via* Immunofluorescence

Mouse tissues were embedded in paraffin, sliced, and dewaxed in water, placed into boiled citrate buffer for antigen repair, and naturally cooled to room temperature. Next, primary antibodies were dropped on the tissues, which were incubated overnight at 4°C. The tissues were washed three times with PBS, secondary antibodies were added to them, and the tissues were incubated at 37°C for 1 h. The sections were rinsed four times with PBST for 3 min each time. After adding DAPI to the sections, the tissues were incubated for 5 min in the dark to stain the nuclei. Excess DAPI was removed by washing 4 times with PBST for 5 min each. Liquid was dried from the sections using absorbent paper, the films were sealed with sealing solution containing an anti-fluorescence quenching agent, and images were collected under a fluorescence microscope.

### Periodic Acid–Schiff Staining Assay

After dehydration, transparent with xylene, wax immersion, and embedding were carried out on the tissues. The samples were sliced (RM2016 rotary slicer, Leica, Wetzlar, Germany); the paraffin-embedded slices were dewaxed in water, treated with 1% periodate aqueous solution for 10 min, washed with tap water, washed with distilled water, reacted with Schiff reagent (leagene, DG0005) for 10 min, rinsed with water for 10 min, treated with Harris haematoxylin for 10 min, rinsed with tap water to remove excess dye solution, washed with distilled water, treated with 1% hydrochloric acid ethanol differentiation solution for 10 s, washed with tap water and distilled water, washed using 1% of ammonia for 10 s, washed with tap water and distilled water, dehydrated by step-by-step ethanol dehydration, treated with xylene transparent, sealed with neutral resin, and examined under a microscope.

### Terminal Deoxynucleotidyl Transferase dUTP Nick End Labelling Staining Assay

After the slices were dewaxed in water, a terminal deoxynucleotidyl transferase dUTP nick end labelling apoptosis detection kit (BA27, Biobox, Nanjing, China) was used to evaluate apoptosis. DNase-free proteinase K working solution was dropped on the slices at 37°C for 30 min for digestion, and the slices were rinsed three times with PBS for 5 min each time. The slices were placed in 3% H_2_O_2_ at 37°C in the dark for 10 min, and then rinsed three times with PBS for 3 min each time. DNase I reaction solution containing active 100 μl was dropped on the samples, which were then incubated at 37°C for 30 min. The slices were washed three times with PBS for 5 min at each time. The areas around the samples were dried using absorbent paper, and TdT enzyme reaction solution was added to the samples for incubation in a wet box at 37°C for 60 min in the dark. The samples were washed three times with PBS and dried with absorbent paper. Streptavidin-FITC-labelled working solution was added to the samples and incubated at 37°C for 30 min in the dark. The samples were rinsed three times with PBS in the dark and dried. Peroxidase-conjugated anti-horseradish peroxidase labelled working solution was added for incubation at 37°C for 30 min in the dark, followed by washing three times in the dark with PBS. The samples were dried using absorbent paper, and then DAB working solution (FL-6001, Shanghai Long Island Biotech Co., Shanghai, China) was added. The colour reaction was developed at 37°C for 30 s. The samples were further re-stained with haematoxylin for 3 min, treated with 1% hydrochloric acid alcohol differentiation solution, dehydrated through step-by-step ethanol dehydration, treated with xylene to make the samples transparent, sealed with neutral resin, and examined under a microscopic.

### Statistical Analysis

GraphPad Prism 10.0 statistical software (GraphPad, Inc., La Jolla, CA, United States) was used for data analysis. All data are expressed as the mean ± standard deviation. Pairwise comparison between the different groups was performed using the least significant difference method (minimum significance method) using one-way analysis of variance. Values of *p* < 0.05 indicated statistically significant differences.

## Results

### LWDHW-Containing Serum Targeted Mesangial Cells Can Reduce aIgA1-Treated MCS-Induced Podocyte Injury

**FIGURE 1:**
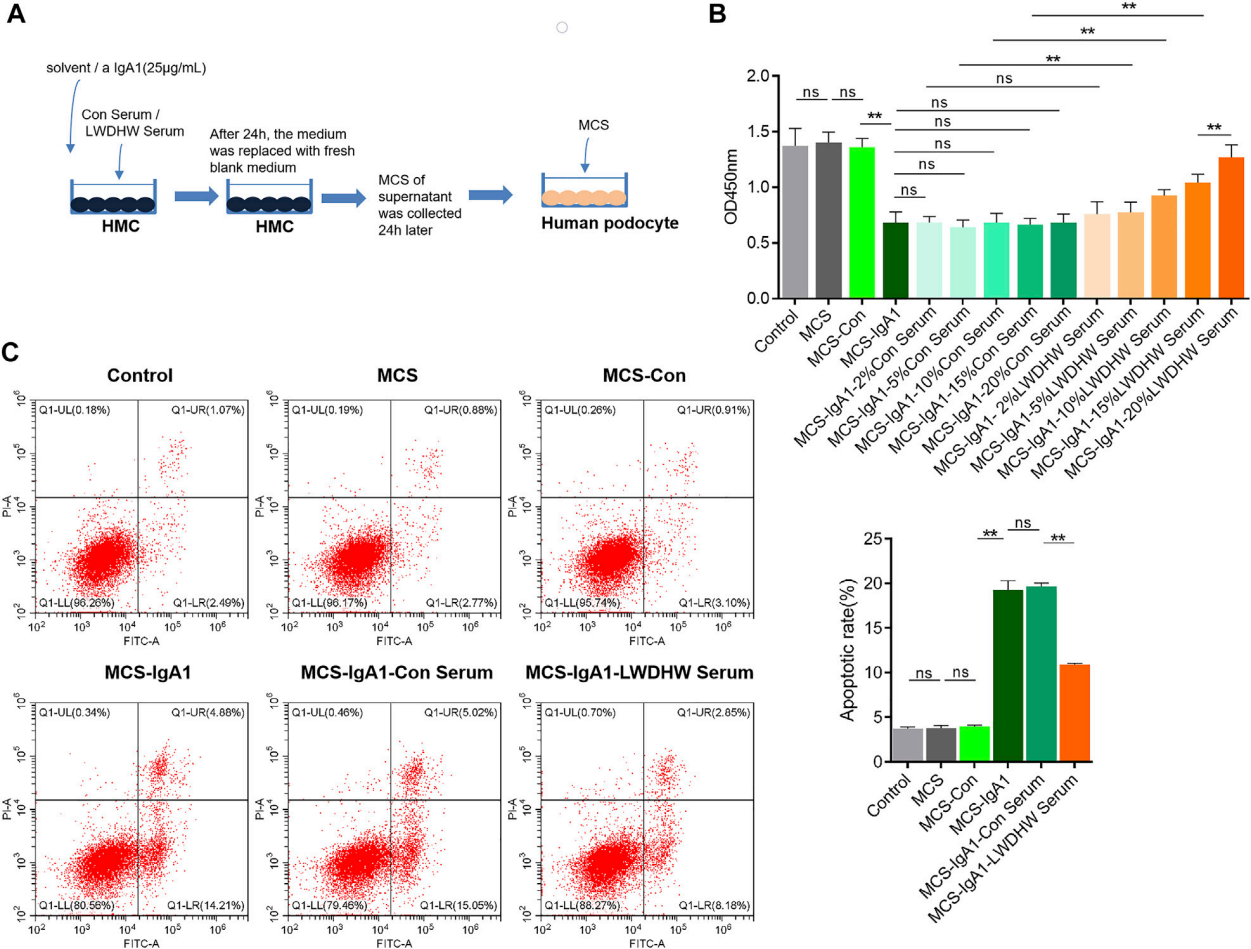
Detection of podocyte damage caused by supernatant of Mesangial cells treated with drug-containing serum and aIgA1. **(A)** The flow chart of cell treatment. **(B)** The change of podocyte activity treated with Mesangial cell supernatant for 24 h by CCK8. **(C)** The apoptosis level of podocyte treated with Mesangial cell supernatant for 24 h was detected by flow cytometry. LWDHW means Liuwei Dihuang pills. Control group: human podocytes without any treatment, MCS group: Represents human podocytes treated with supernatant secreted by mesangial cells without any treatment, MCS-Con group: human podocytes treated with supernatant secreted by Mesangial cells treated with solvent, MCS-IgA1: Human podocytes treated with supernatant secreted by Mesangial cells treated with 25 μg/ml aIgA1 for 24 h, MCS-IgA1-ConSerum: the supernatant secreted by Mesangial cells treated with 25 μg/ml aIgA1 and 20% control serum for 24 h was used to treat human podocytes, MCS-IgA1-LWDHWSrum: the supernatant secreted by Mesangial cells treated with 25 μg/ml aIgA1 and 20% Liuwei Dihuang pills containing serum for 24 h was used to treat human podocytes. ** means *p* < 0.01, ns means *p* > 0.05.

To determine whether LWDHW affects mesangial cells and inhibits the effects of the aIgA1-treated MCS on podocytes, we obtained the MCS (Figure 1A). Several MCS were used to treat human podocytes, and podocyte activity in each group was evaluated in a CCK-8 assay. Compared to the activity of untreated podocytes, there was no significant change in the activity of podocytes treated with the untreated MCS (*p* > 0.05). The supernatant of mesangial cells treated with the solvent did not affect podocyte activity (*p* > 0.05). Compared to the solvent-treated MCS, the supernatant of mesangial cells treated with aIgA1 significantly decreased podocyte activity (*p* < 0.05, Figure 1B). Furthermore, the MCS alone did not affect podocyte activity; however, the aIgA1-treated MCS reduced podocyte activity, indicating that the aIgA1-treated MCS-induced podocyte injury model was successfully established.

Compared to the group treated with the MCS without the control serum, those treated with the MCS treated with different concentrations of the control serum showed no significant change in podocyte activity (*p* < 0.05, Figure 1B). This finding indicates that mesangial cells treated with the control serum did not reverse the podocyte injury induced by the supernatant of aIgA1-treated mesangial cells. In addition, compared to the supernatant of mesangial cells treated with the control serum and aIgA1, that of mesangial cells treated with LWDHW-containing serum (5%–20%) and aIgA1 decreased the inhibitory effects on podocyte activity (*p* < 0.05, Figure 1B). The supernatant of mesangial cells treated with 20% LWDHW-containing serum and aIgA1 exhibited the lowest inhibitory effects against podocyte activity (*p* < 0.05, Figure 1B). Therefore, a follow-up experiment was carried out using 20% LWDHW drug-containing serum to determine the mesangial cell-mediated effects of LWDHW in reducing aIgA1-treated MCS-induced podocyte injury.

As shown in Figure 1C, the supernatant of untreated mesangial cells and supernatant of mesangial cells treated with the solvent did not affect podocyte apoptosis levels compared to those in the control group (*p* > 0.05). Compared to the MCS-Con group, podocyte apoptosis levels were significantly increased in the MCS-IgA1 group. We predicted that the MCS alone does not affect podocyte apoptosis levels, whereas the aIgA1-treated MCS increases podocyte apoptosis levels. In addition, the supernatant secreted by mesangial cells following treatment with the solvent did not significantly affect podocyte apoptosis. Compared to the MCS-IgA1 group, there was no significant change in podocyte apoptosis in the groups treated with 20% control serum- and aIgA1-treated MCS. Additionally, mesangial cells treated with the control serum did not reverse the podocyte apoptosis-inducing effects of the supernatant secreted by aIgA1-treated mesangial cells. Compared to the MCS-IgA1-20% Con serum group, podocyte apoptosis levels in the groups treated with the MCS significantly decreased following treatment with 20% LWDHW-containing serum and aIgA1. This indicates that treatment of mesangial cells with LWDHW-containing serum improved the podocyte injury-inducing effects of the supernatant secreted by aIgA1-treated mesangial cells.

### Treatment With LWDHW-Containing Serum Can Improve aIgA1-Treated MCS-Induced Podocyte Injury

**FIGURE 2:**
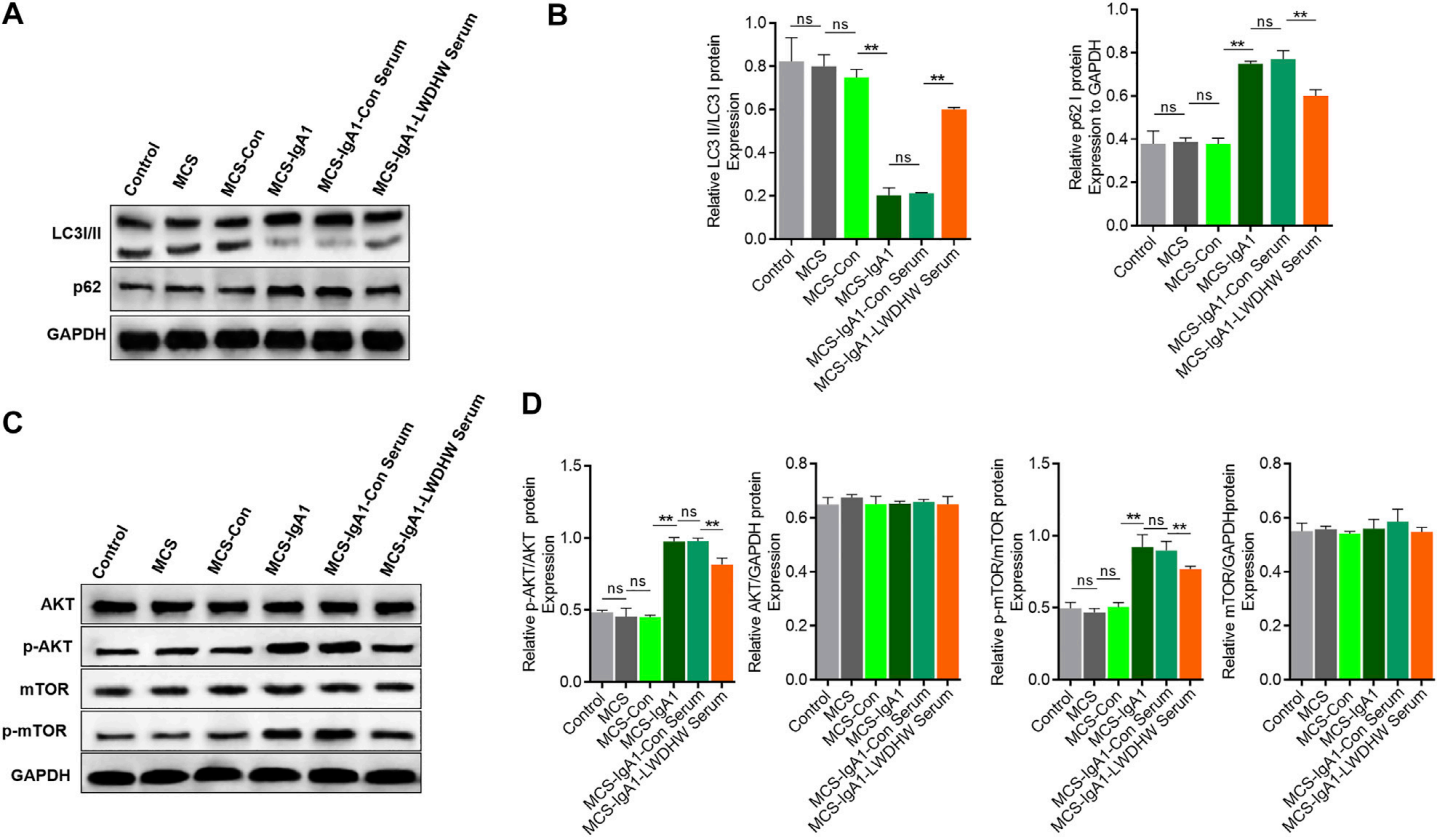
The effects of the supernatant of mesangial cells treated with Liuwei Dihuang pills and aIgA1 on the autophagy level and PI3K-Akt-mTOR pathway of podocytes were detected. **(A)** The level of autophagy of podocytes treated with mesangial cell supernatant for 24 h was detected by WB. **(B)** Quantification map of **(A)**. **(C)** Western Blot to detect the activation of PI3K-Akt-mTOR pathway in podocytes treated with Mesangial cell supernatant for 24 h. **(D)** Figure **(C)** Quantization diagram of WB strip. Control group: human podocytes without any treatment, MCS group: human podocytes treated with supernatant secreted by without any treatment Mesangial cells, MCS-Con group: human podocytes treated with supernatant secreted by Mesangial cells treated with solvent, MCS-IgA1: Mesangial cells treated with 25 μg/ml aIgA1 for 24 h, and human podocytes treated with secretory supernatant, MCS-IgA1-ConSerum: Mesangial cells treated with 25 μg/ml aIgA1 and 20% control serum for 24 h, and human podocytes treated with secretory supernatant, MCS-IgA1-LWDHWSrum: Mesangial cells treated with 25 μg/ml aIgA1 and 20% Liuwei Dihuang pills containing serum for 24 h, and human podocytes treated with secretory supernatant. ** it means *p* < 0.01, and ns means *p* > 0.05.

Autophagy plays an important role in regulating podocyte function (Tagawa et al., 2016), and LWDHW regulates renal autophagy levels (Liu 2019). To determine whether the decrease in podocyte injury induced by the supernatant of mesangial cells treated with LWDHW and aIgA1 was related to changes in autophagy levels, we evaluated the changes in the intracellular autophagy in podocytes treated with different MCSs using western blotting. Compared to the control group, there was no significant change in the LC3II/I and p62 content in the MCS group and no significant difference in the LC3II/I and p62 content between podocytes of the MCS and MCS-Con groups. Compared to the MCS-con group, the LC3II/I ratio in podocytes in the McS-IgA1 group significantly decreased, whereas the p62 content was upregulated in this group (*p* < 0.05, Figures 2A,B). This indicates that the MCS alone does not affect podocyte autophagy levels, but aIgA1-treated MCS can reduce podocyte autophagy levels. In addition, compared to the MCS-IgA1 group, there was no significant change in in autophagy levels in podocytes in groups that received MCS treated with 20% control serum and aIgA1 (Figures 2A,B). This indicated that treatment of mesangial cells with control serum did not reverse the inhibitory effects of aIgA1-treated MCS on podocyte autophagy levels. Compared to the MCS-IgA1-ConSerum group, podocyte autophagy levels were upregulated in groups treated with 20% LWDHW-containing serum and aIgA1-treated MCS (Figures 2A,B). Therefore, treatment of mesangial cells with LWDHW-containing serum reversed the inhibitory effects of the supernatants secreted by aIgA1-treated mesangial cells against podocyte autophagy.

In addition, western blotting showed that there was no significant change in Akt and mTOR phosphorylation levels in the MCS group compared to in the control group, there was no significant difference in the podocyte p-Akt and p-mTOR content between the MCS and MCS-Con groups. Akt and mTOR phosphorylation levels in podocytes in the MCS-IgA1 group were significantly higher than those in podocytes in the MCS-Con group (*p* < 0.05, Figures 2C,D). This result indicates that the MCS alone cannot affect the PI3K-Akt-mTOR pathway in podocytes, but aIgA1-treated MCS can activate the PI3K-Akt-mTOR pathway in podocytes. In addition, compared to the MCS-IgA1 group, there was no significant change in PI3K-Akt-mTOR pathway activity in podocytes in groups that received MCS treated with 20% control serum and aIgA1 (Figures 2C,D). This result indicates that mesangial cells treated with the control serum did not reverse the effects of aIgA1-treated MCS on PI3K-Akt-mTOR activation in podocytes. Compared to the MCS-IgA1-ConSerum group, there was a decrease in Akt and mTOR phosphorylation levels in podocytes in groups treated with 20% LWDHW-containing serum and aIgA1-treated MCS (Figures 2C,D). These results indicate that treatment of mesangial cells with LWDHW-containing serum improved the podocyte PI3K-Akt-MTOR pathway activation effects of the supernatant secreted by aIgA1-treated mesangial cells. In addition, intragastric administration of LWDHW significantly inhibited PI3K-Akt-mTOR pathway activation in the kidney tissues of IgAN model mice (Supplementary Figure S1). This observation suggests that the supernatant secreted by aIgA1-treated mesangial cells decreases podocyte autophagy levels by activating the PI3K-Akt-mTOR pathway in podocytes, which may lead to podocyte injury. LWDHW-containing serum inhibited PI3K-Akt-mTOR pathway activation and podocyte autophagy induced by aIgA1-treated MCS, thereby alleviating podocyte injury.

### Combination of LWDHW-Containing Serum and Rapamycin Can Reduce the Induction of aIgA1-Treated MCS-Induced Podocyte Injury

**FIGURE 3:**
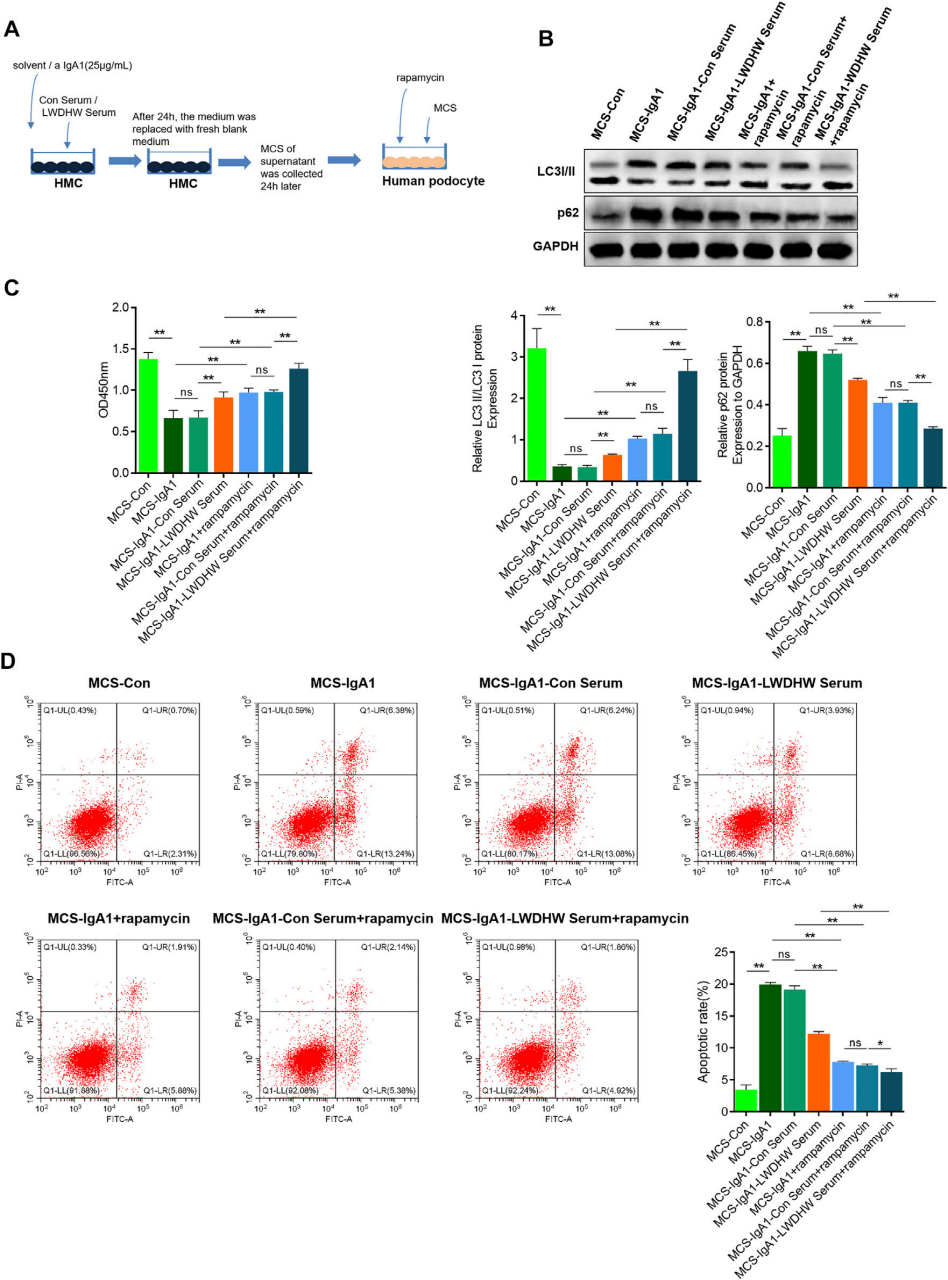
The effects of drug-containing serum, aIgA1 treated mesangial cell supernatant and autophagy inducer on popocyte injury were detected. **(A)** Cell treatment process. **(B)** WB to detect the level of autophagy of podocytes treated with Mesangial cell supernatant and autophagy inducer rapamycin for 24 h. **(C)** CCK8 to detect podocyte activity treated with Mesangial cell supernatant and autophagy inducer rapamycin for 24 h. **(D)** Flow cytometry to detect the level of podocyte apoptosis treated with Mesangial cell supernatant and autophagy inducer rapamycin for 24 h. LWDHW means Liuwei Dihuang pills. Control group: human podocytes without any treatment. MCS group: Human podocytes treated with supernatant secreted by untreated Mesangial cells. MCS-Con group: human podocytes treated with supernatant secreted by Mesangial cells treated with solvent. MCS-IgA1: Mesangial cells treated with 25 μg/ml aIgA1 for 24 h, and human podocytes treated with secretory supernatant. MCS-IgA1-ConSerum: Mesangial cells treated with 25 μg/ml aIgA1 and 20% control serum for 24 h, and human podocytes treated with secretory supernatant. MCS-IgA1-LWDHWSrum: Mesangial cells treated with 25 μg/ml aIgA1 and 20% Liuwei Dihuang pills containing serum for 24 h, and human podocytes treated with secretory supernatant. MCS-IgA1+rapamycin: The supernatant secreted by Mesangial cells treated with 25 μg/ml aIgA1 for 24 h and the autophagy inducer rapamycin were treated with human podocytes at the same time. MCS-IgA1-ConSerum + rapamycin: The supernatant secreted by Mesangial cells treated with 25 μg/ml aIgA1 and 20% control serum for 24 h, together with autophagy inducer rapamycin, simultaneously treated human podocytes. MCS-IgA1-LWDHWSerum + rapamycin: The supernatant secreted by Mesangial cells treated with 25 μg/ml aIgA1 and 20% Liuwei Dihuang Pill containing serum for 24 h was treated with rapamycin, an autophagy inducer, and human podocytes at the same time. ** means *p* < 0.01, ns means *p* > 0.05.

To investigate the effects of autophagy activation in combination with LWDHW on mesangial cell injury induced by the MCS, we collected the supernatant of mesangial cells treated with aIgA1 in combination with the control serum or drug-containing serum and used it to treat podocytes. The supernatant was simultaneously stimulated and treated with the autophagy activation agent rapamycin. Podocyte autophagy levels were determined by performing western blotting. Compared to the MCS-IgA1 group, there was an increase in the LC3II/I ratio and a decrease in the p62 content in podocyte groups treated with rapamycin. Compared to the MCS-IgA1-ConSerum + rapamycin and MCS-IgA1-LWDHW serum groups, the LC3II/I ratio and p62 content in podocytes in the MCS-IgA1-LWDHW serum + rapamycin group further increased and decreased, respectively (Figures 3A,B). These results suggest that podocytes treated with rapamycin in combination with MCS treated with the medicated serum further decreased the inhibitory effects of the supernatant secreted by aIgA1-treated mesangial cells on podocyte autophagy. The results of the CCK-8 assay and flow cytometry showed that compared to the MCS-IgA1 group, rapamycin treatment significantly enhanced podocyte activity. Compared to the MCS-IgA1 + rapamycin group, podocytes in the MCS-IgA1-Con serum group were treated with 20% control serum and aIgA1-treated MCS simultaneously, which did not affect podocyte activity. In addition, compared to the MCS-IgA1-Con serum + rapamycin and MCS-IgA1-LWDHW serum groups, podocyte activity was upregulated and apoptosis levels were decreased in the MCS-IgA1-LWDHWSerum + rapamycin group (Figures 3C,D). These findings indicate that the inhibitory effects of LWDHW in combination with rapamycin against podocyte autophagy and injury were significantly higher than those of LWDHW or rapamycin alone.

### LWDHW-Containing Serum Inhibits aIgA1-Treated MCS-Induced Podocyte Injury by Altering Exosome Function and Secretion in Mesangial Cells

**FIGURE 4:**
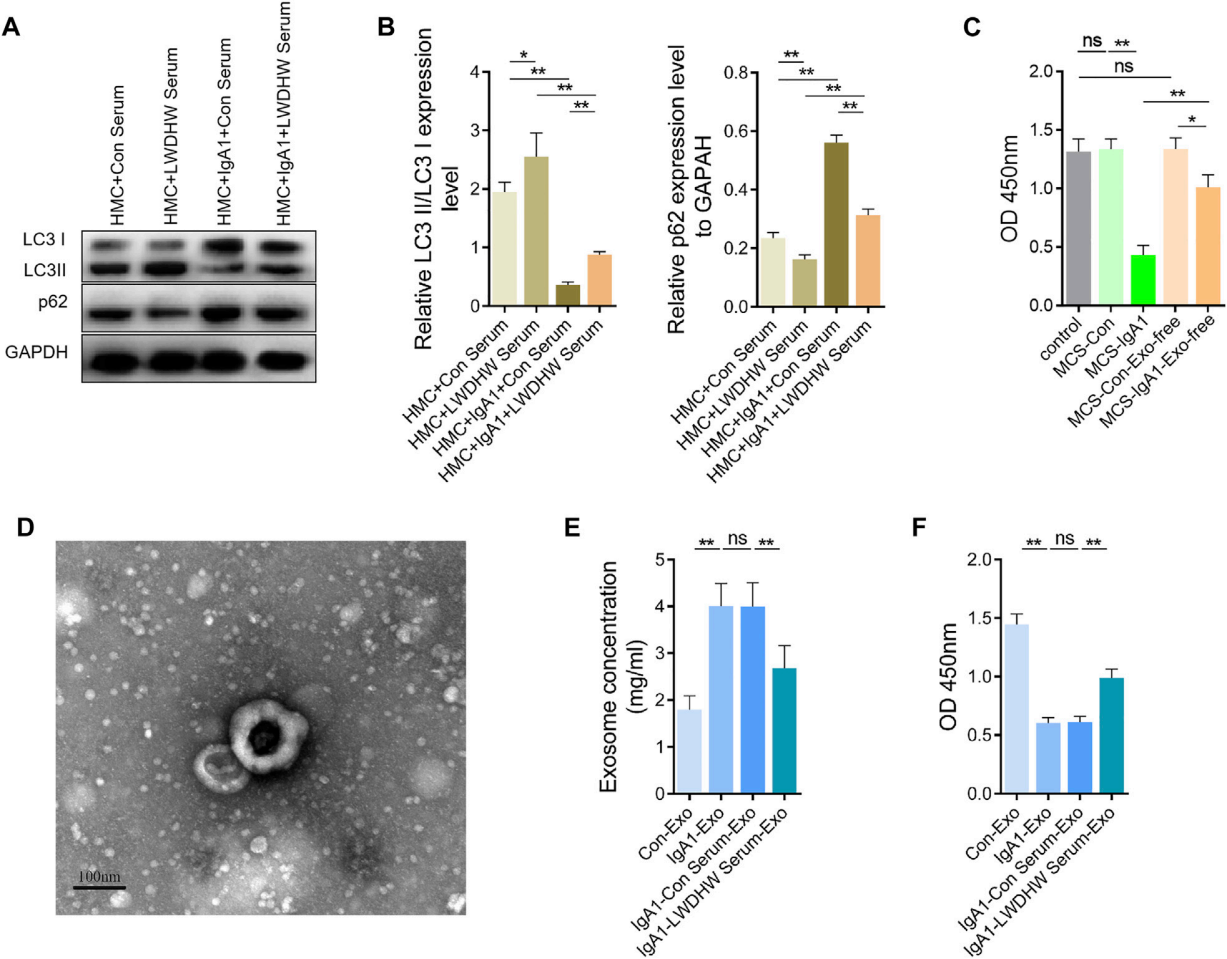
Effects of aIgA1 and Liuwei Dihuang Pill-containing serum on human mesangial cell autophagy level, human mesangial cell exosome secretion and mesangial cell exosomes on human podocyte activity. **(A)** WB assay of aIgA1 and LWDHW-containing serum on LC3II/I and p62 content in human mesangial cells. **(B)** Figure **(A)** Quantization diagram of WB strip. **(C)** CCK-8 assay to detect the effect of aIgA1 and LWDHW drug-containing serum treated HMCs supernatant, and exosome-free mesangial cell supernatant on podocyte activity. **(D)** The morphology of mesangial cell exosomes was observed under transmission electron microscopy. **(E)** The effect of aIgA1 and LWDHW-containing serum on mesangial cell exosome protein concentration was detected using BCA kit. **(F)** CCK-8 assay was performed to detect the effect of each group of HMC exosomes (25 μg/ml) on podocyte activity. ns means *p* > 0.05, * means *p* < 0.05, ** means *p* < 0.01.

In the absence of aIgA1 treatment, compared with the control serum treatment, LWDHW-containing serum treatment significantly increased the LC3II/I ratio and reduced the p62 content in HMCs (Figures 4A,B). Compared with that in the group without aIgA1 treatment, aIgA1 treatment significantly reduced the LC3II/I ratio and increased the p62 content in HMCs, aIgA1 treatment reduced the LC3II/I ratio in HMCs (*p* < 0.01, Figures 4A,B); additionally, p62 content was reduced in the aIgA1 and LWDHW serum-containing treatment groups compared to that in the aIgA1 and control serum treatment groups (*p* < 0.01, Figures 4A,B). These results indicated that the drug-containing serum of LWDHW blocked the inhibitory effect of aIgA1 on mesangial cells at the autophagy level.

After removing exosomes from the supernatant secreted by mesangial cells treated with aIgA1, the supernatant still reduced podocyte activity (*p* < 0.05), but the effects were much weaker than those of the whole supernatant (*p* < 0.01, Figure 4C). This result indicates that aIgA1 may induce podocyte injury mainly through mesangial cells exosomes.

Exosomes extracted from the supernatant of mesangial cells were round, with a diameter of approximately 100 nm under transmission EM (Figure 4D), which indicated that exosome extraction was successful. The concentration of exosomes secreted from mesangial cells after aIgA1 treatment was significantly increased, as assessed using the BCA assay (Figure 4E), whereas treatment with the serum-containing LWDHW reduced the effect of aIgA1 on promotion of exosome secretion from mesangial cells. The exosomes secreted by mesangial cells treated with aIgA1 reduced podocyte activity (*p* < 0.01, Figure 4F), which indicated that the exosomes of aIgA1-treated mesangial cells damaged the podocytes. Compared with the exosomes secreted by mesangial cells after treatment with the control serum and aIgA1, the exosomes secreted by mesangial cells treated with aIgA1 and LWDHW-containing serum showed improved inhibition of podocyte activity (*p* < 0.01, Figure 4F). LWDHW may inhibit the effect of aIgA1-treated MCS on podocyte injury mainly by altering mesangial cell exosome function and secretion.

### LWDHW in Combination With an Autophagy Inducer Can Significantly Inhibit the Progression of IgA Nephropathy in IgAN Model Mice

**FIGURE 5:**
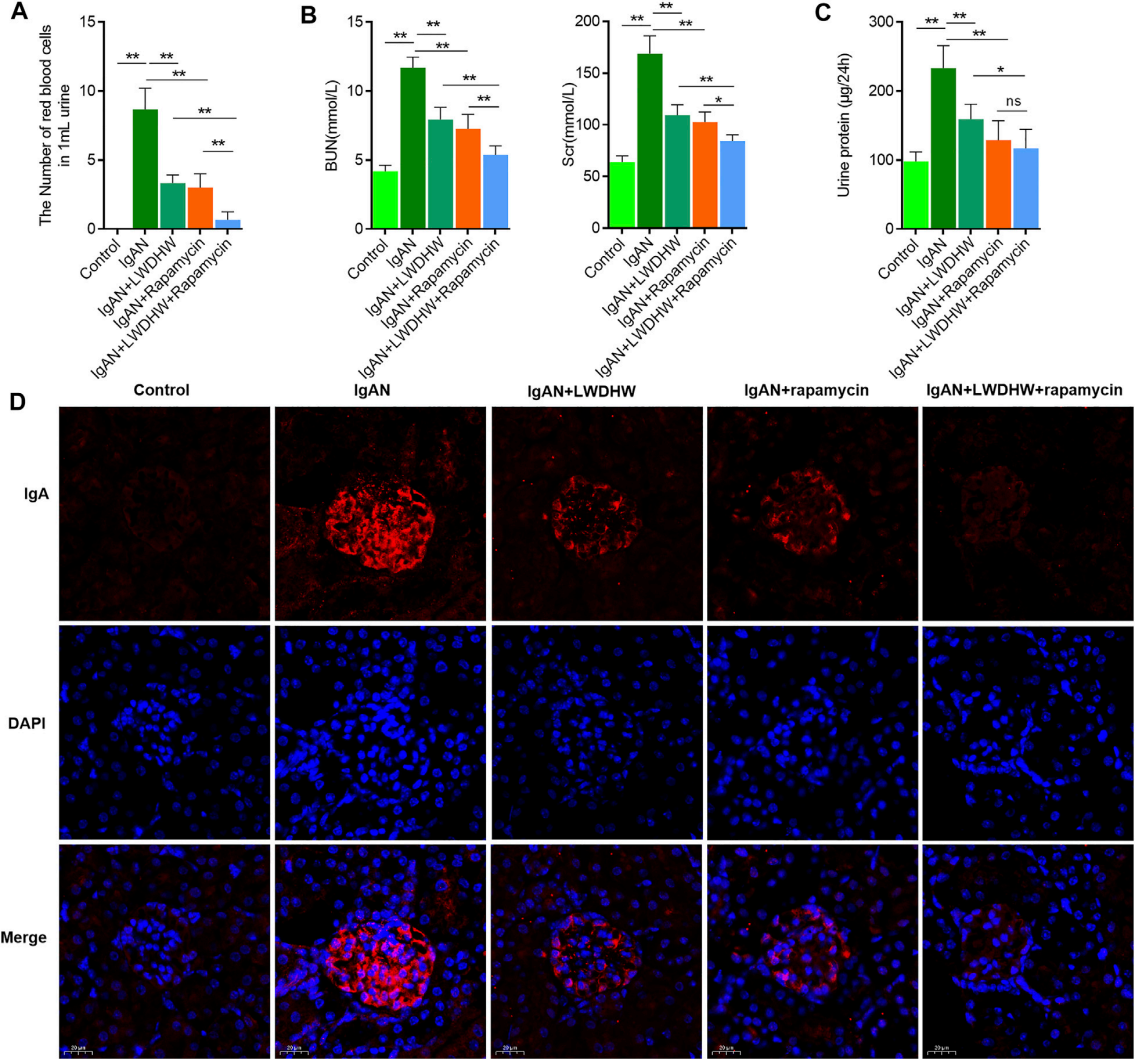
| Liuwei Dihuang Pill combined with autophagy inducer can significantly inhibit the progression of IgA nephropathy in IgAN model mice. **(A)** Observe the number of red blood cells in the urine of mice in each group under light microscope and make quantitative statistical analysis. **(B)** Detect the contents of serum creatinine and urea nitrogen in mice of each group by urea nitrogen detection kit and creatinine detection kit, respectively. **(C)** Detect the content of 24-h urine protein in mice of each group by BCA kit. **(D)** Detect the deposition of IgA in kidney tissue of mice in each group by tissue immunofluorescence method. LWDHW means Liuwei Dihuang pills, ** means p < 0.01, **means p < 0.05.

To clarify the effects of LWDHW in combination with rapamycin against IgAN progression, we constructed an IgAN mouse model. According to light microscopy analysis, compared to in control mice, the urine red blood cell count increased significantly and the serum creatinine and urea nitrogen content and 24-h urinary protein increased in IgAN model mice. This was accompanied by glomerular IgA deposition, indicating that the IgAN model was successfully established (Figures 5A–D). The effects of LWDHW and/or rapamycin on IgAN progression in IgAN model mice were evaluated. Compared to treatment with distilled water, treatment with LWDHW and rapamycin significantly reduced the urine red blood cell count, serum creatinine content, urea nitrogen, and 24-h urinary protein function in IgAN model mice. Among all treatments, LWDHW in combination with rapamycin showed the strongest inhibitory effects against the increased the urine red blood cell count, serum creatinine and urea nitrogen contents, and 24-h urinary protein in IgAN model mice (Figures 5A–C). These findings indicate that both LWDHW and rapamycin improve haematuria, renal function, and proteinuria, with their combination eliciting greater effects.

In the renal tissues, compared to treatment with distilled water, treatment with LWDHW and rapamycin significantly reduced glomerular IgA deposition in IgAN model mice. LWDHW in combination with rapamycin showed the highest inhibitory effects against glomerular IgA deposition in IgAN model mice (Figure 5D). Thus, LWDHW and rapamycin improved glomerular IgA deposition in the kidneys of IgAN model mice, and their combination elicited the strongest effects. Therefore, LWDHW and rapamycin, an autophagy inducer, improved IgAN progression in IgAN model mice. These effects may be related to inhibition of glomerular IgA deposition in the kidney tissues.

### LWDHW in Combination With Rapamycin Significantly Reduced Glomerular Pathological Changes and Autophagy in the Renal Tissues of IgAN Model Mice

**FIGURE 6:**
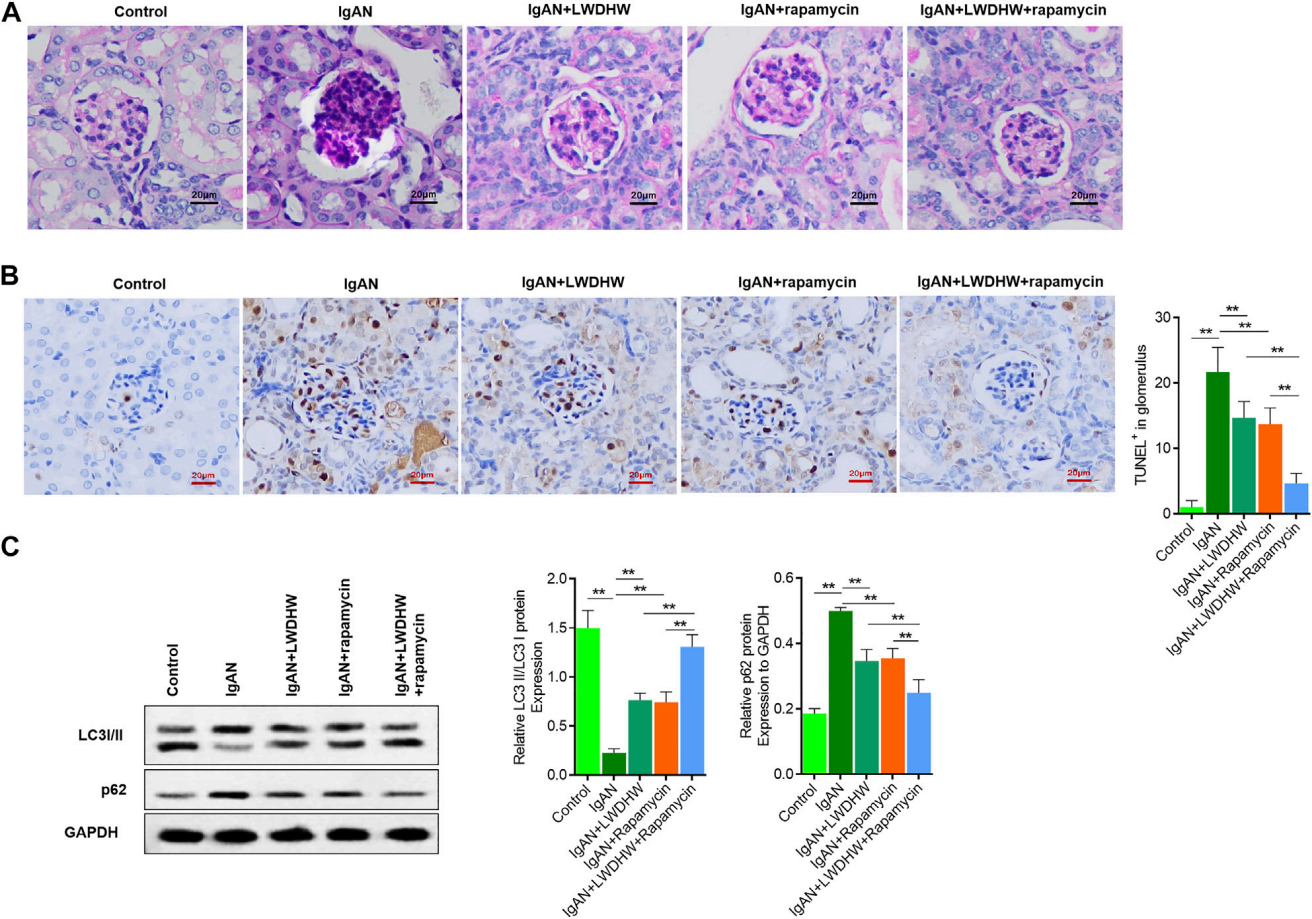
Liuwei Dihuang Pill can significantly inhibit glomerular pathological changes and apoptosis in kidney tissue of IgAN model mice and reverse autophagy down-regulation. **(A)** The pathological changes of glomerular structure and the deposition of polysaccharides in kidney tissue of mice in each group were observed by PAS staining. **(B)** TUNEL method was used to detect the level of apoptosis in renal tissue and glomeruli of mice in each group, and quantitative statistical analysis was made. **(C)** The proportion of LC3II/I and the content of p62 in kidney tissue of mice in each group were detected by Western Blot method. Take GAPDH as the internal reference, LWDHW for Liuwei Dihuang Pill. ** means *p* < 0.01, * means *p* < 0.05.

To clarify the effects of LWDHW and rapamycin on glomerular cytopathic changes in the renal tissues of IgAN model mice, periodic acid–Schiff and terminal deoxynucleotidyl transferase dUTP nick end labelling staining were used to evaluate changes in glomerular structure, polysaccharide deposition, and apoptosis in the renal tissues of mice in each group. Compared to control mice, the glomerular volume of IgAN model mice increased, accompanied by glomerular cell proliferation and polysaccharide deposition, and glomerular cell apoptosis levels were significantly increased. Compared to treatment with distilled water, treatment with LWDHW and rapamycin decreased the glomerular volume, glomerular cell proliferation and polysaccharide deposition, and glomerular cell apoptosis in IgAN model mice. Combined treatment with LWDHW and rapamycin showed the highest inhibitory effects (Figures 6A,B).

The kidney tissues of mice in each group were collected to evaluate autophagy levels using western blotting. Compared to control mice, the LC3II/I ratio and p62 content in the kidney tissues of IgAN model mice significantly decreased and increased, respectively, indicating decreased autophagy levels. Compared to treatment with distilled water, treatment with LWDHW and rapamycin reversed changes in the LC3II/I ratio and p62 content in the kidney tissues of IgAN model mice. Compared to treatment with LWDHW and rapamycin alone, treatment with LWDHW in combination with rapamycin led to greater decreases in the LC3II/I ratio and p62 content in the kidney tissues of IgAN model mice (*p* < 0.05, Figure 6C). These results indicate that both LWDHW and rapamycin significantly increased autophagy levels in the renal tissues of IgAN model mice, with their combination exerting greater effects against autophagy. LWDHW and rapamycin improved glomerular structural changes, glomerular cell apoptosis, and renal autophagy in IgAN model mice, and that their combination exhibits stronger regulatory effects.

## Discussion

IgAN is an important cause of end-stage renal disease in young people. However, the disease cannot be treated effectively and selectively, and most treatment strategies are based on controlling blood pressure and relieving proteinuria to slow disease progression (Leung et al., 2018; Huang and Xu 2021). Albuminuria is considered as the most widely and well-studied risk factor for IgAN progression to end-stage kidney disease (Thompson et al., 2019).

Podocytes, glomerular endothelial cells, and the glomerular basement membrane constitute the glomerular filtration barrier. Podocytes are the most essential part of the filtration unit and play an important role in maintaining the integrity of the glomerular filtration barrier (Patrakka and Tryggvason 2009). Recently, podocytes were identified as new targets for treating various albuminuria-related kidney diseases. Inhibition of podocyte autophagy in patients with IgAN is related to podocyte injury, apoptosis, dysfunction, and ultimately proteinuria (Ye et al., 2019). Autophagy is a highly lysosome-dependent intracellular process that widely occurs in eukaryotic cells as an adaptive metabolic process for degrading damaged or redundant organelles and biological macromolecules (Liu et al., 2016; Bhatia and Choi 2020). This process involves two main steps, autophagosome induction and autophagosome and lysosome fusion (Choi et al., 2013; Kim and Lee 2014). Two modes of autophagy exist in podocytes obtained by renal biopsy from patients with IgAN, and the podocytes from patients with IgAN contain more autophagic bodies than those of healthy subjects (Sato et al., 2009; Liang et al., 2016). In a study of the protective mechanism of triptolide in podocytes in IgAN model rats, triptolide reduced mesangial cell proliferation and IgA expansion and deposition (Liang et al., 2018). These findings suggest that autophagy is involved in the occurrence and development of IgAN, and that it may help to reduce IgA deposition during IgAN and prevent inflammation-related renal damage. Therefore, autophagy upregulation may be beneficial for treating IgAN.

LWDHW was recently shown to improve insulin resistance and correct lipid metabolic disorders, and is useful for treating diabetic nephropathy, hypoglycaemia, osteoporosis, and other diseases (He et al., 2007; Xu et al., 2017). However, the mechanism of LWDHW in IgAN treatment is unclear. Because the LWDHW formula contains a variety of components, among which polysaccharides and flavonoids exert nephroprotective functions, we previously analysed the physicochemical properties of LWDHW mainly by detecting the polysaccharide, flavonoid, and protein contents for drug quality control (Peng et al., 2020), which showed percentages of 52.11%, 0.99%, and 8.30%, respectively, in the filtrate of LWDHW.

In previous research of aIgA1 from patients with IgAN, conditioned media from mesangial cells incubated with aIgA1 from IgAN patients caused podocyte injury (Weide and Huber 2011). Therefore, we established a podocyte injury model induced by MCS after aIgA1 treatment and determined the effects of LWDHW on podocytes. The exosomes secreted by mesangial cells after aIgA1 treatment reduced podocyte activity, which indicated that podocyte injury was induced by mesangial cell exosomes. The LWDHW-containing serum reduced aIgA1-promoted exosome secretion from mesangial cells compared to that in the controls. Notably, the supernatant secreted by aIgA1-treated mesangial cells inhibited podocyte autophagy by activating the PI3K-Akt-mTOR pathway, and LWDHW was induced autophagy through the PI3K-Akt-mTOR pathway to inhibit aIgA1-treated mesangial cell-induced podocyte injury.

In our mouse model of IgAN, the urine red blood cell count of model mice was significantly increased, and the serum creatinine and urea nitrogen content and 24-h urinary protein were increased, accompanied by glomerular IgA deposition in the kidney tissues. After treatment with LWDHW, these effects were significantly reversed. LWDHW improved IgA deposition in the mouse glomeruli, which may be related to inhibition of IgA deposition in renal tissues. Moreover, LWDHW improved glomerular structural changes, glomerular cell apoptosis, and renal autophagy in IgAN model mice. In addition, both *in vitro* and *in vivo*, the effects of LWDHW in combination with rapamycin were significantly higher than those of each agent alone.

However, this study had some limitations. LWDHW is composed of several Chinese herbal medicines, other the involvement of other mechanisms that were not investigated in this study cannot be ruled out. Therefore, further studies and clinical trials are needed to verify our results.

## Conclusion

LWDHW may improve regulation of the PI3K-Akt-mTOR pathway and inhibit autophagy in podocytes by altering the function and secretion of glomerular mesangial cell-derived exosomes, thereby improving podocyte injury and inhibiting IgAN progression. Treatment using LWDHW in combination with rapamycin can further enhance podocyte autophagy, improve podocyte injury, and inhibit IgAN progression.

## Data Availability

The original contributions presented in the study are included in the article/Supplementary Material, further inquiries can be directed to the corresponding authors.

## Abbreviations

IgA, immunoglobulin A; IgAN, immunoglobulin A nephropathy; HMC, human mesangial cell; LWDHW, Liuwei Dihuang pills; aIgA1, aggregated IgA1; MCS, mesangial cell supernatant; PBS, phosphate-buffered saline.
