# Supplementary figures and images for: Liuwei Dihuang Pills Inhibit Podocyte Injury and Alleviate IgA Nephropathy by Directly Altering Mesangial Cell-Derived Exosome Function and Secretion (part 1 of 2)
Source: Front Pharmacol. 2022 Jul 11;13:889008. doi: 10.3389/fphar.2022.889008 (PMC9309816; doi:10.3389/fphar.2022.889008)

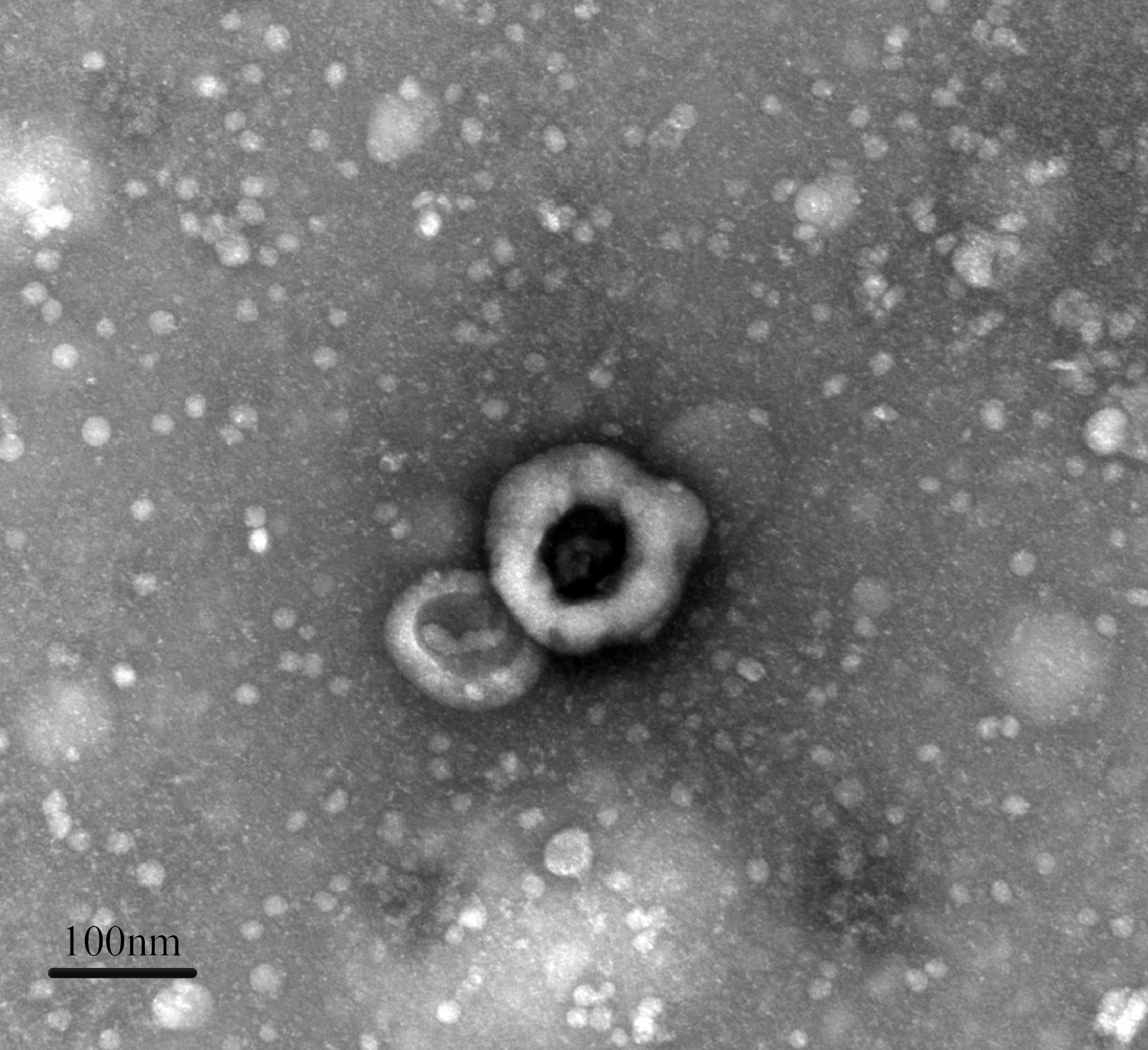

Supplement: Supplementary file 1 [file DataSheet3.ZIP › original data/Exosome transmission electron microscopy.tif]

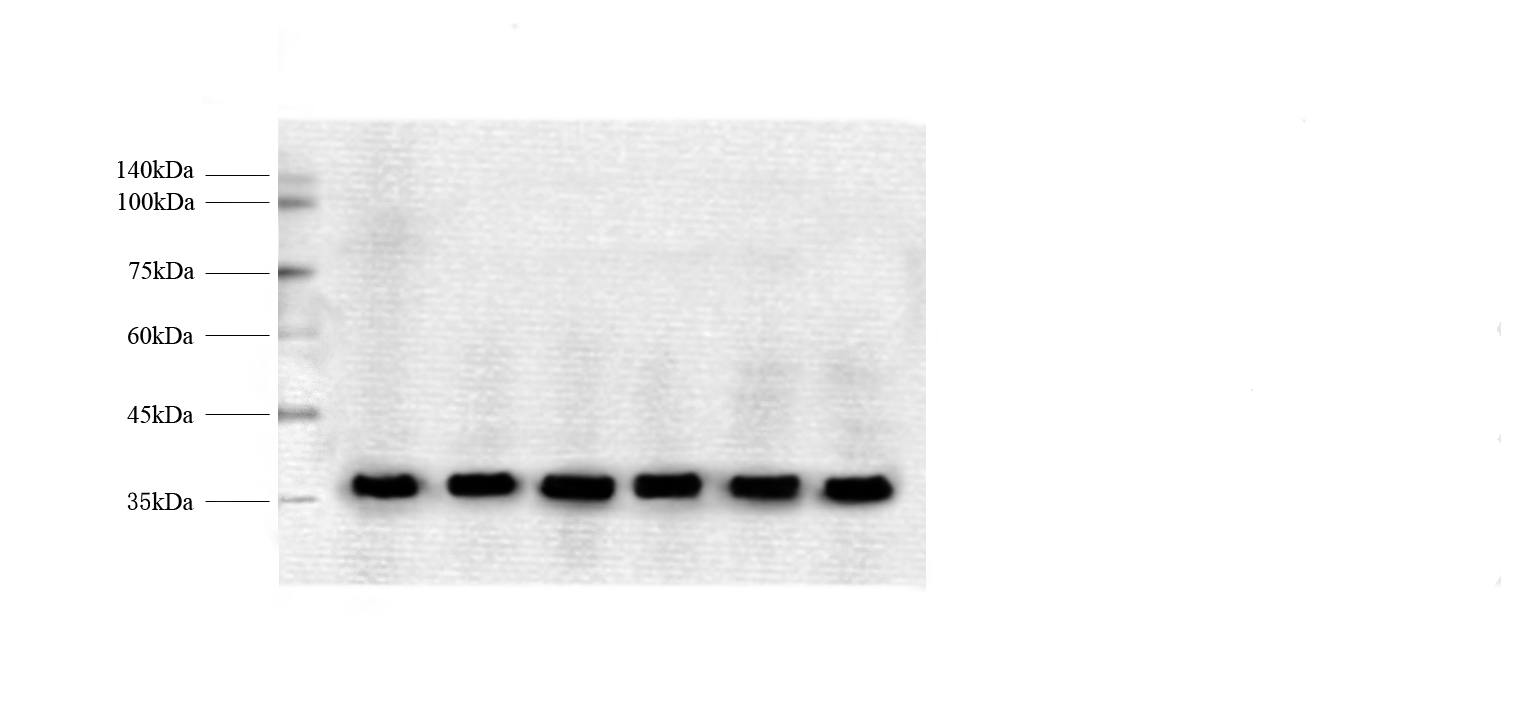

Supplement: Supplementary file 1 [file DataSheet3.ZIP › original data/Fig.2A/GAPDH-1.tif]

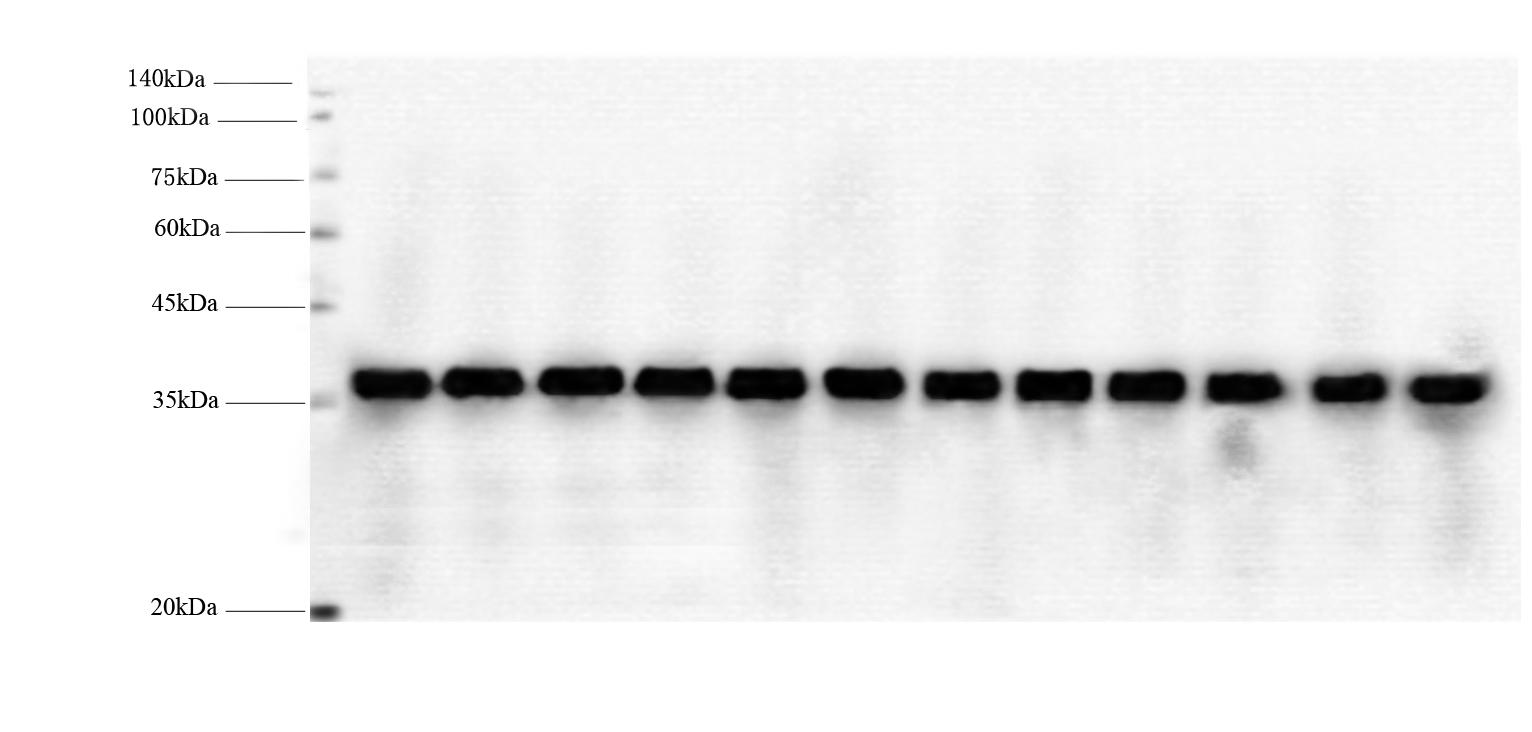

Supplement: Supplementary file 1 [file DataSheet3.ZIP › original data/Fig.2A/GAPDH.tif]

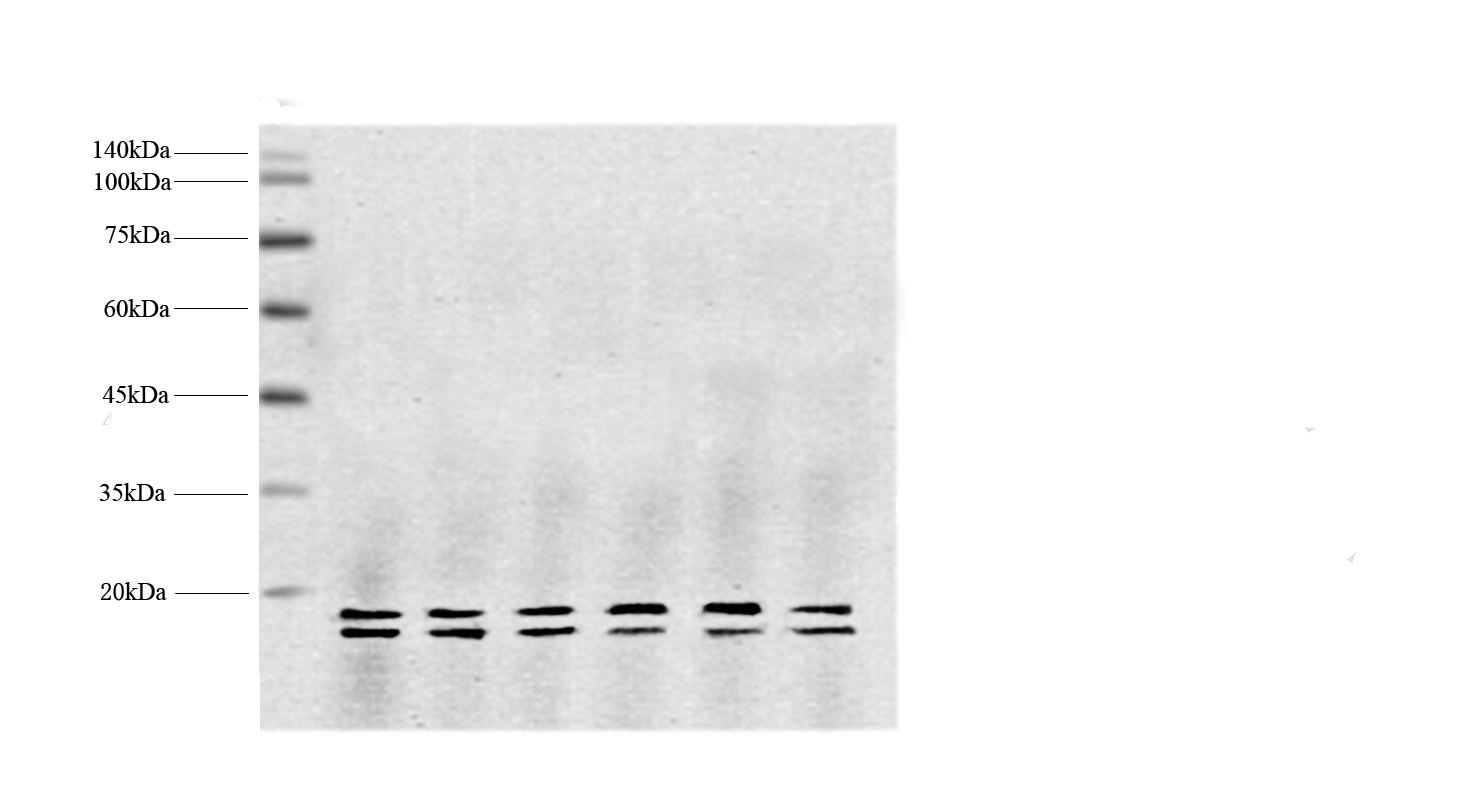

Supplement: Supplementary file 1 [file DataSheet3.ZIP › original data/Fig.2A/LC3-1.tif]

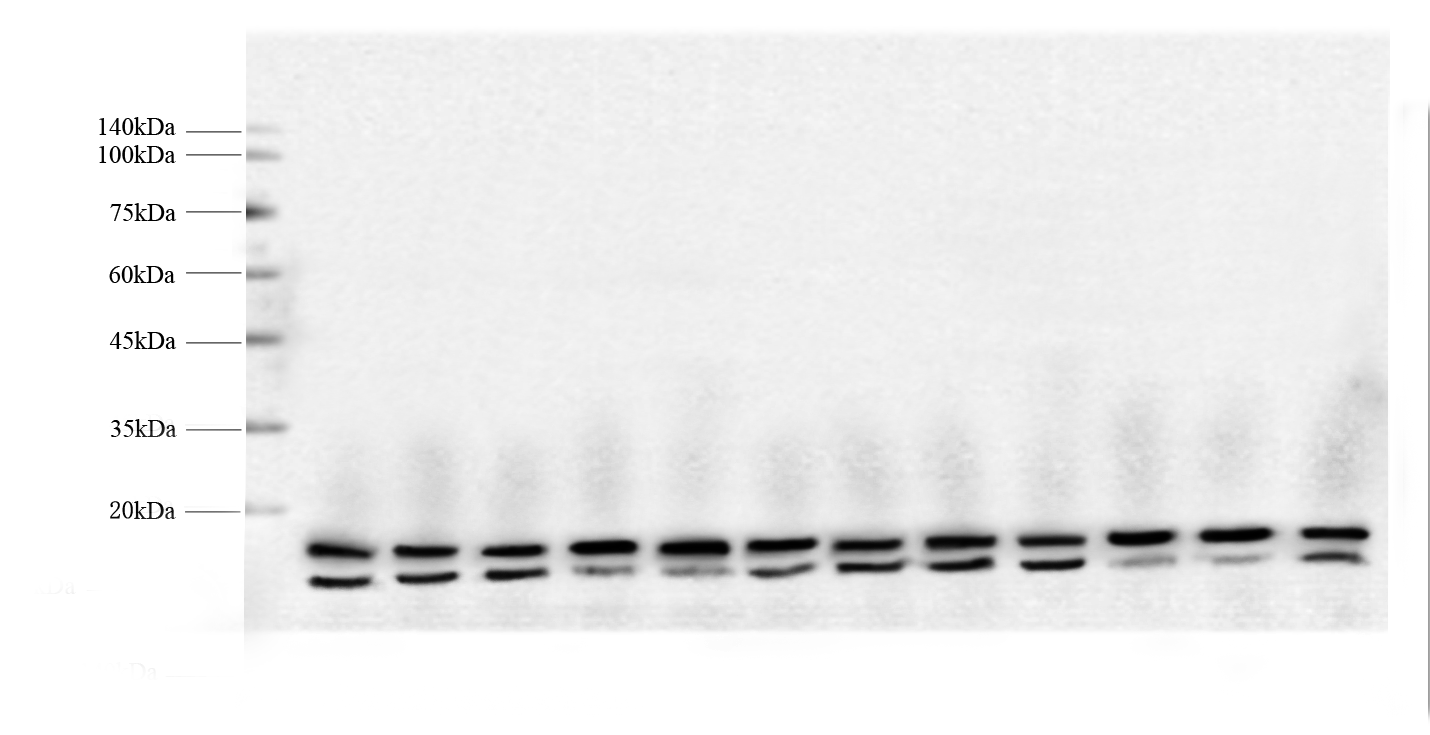

Supplement: Supplementary file 1 [file DataSheet3.ZIP › original data/Fig.2A/LC3.tif]

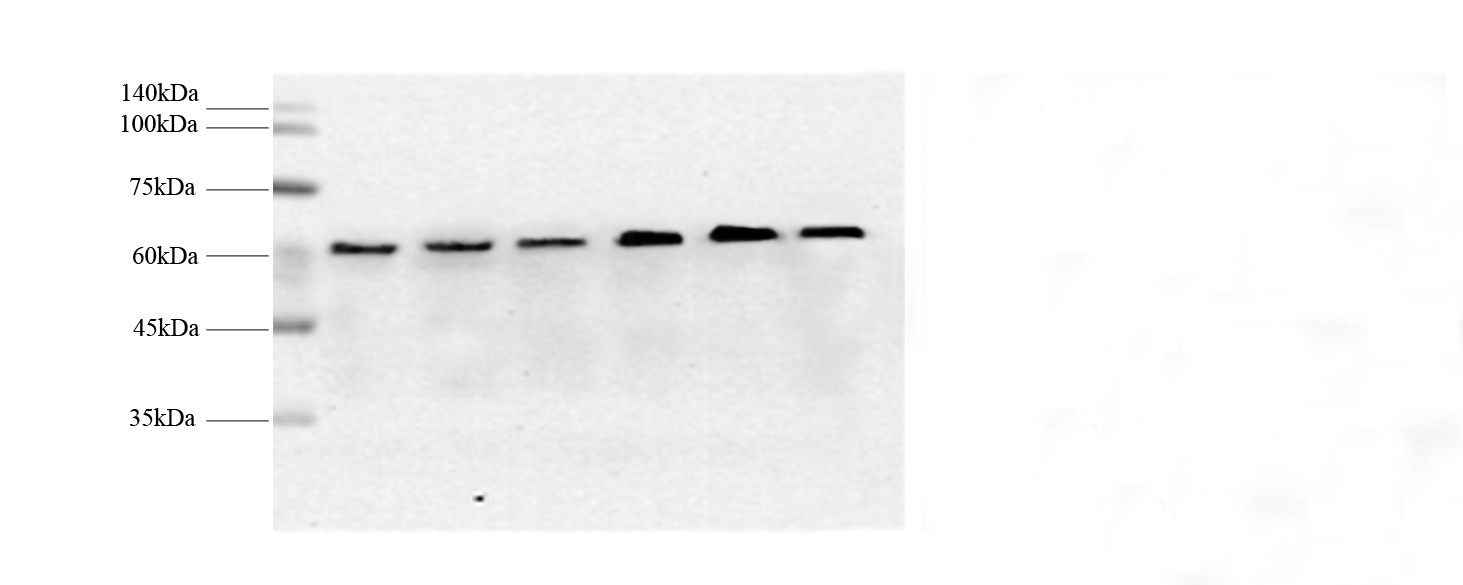

Supplement: Supplementary file 1 [file DataSheet3.ZIP › original data/Fig.2A/p62-1.tif]

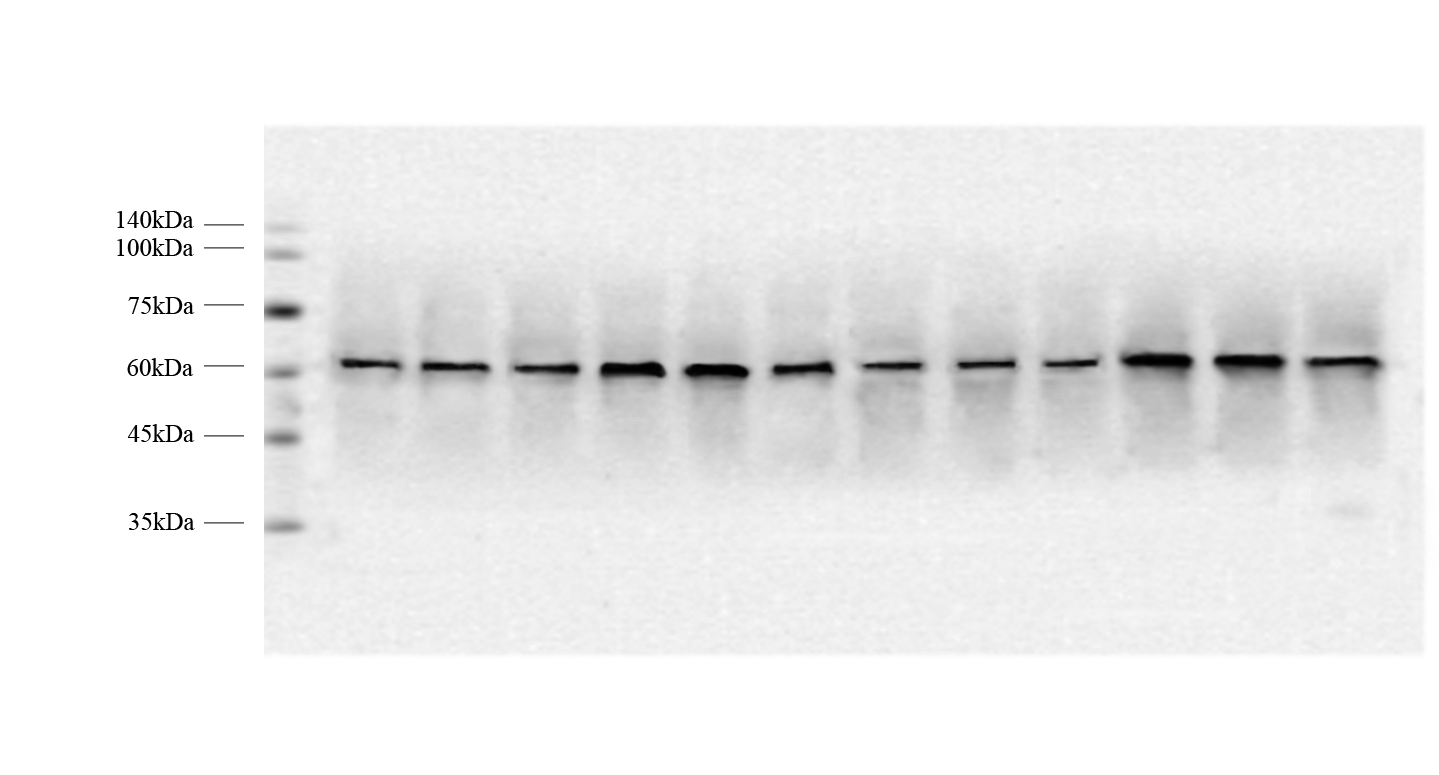

Supplement: Supplementary file 1 [file DataSheet3.ZIP › original data/Fig.2A/p62.tif]

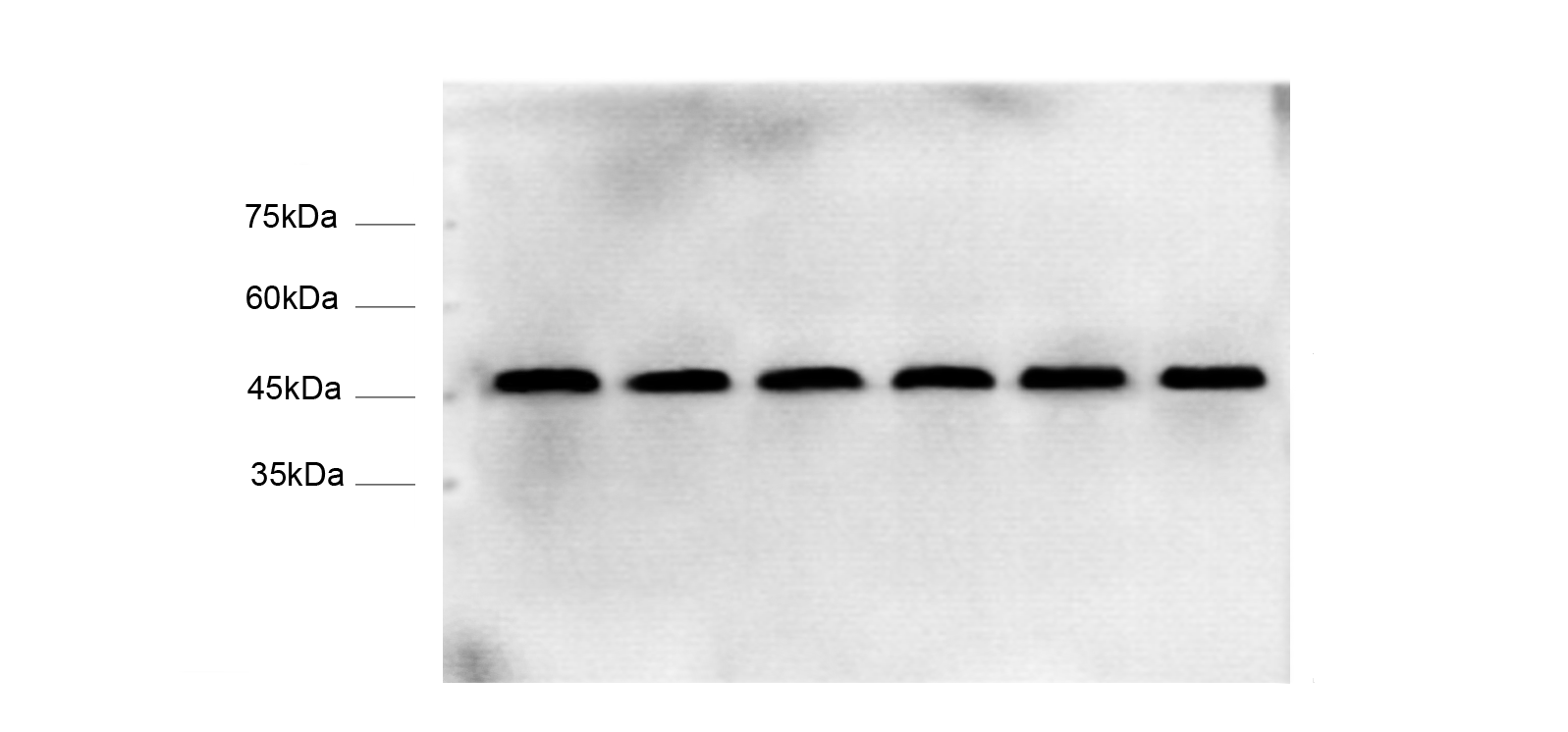

Supplement: Supplementary file 1 [file DataSheet3.ZIP › original data/Fig.2C/1-AKT-1.tif]

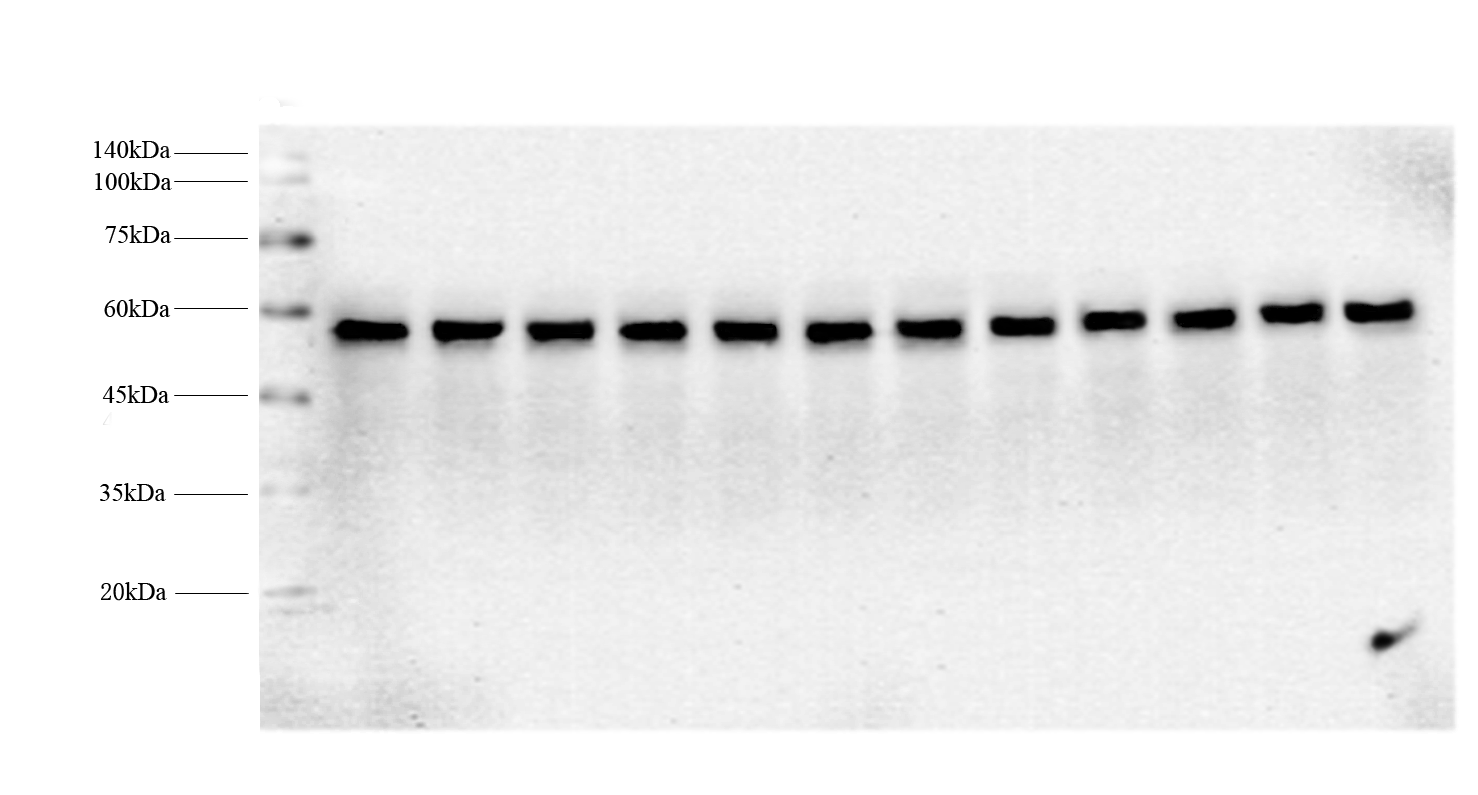

Supplement: Supplementary file 1 [file DataSheet3.ZIP › original data/Fig.2C/1-AKT.tif]

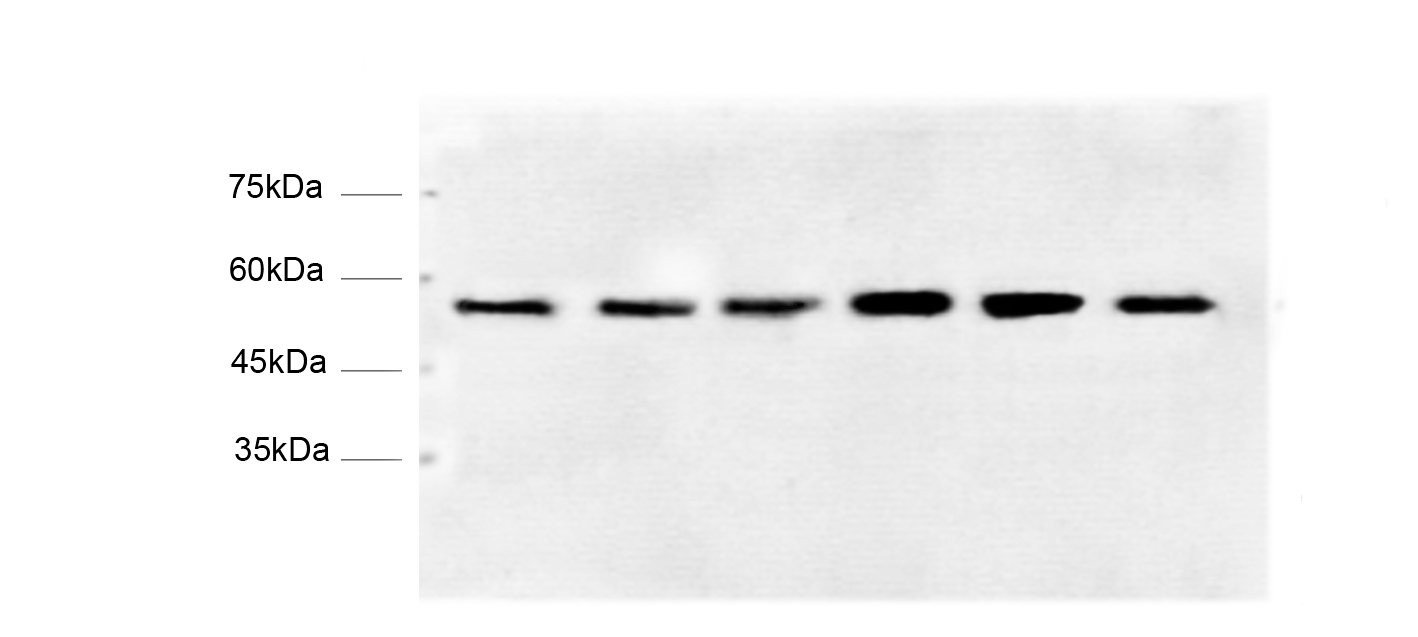

Supplement: Supplementary file 1 [file DataSheet3.ZIP › original data/Fig.2C/2-p-Akt-1.tif]

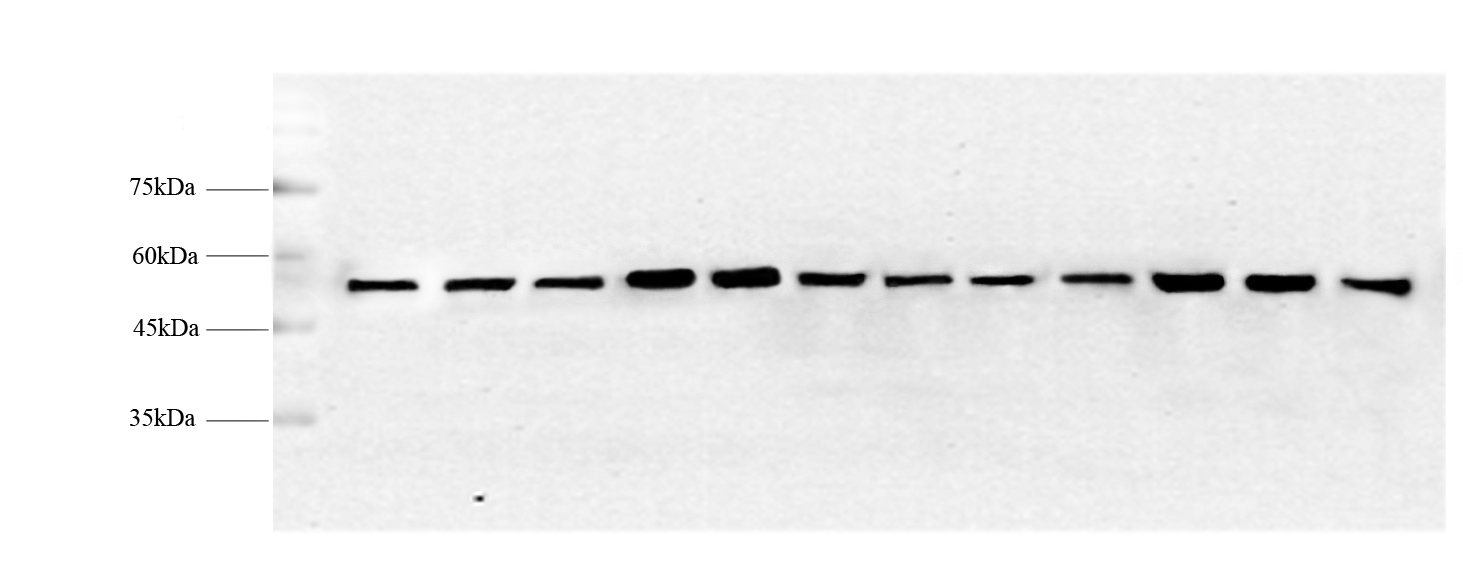

Supplement: Supplementary file 1 [file DataSheet3.ZIP › original data/Fig.2C/2-p-Akt.tif]

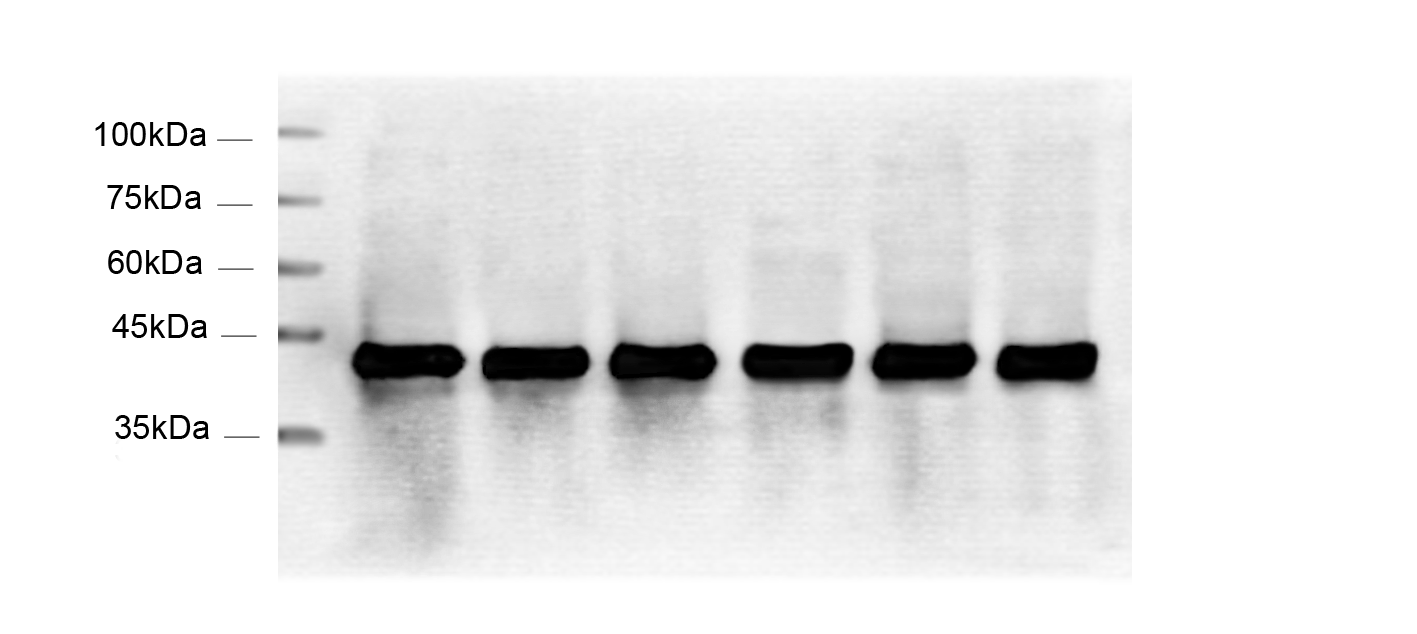

Supplement: Supplementary file 1 [file DataSheet3.ZIP › original data/Fig.2C/3-GAPDH-1.tif]

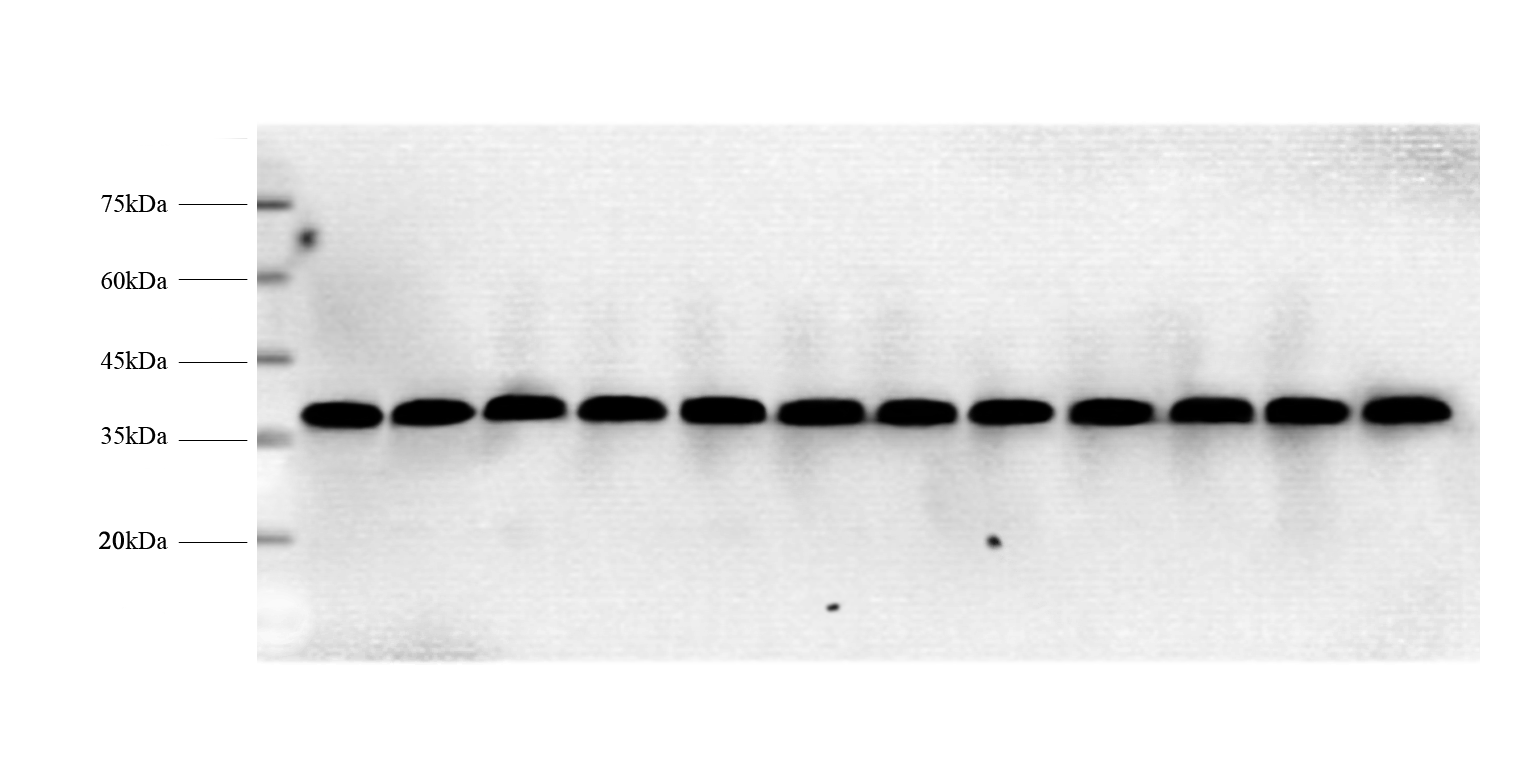

Supplement: Supplementary file 1 [file DataSheet3.ZIP › original data/Fig.2C/3-GAPDH.tif]

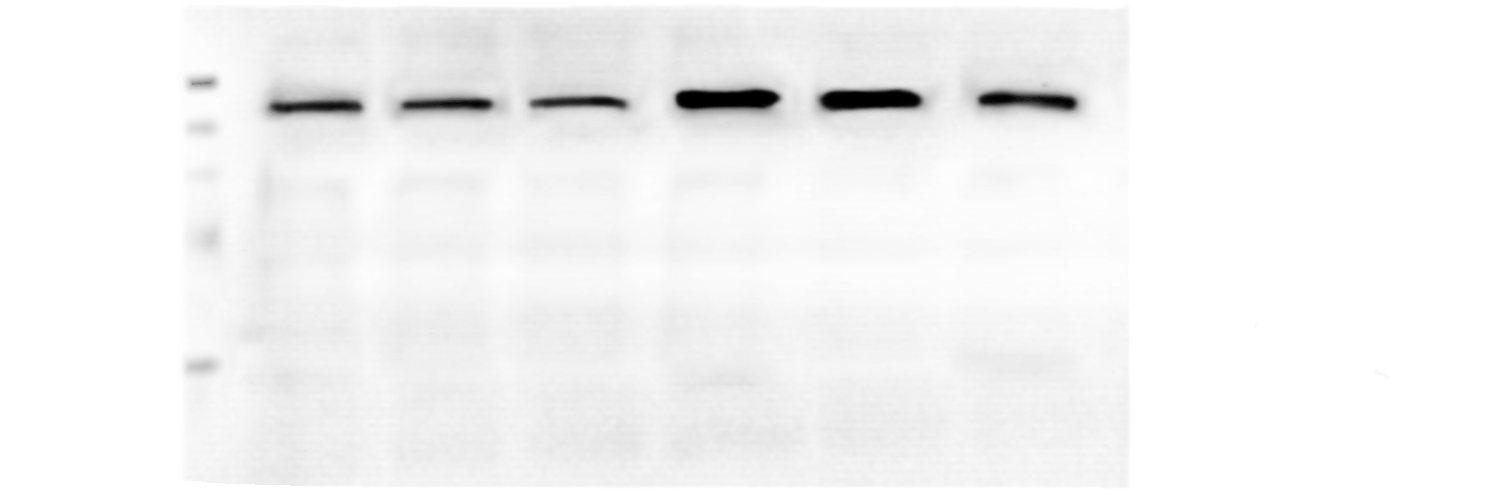

Supplement: Supplementary file 1 [file DataSheet3.ZIP › original data/Fig.2C/4-mTOR-1.tif]

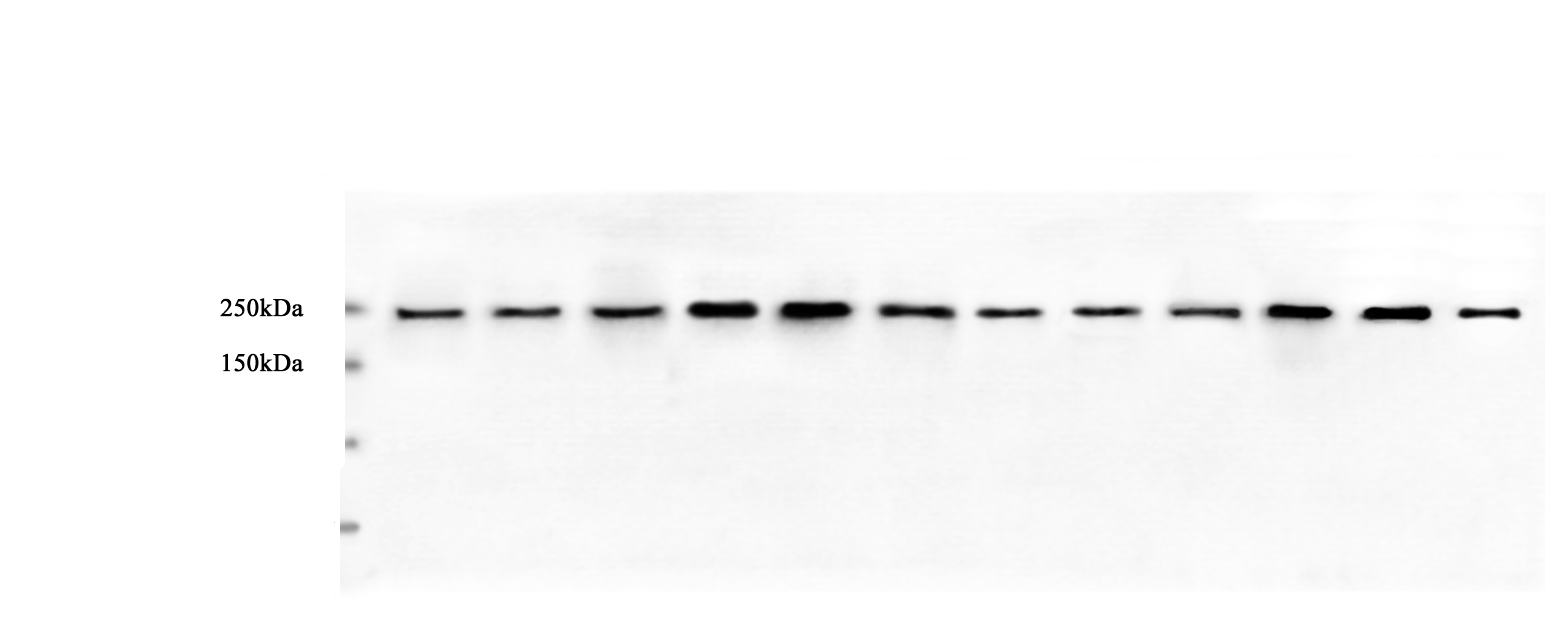

Supplement: Supplementary file 1 [file DataSheet3.ZIP › original data/Fig.2C/4-mTOR.tif]

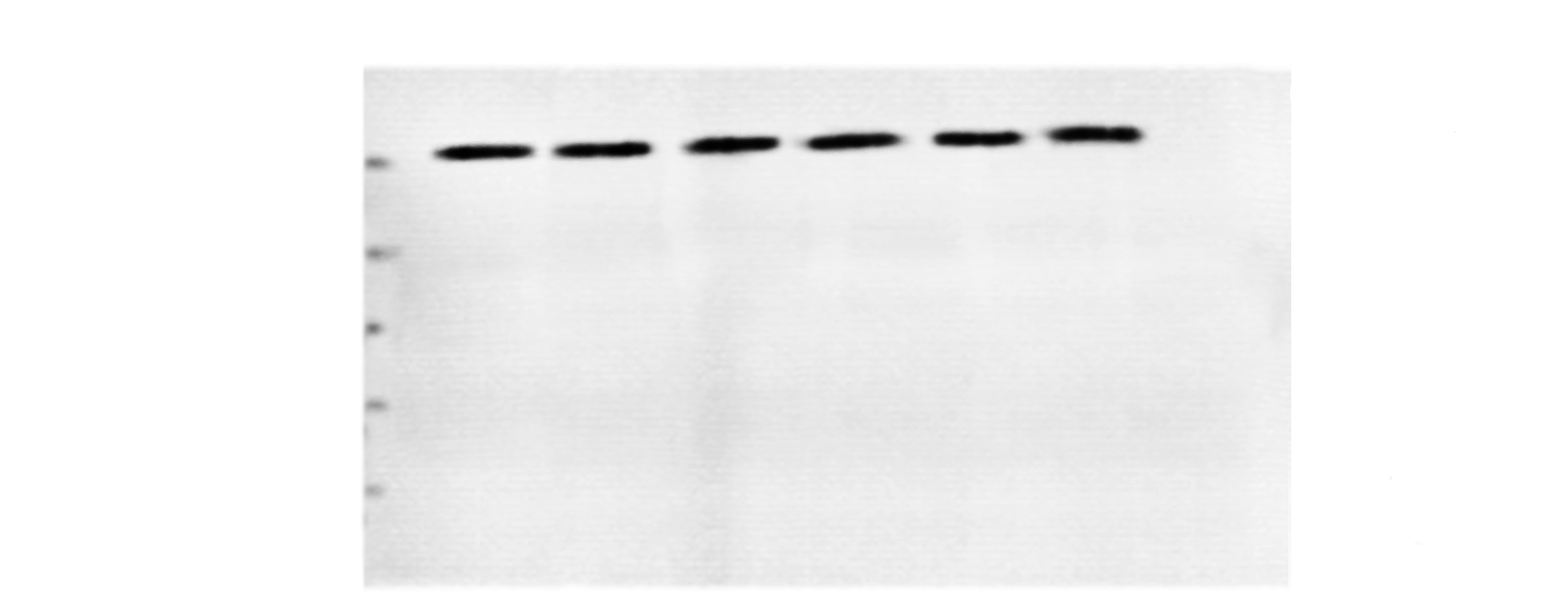

Supplement: Supplementary file 1 [file DataSheet3.ZIP › original data/Fig.2C/5-p-mTOR-1.tif]

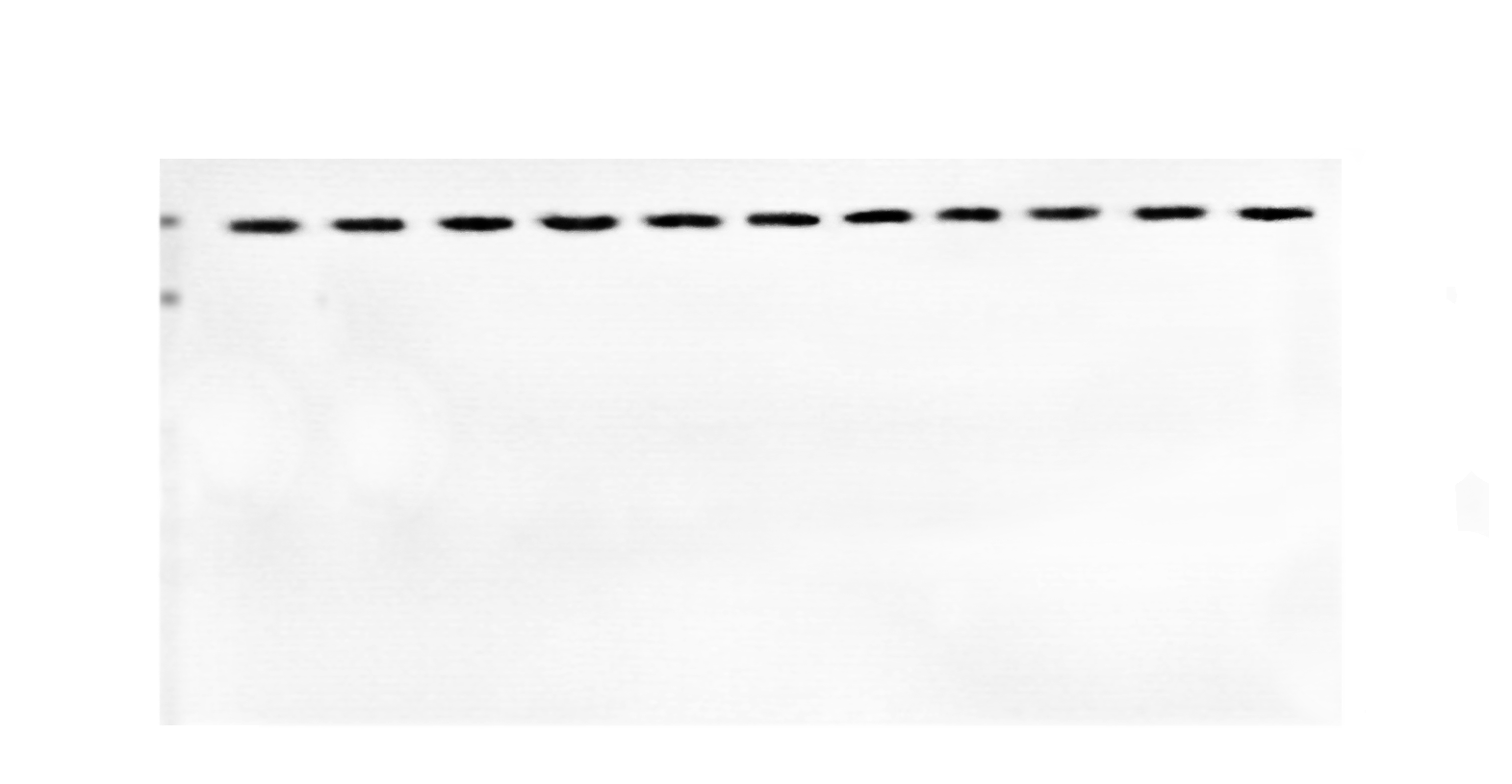

Supplement: Supplementary file 1 [file DataSheet3.ZIP › original data/Fig.2C/5-p-mTOR.tif]

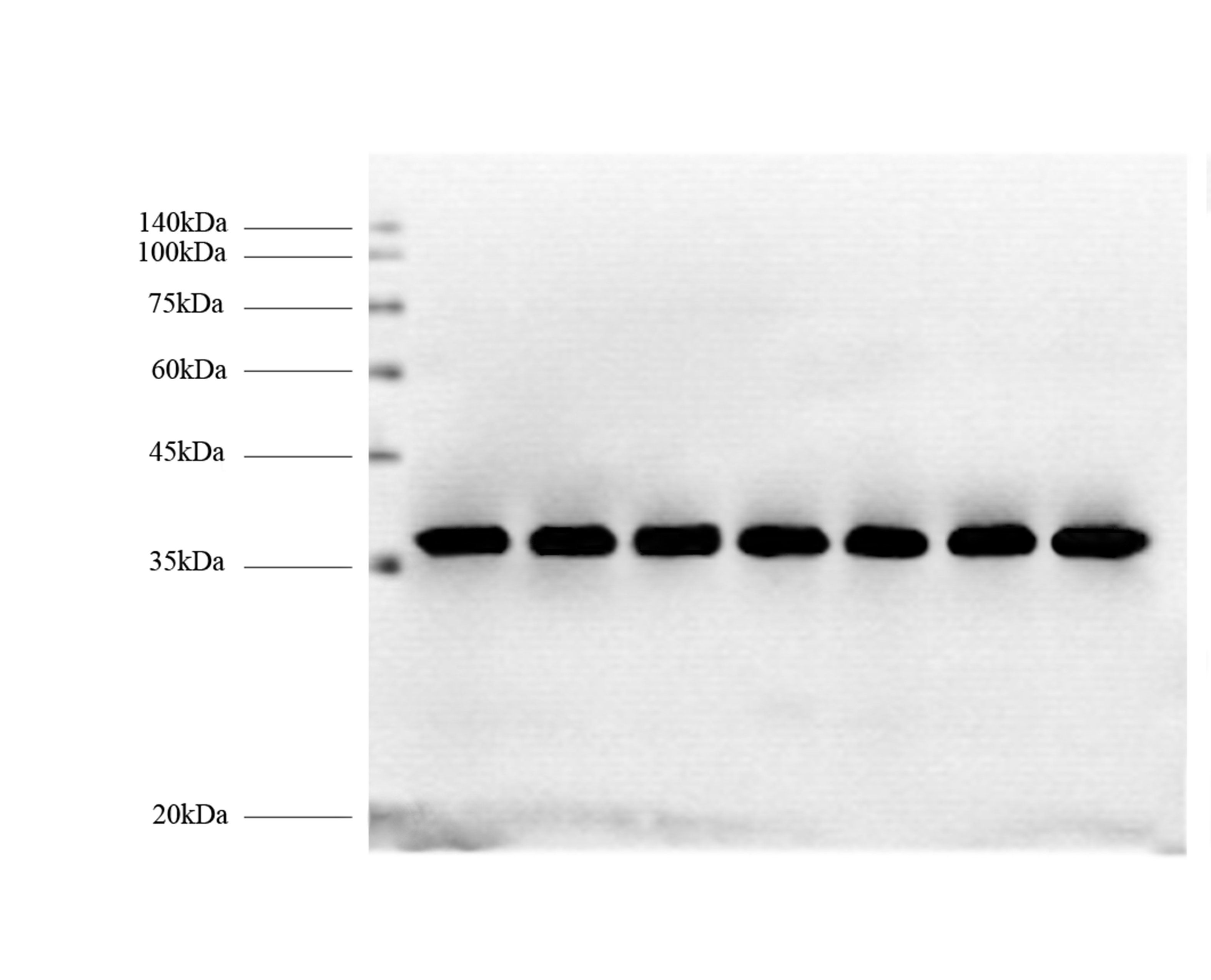

Supplement: Supplementary file 1 [file DataSheet3.ZIP › original data/Fig.3B/GAPDH-1.tif]

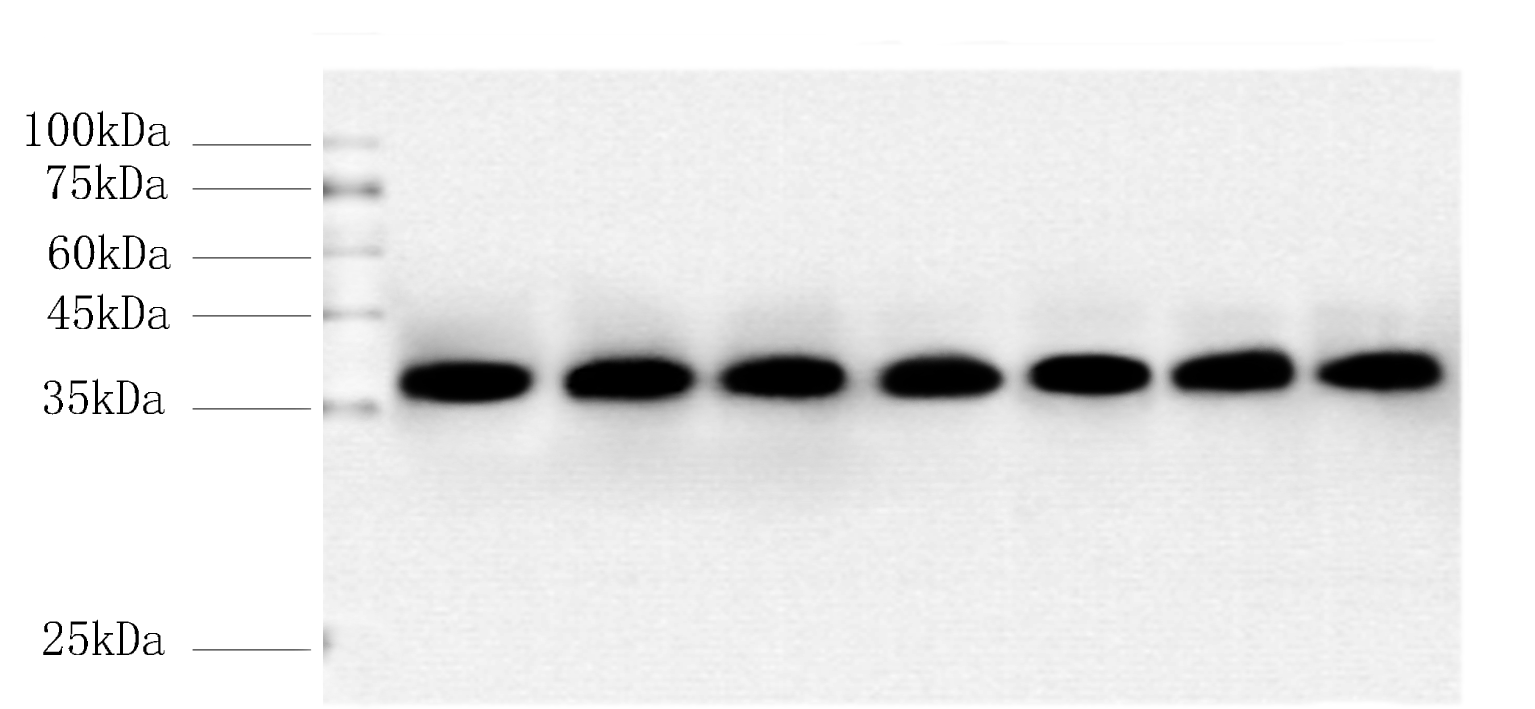

Supplement: Supplementary file 1 [file DataSheet3.ZIP › original data/Fig.3B/GAPDH-2.tif]

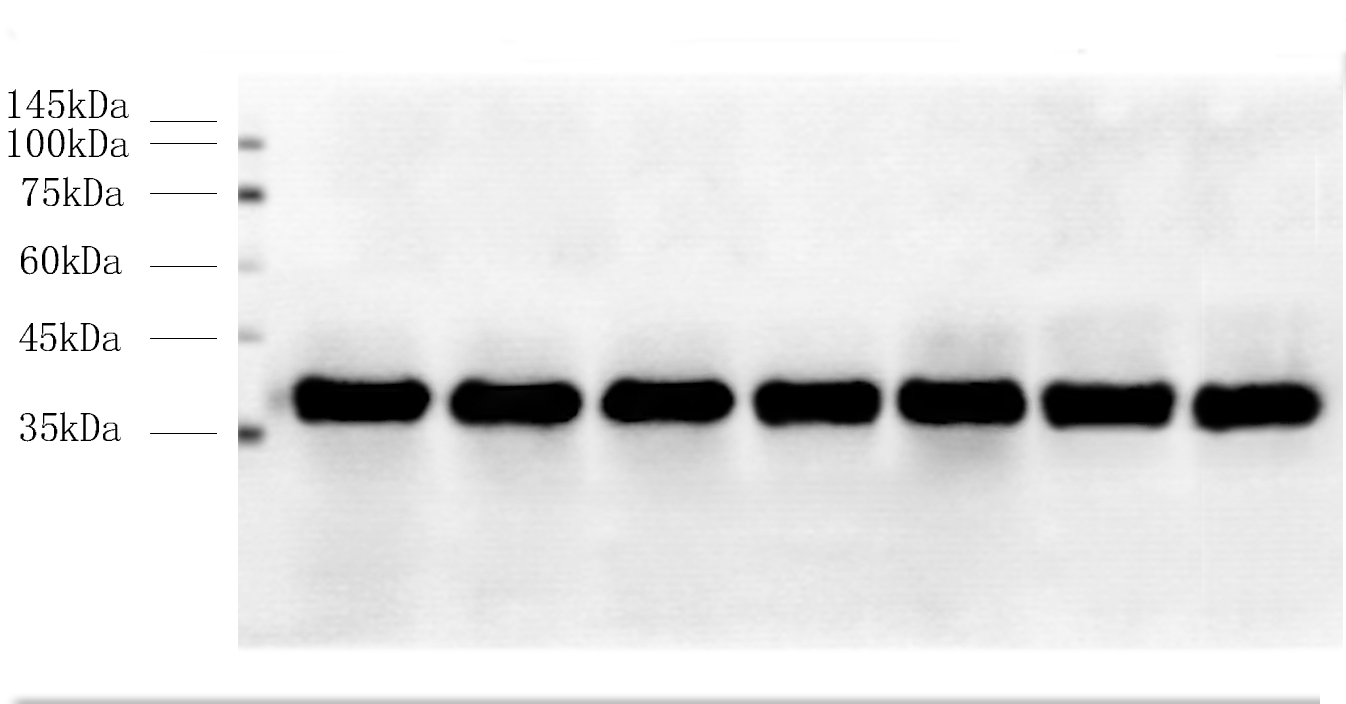

Supplement: Supplementary file 1 [file DataSheet3.ZIP › original data/Fig.3B/GAPDH-3.tif]

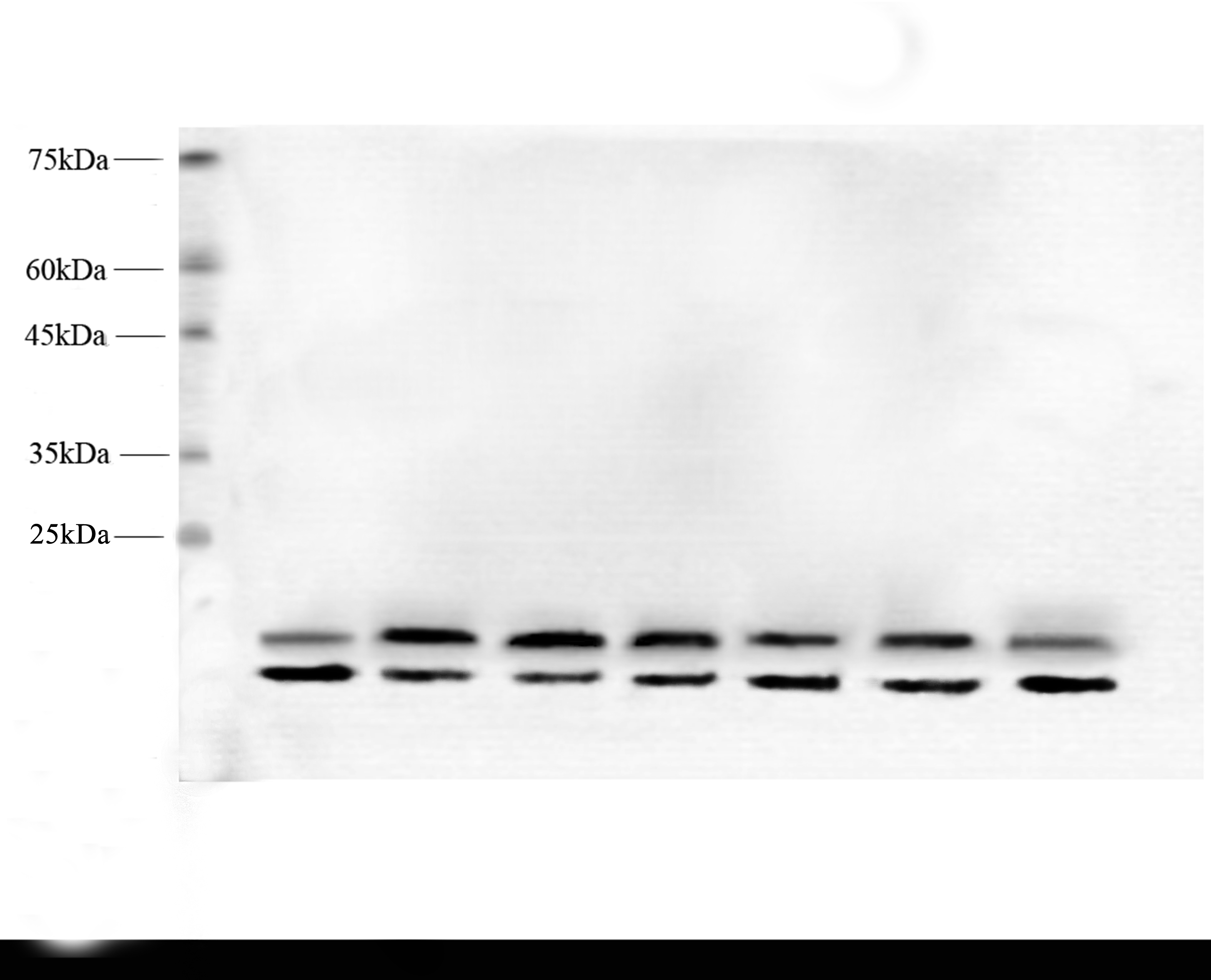

Supplement: Supplementary file 1 [file DataSheet3.ZIP › original data/Fig.3B/LC3-1.tif]

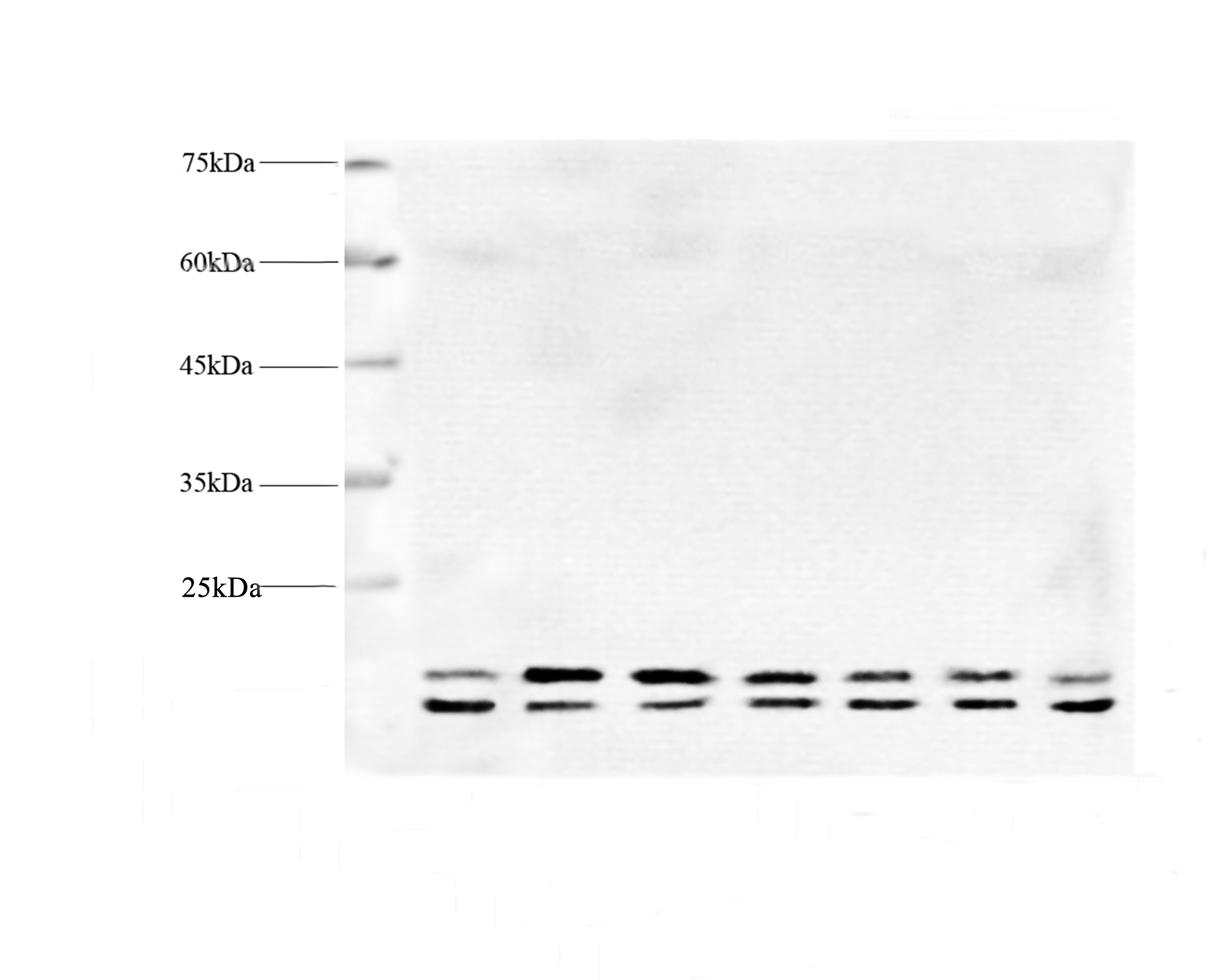

Supplement: Supplementary file 1 [file DataSheet3.ZIP › original data/Fig.3B/LC3-2.tif]

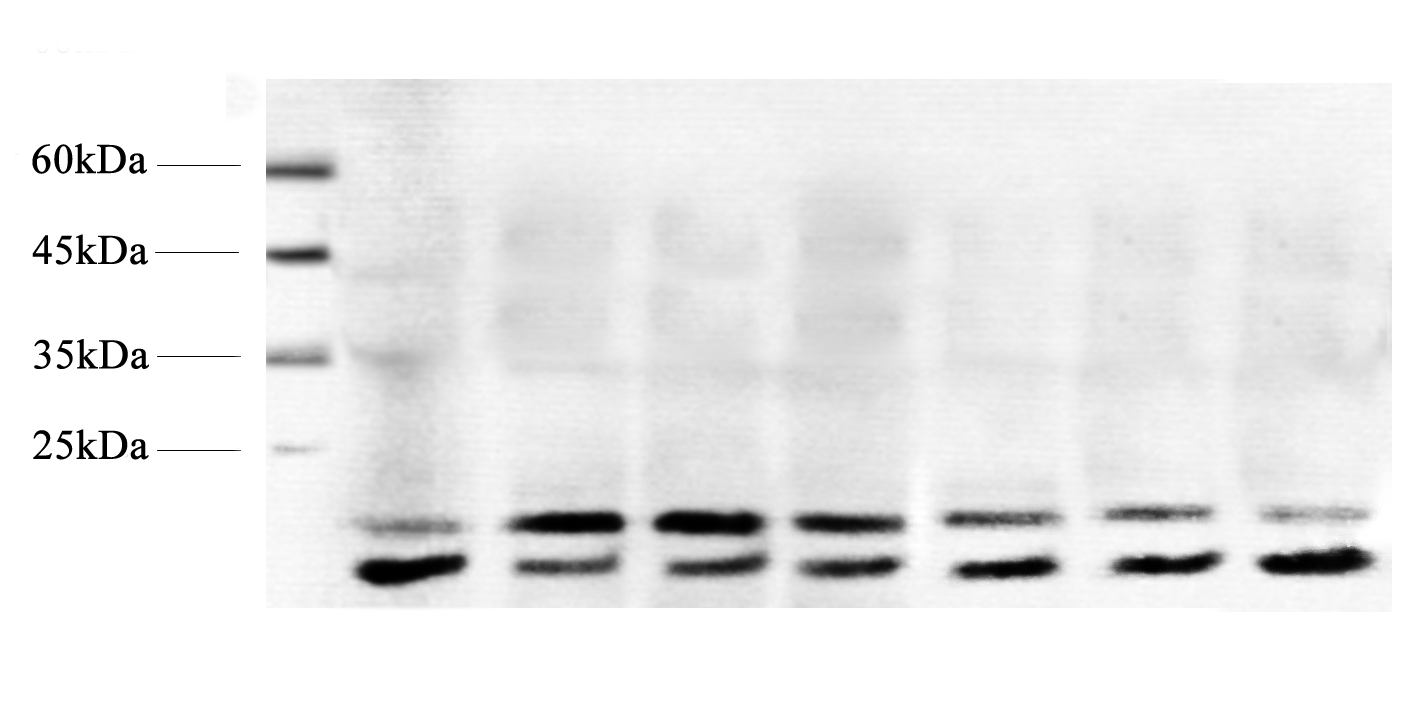

Supplement: Supplementary file 1 [file DataSheet3.ZIP › original data/Fig.3B/LC3-3.tif]

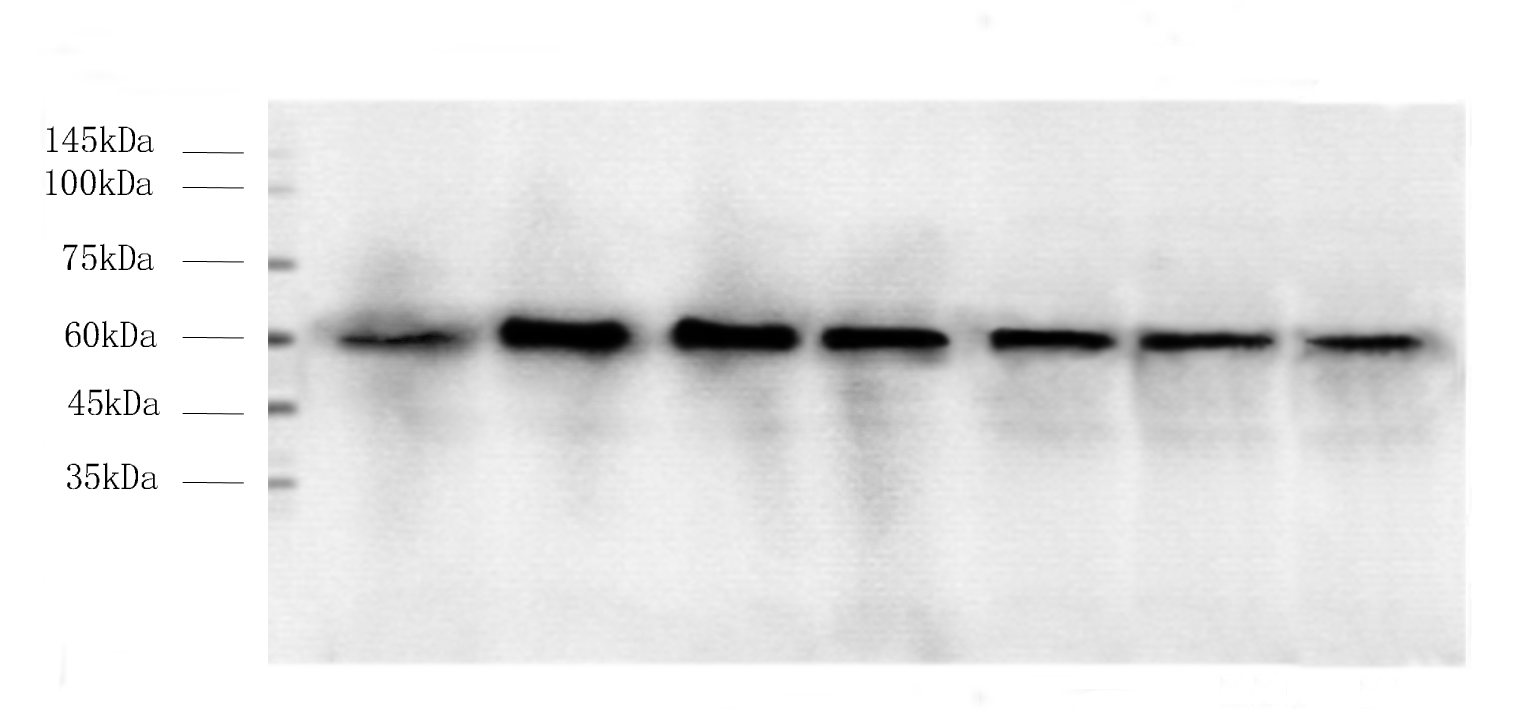

Supplement: Supplementary file 1 [file DataSheet3.ZIP › original data/Fig.3B/P62-1.tif]

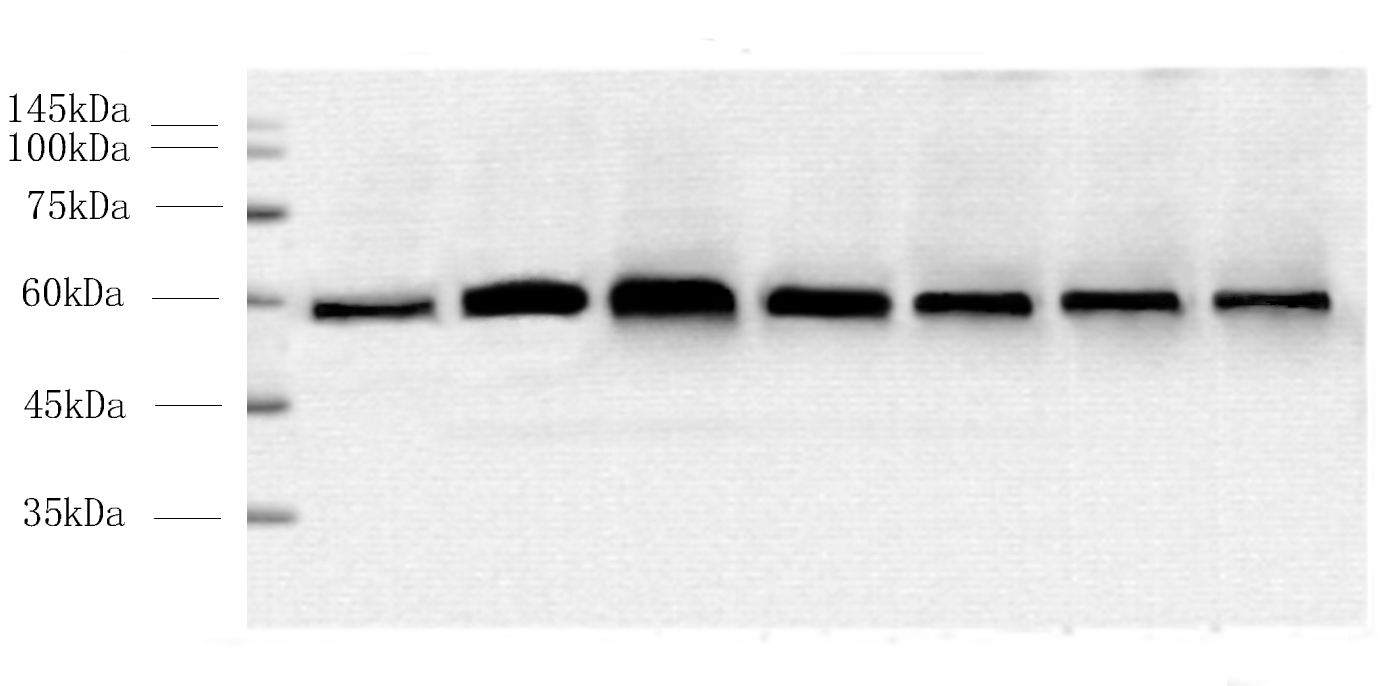

Supplement: Supplementary file 1 [file DataSheet3.ZIP › original data/Fig.3B/P62-2.tif]

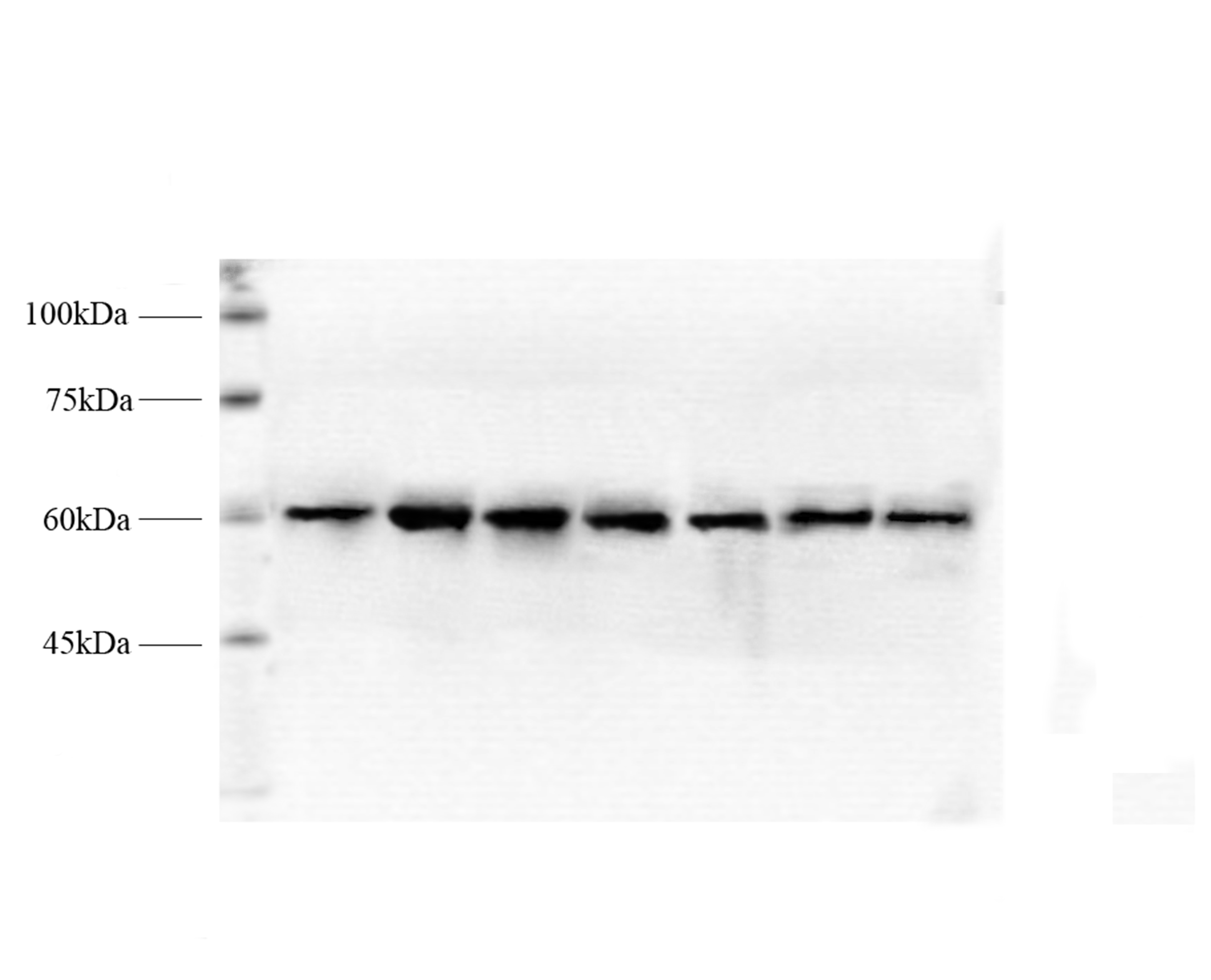

Supplement: Supplementary file 1 [file DataSheet3.ZIP › original data/Fig.3B/P62-3.tif]

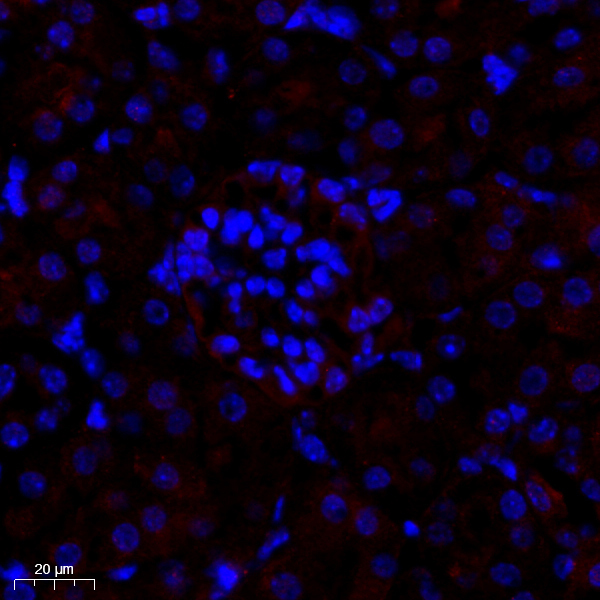

Supplement: Supplementary file 1 [file DataSheet3.ZIP › original data/Fig.4D/1/1-1-1.jpg]

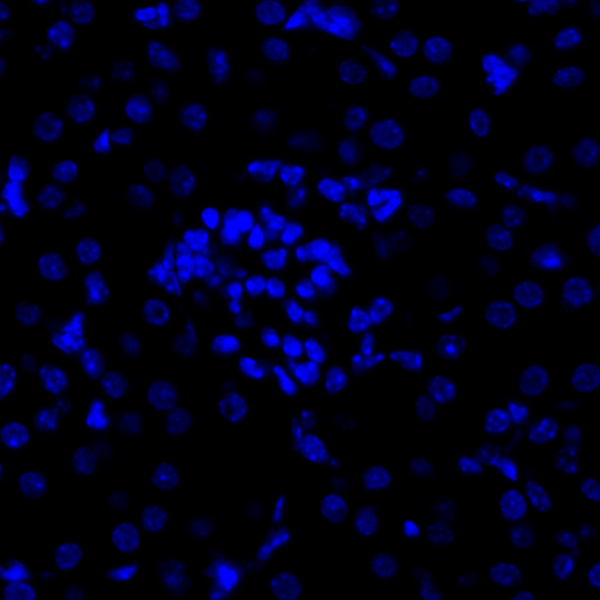

Supplement: Supplementary file 1 [file DataSheet3.ZIP › original data/Fig.4D/1/1-1.jpg]

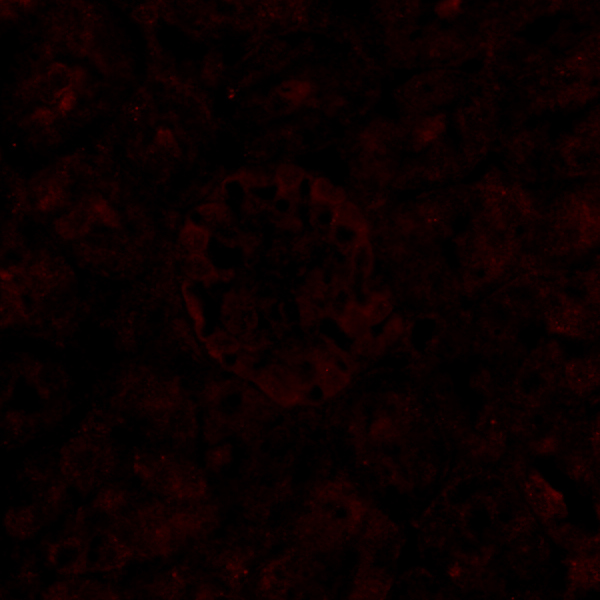

Supplement: Supplementary file 1 [file DataSheet3.ZIP › original data/Fig.4D/1/1.jpg]

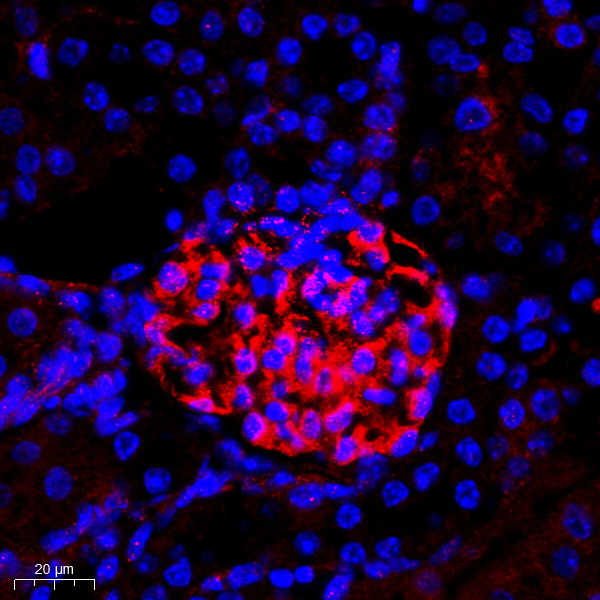

Supplement: Supplementary file 1 [file DataSheet3.ZIP › original data/Fig.4D/2/2-1-1.jpg]

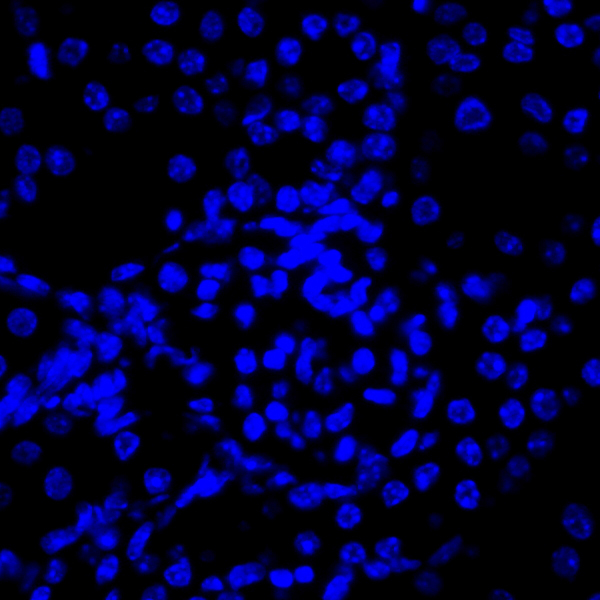

Supplement: Supplementary file 1 [file DataSheet3.ZIP › original data/Fig.4D/2/2-1.jpg]

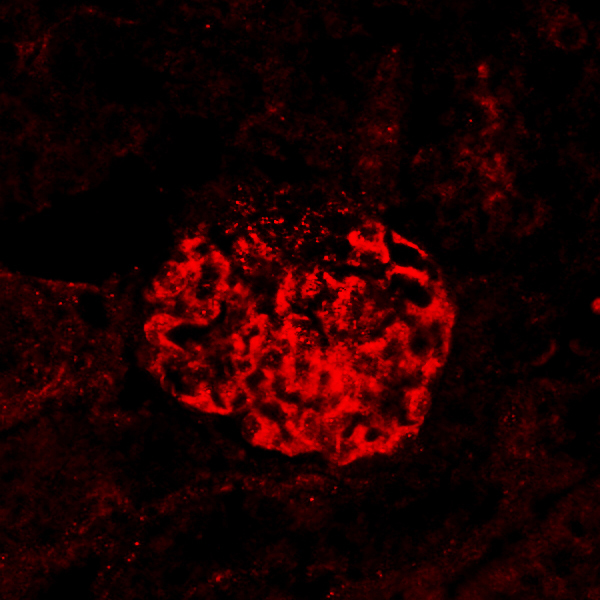

Supplement: Supplementary file 1 [file DataSheet3.ZIP › original data/Fig.4D/2/2.jpg]

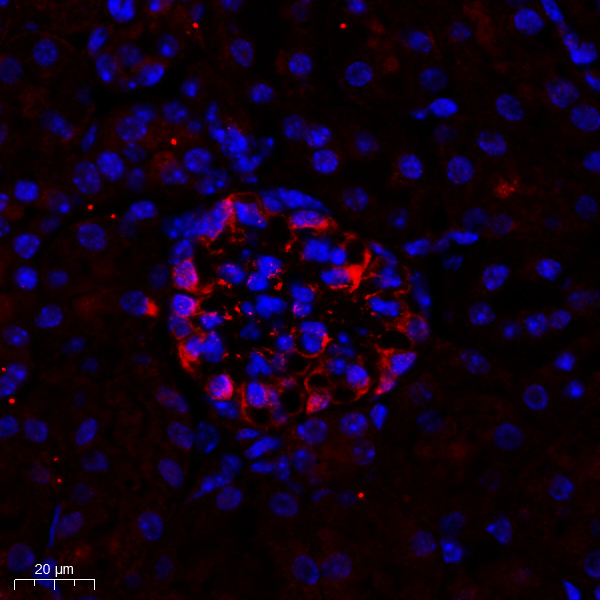

Supplement: Supplementary file 1 [file DataSheet3.ZIP › original data/Fig.4D/3/3-1-1.jpg]

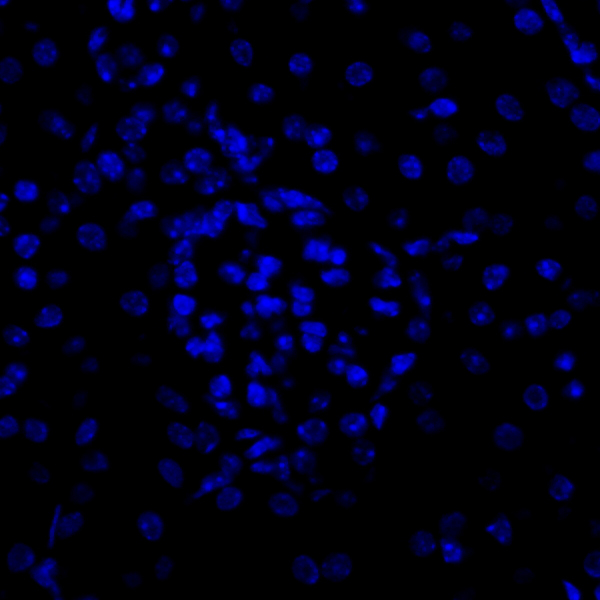

Supplement: Supplementary file 1 [file DataSheet3.ZIP › original data/Fig.4D/3/3-1.jpg]

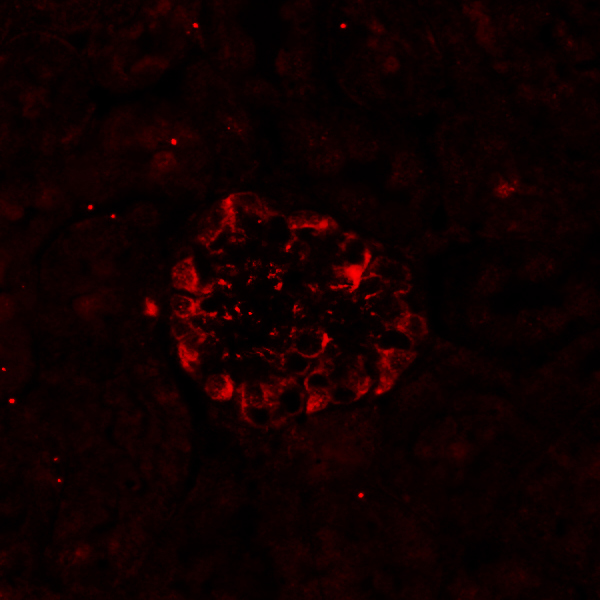

Supplement: Supplementary file 1 [file DataSheet3.ZIP › original data/Fig.4D/3/3.jpg]

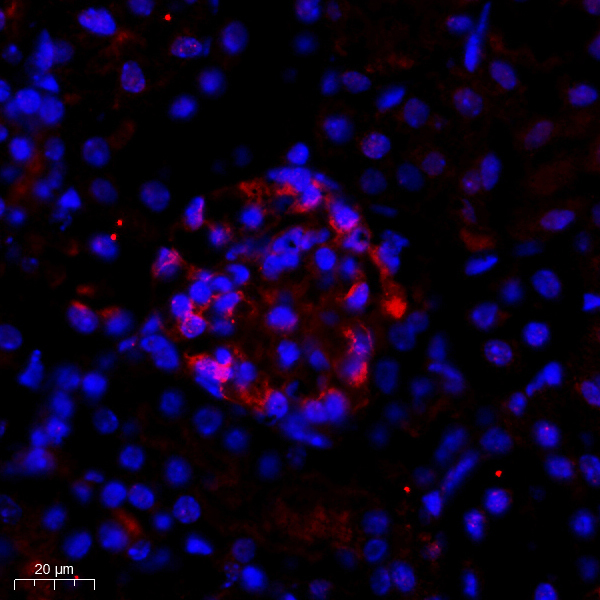

Supplement: Supplementary file 1 [file DataSheet3.ZIP › original data/Fig.4D/4/3-1-1.jpg]

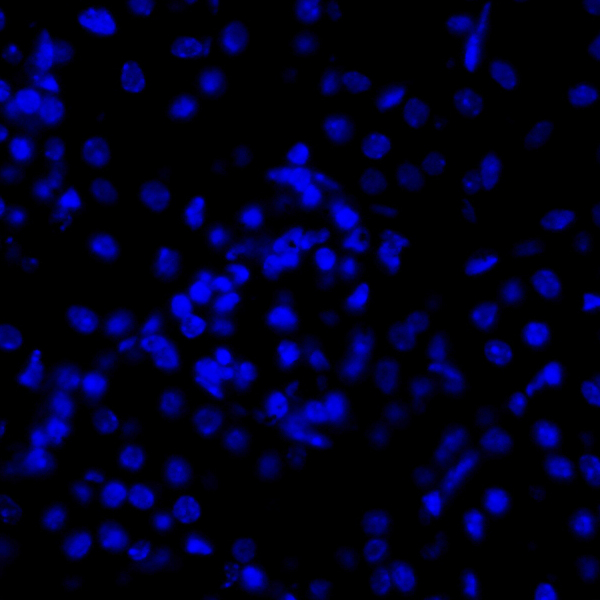

Supplement: Supplementary file 1 [file DataSheet3.ZIP › original data/Fig.4D/4/3-1.jpg]

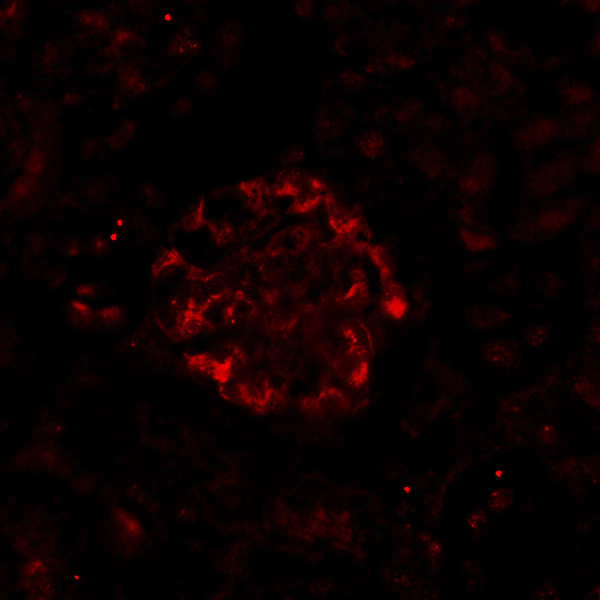

Supplement: Supplementary file 1 [file DataSheet3.ZIP › original data/Fig.4D/4/3.jpg]

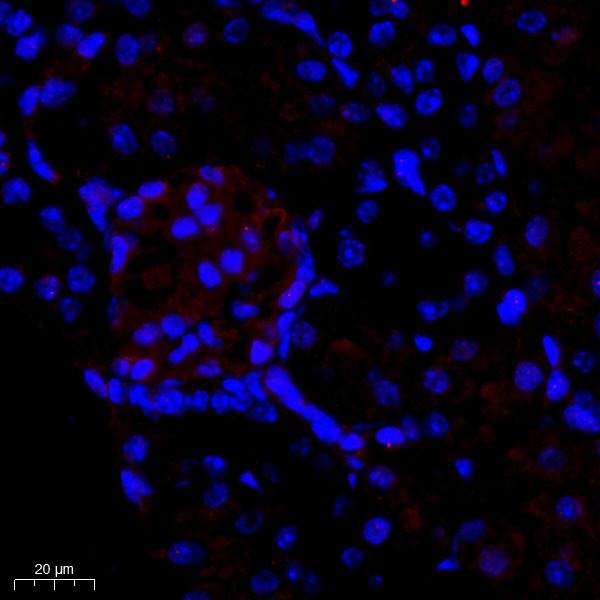

Supplement: Supplementary file 1 [file DataSheet3.ZIP › original data/Fig.4D/5/3-1-1.jpg]

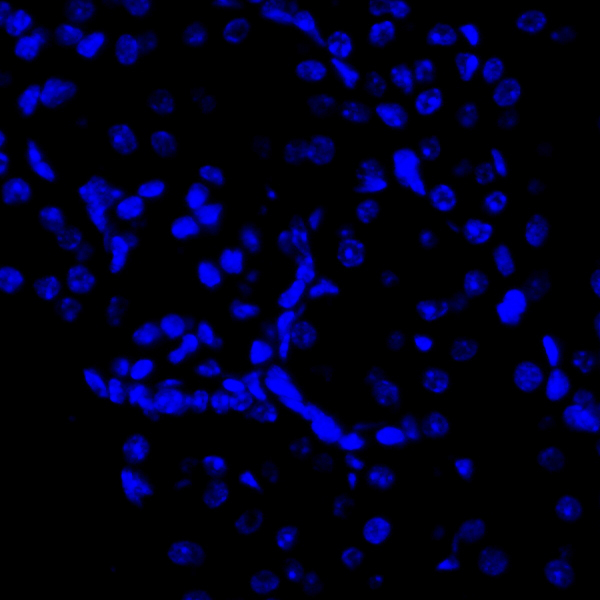

Supplement: Supplementary file 1 [file DataSheet3.ZIP › original data/Fig.4D/5/3-1.jpg]

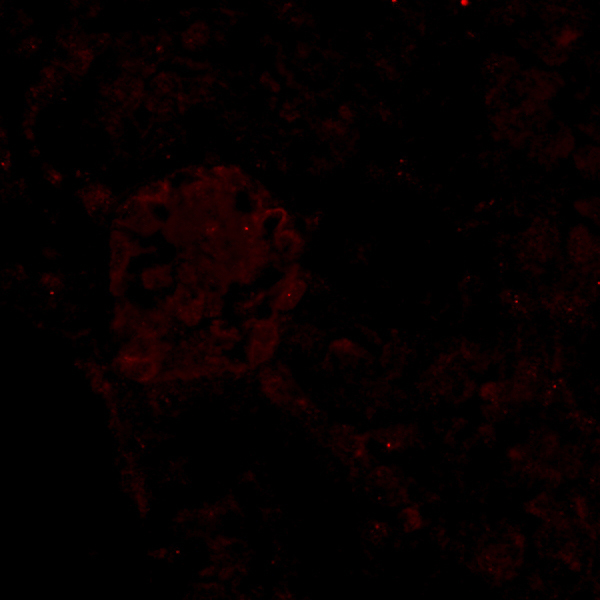

Supplement: Supplementary file 1 [file DataSheet3.ZIP › original data/Fig.4D/5/3.jpg]

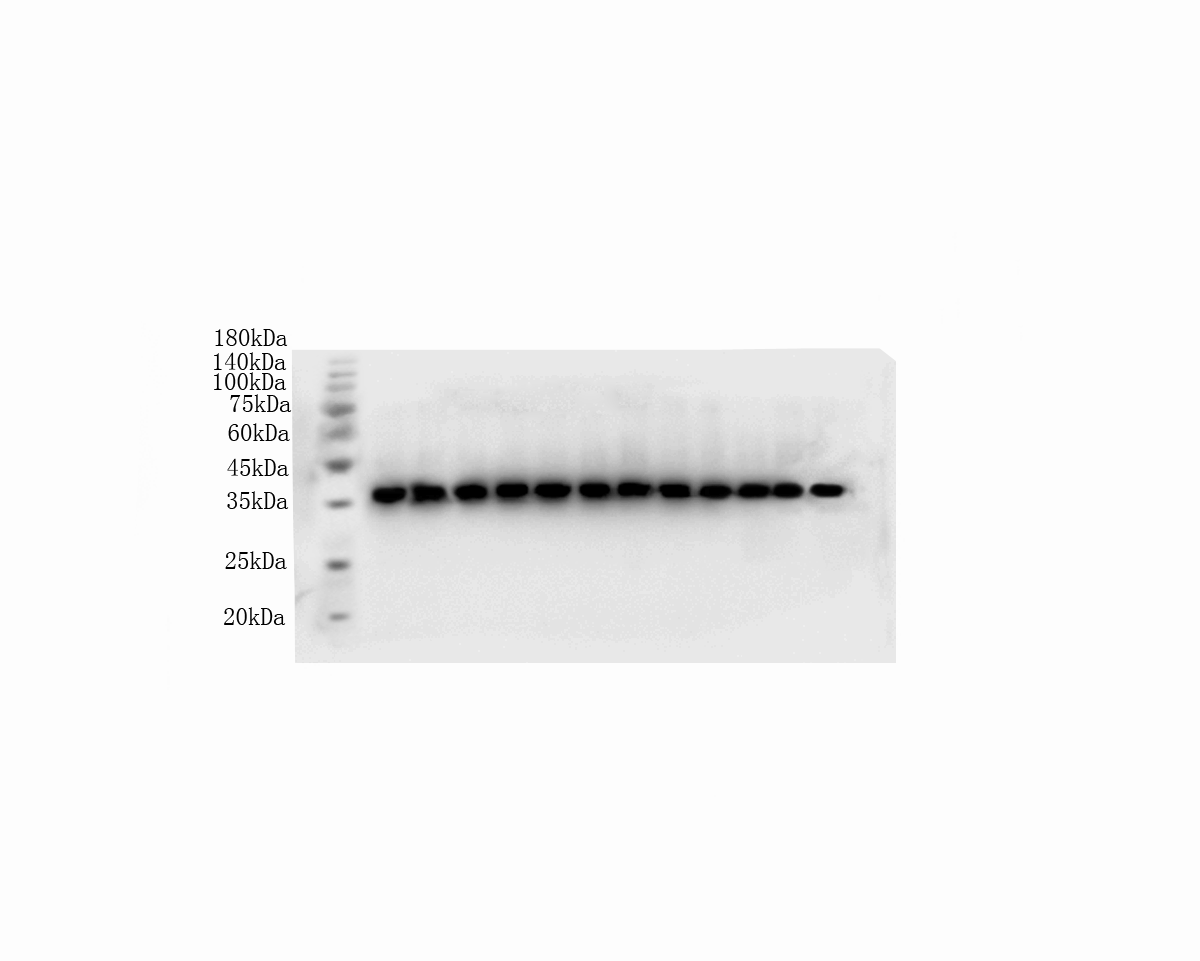

Supplement: Supplementary file 1 [file DataSheet3.ZIP › original data/Fig.5A/GAPDH.tif]

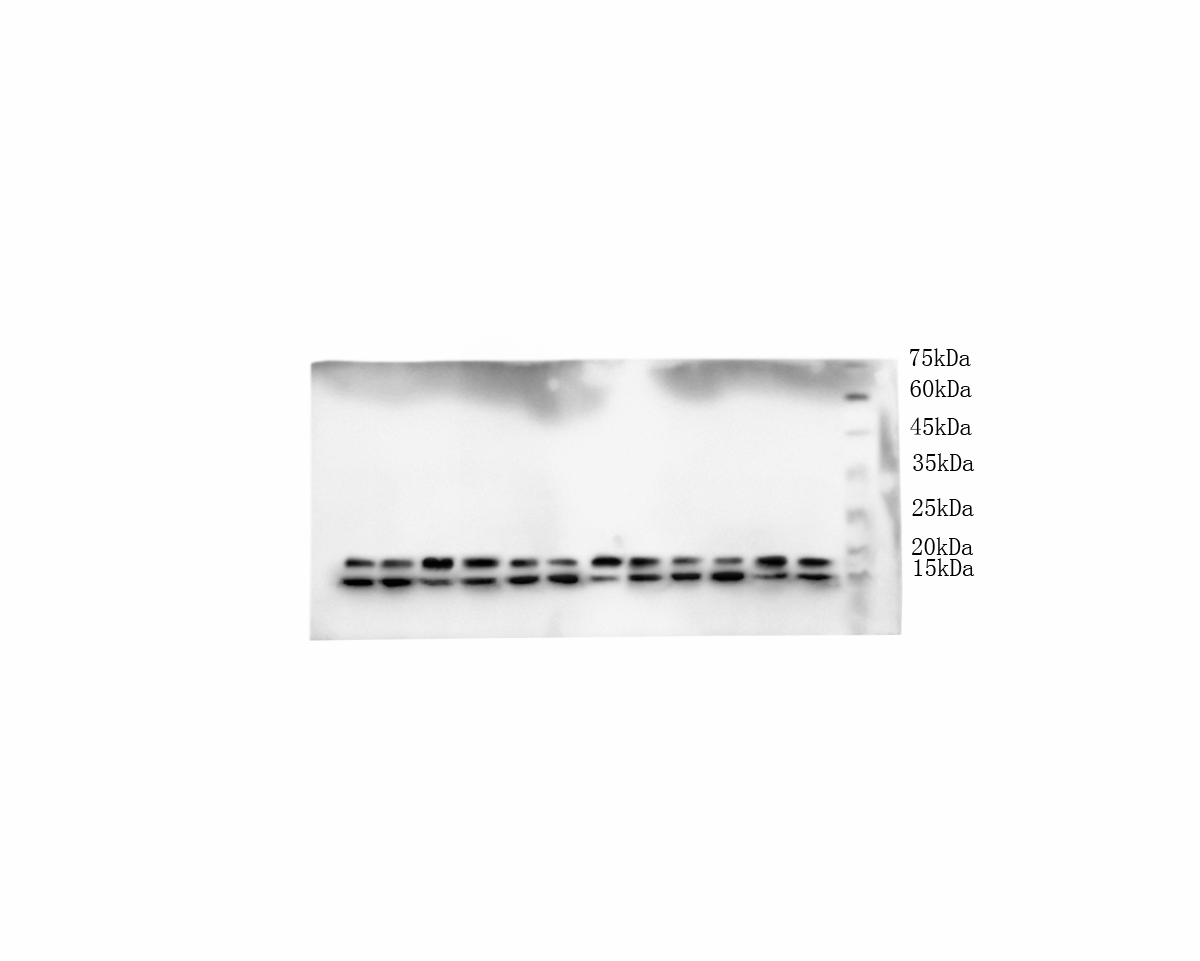

Supplement: Supplementary file 1 [file DataSheet3.ZIP › original data/Fig.5A/lc3.tif]

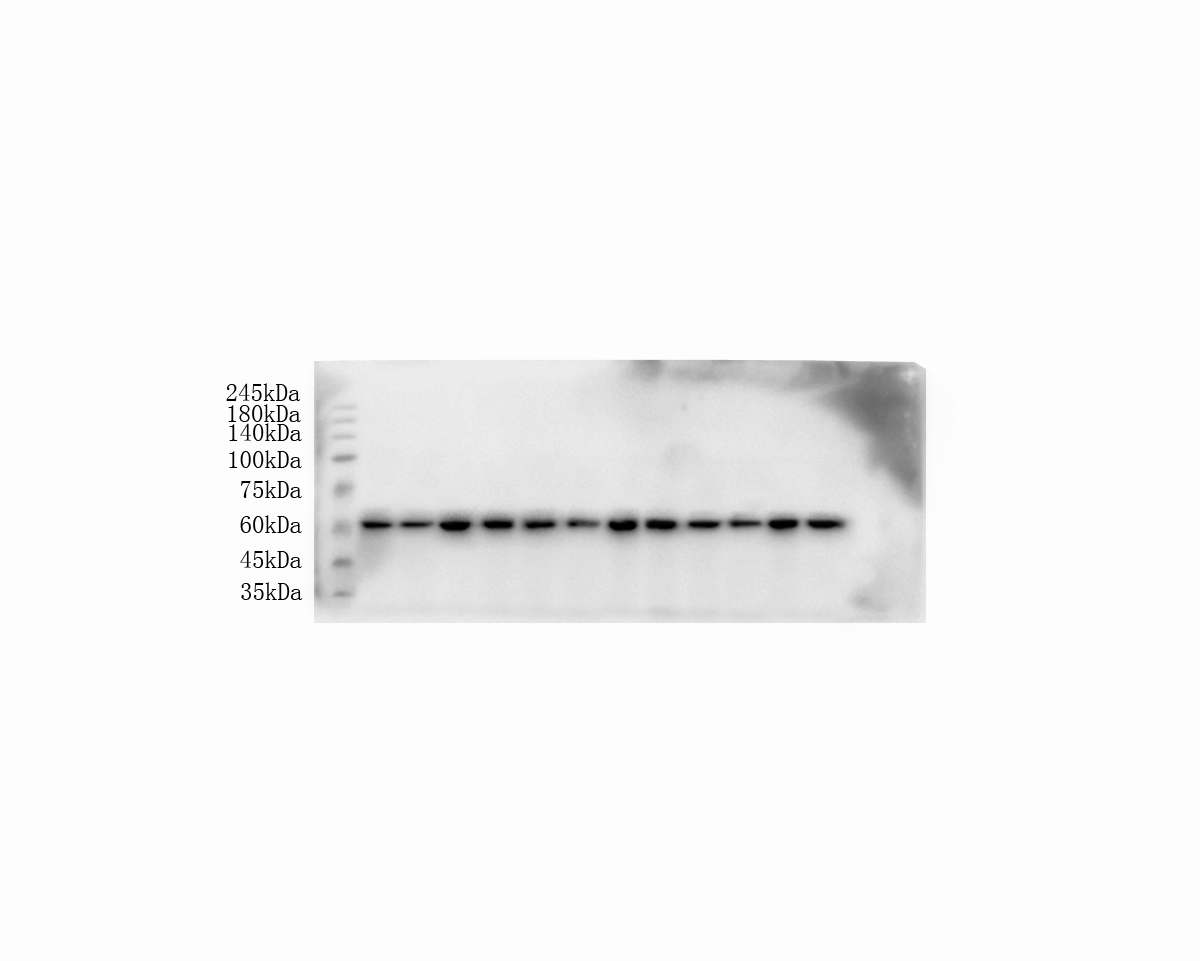

Supplement: Supplementary file 1 [file DataSheet3.ZIP › original data/Fig.5A/P62.tif]

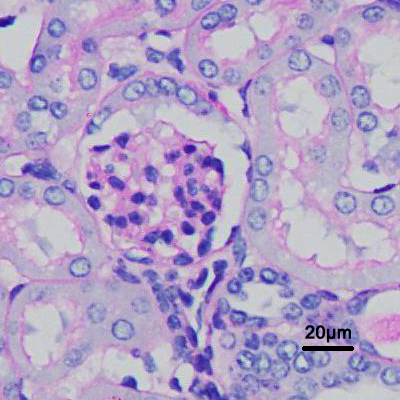

Supplement: Supplementary file 1 [file DataSheet3.ZIP › original data/Fig.6A/1.tif]

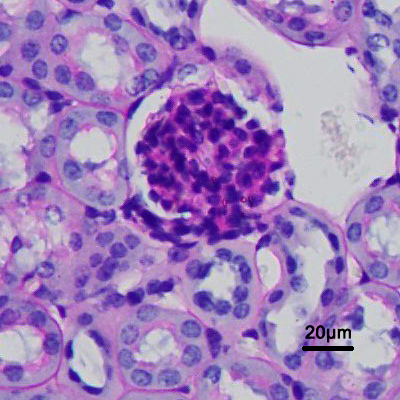

Supplement: Supplementary file 1 [file DataSheet3.ZIP › original data/Fig.6A/2.tif]

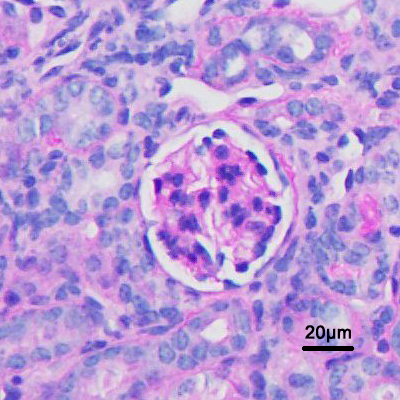

Supplement: Supplementary file 1 [file DataSheet3.ZIP › original data/Fig.6A/3.tif]

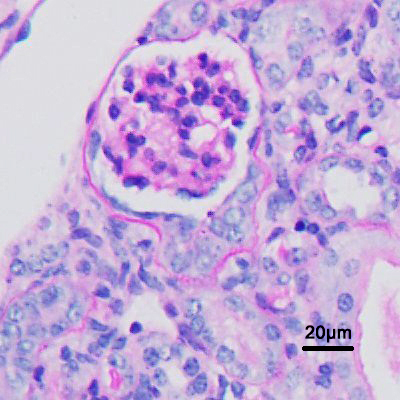

Supplement: Supplementary file 1 [file DataSheet3.ZIP › original data/Fig.6A/4.tif]

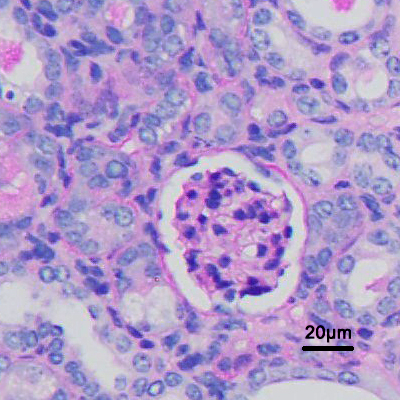

Supplement: Supplementary file 1 [file DataSheet3.ZIP › original data/Fig.6A/5.tif]

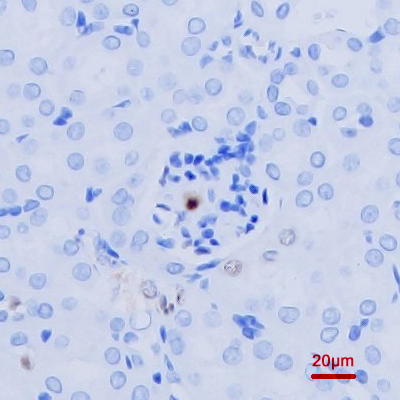

Supplement: Supplementary file 1 [file DataSheet3.ZIP › original data/Fig.6B/1-1.jpg]

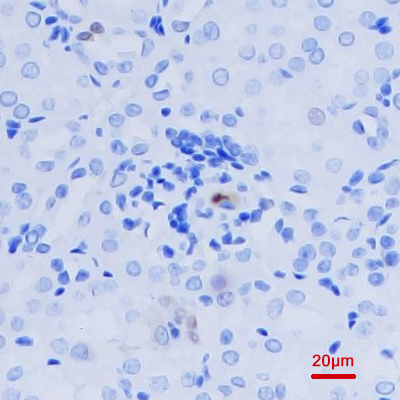

Supplement: Supplementary file 1 [file DataSheet3.ZIP › original data/Fig.6B/1-2.jpg]

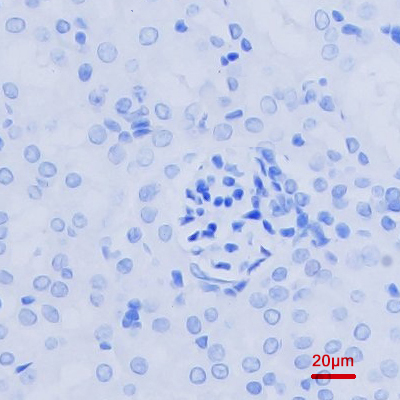

Supplement: Supplementary file 1 [file DataSheet3.ZIP › original data/Fig.6B/1-3.jpg]

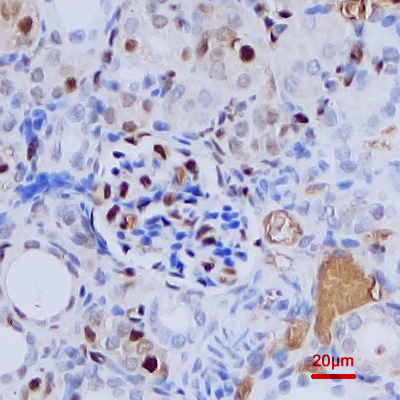

Supplement: Supplementary file 1 [file DataSheet3.ZIP › original data/Fig.6B/2-1.tif]

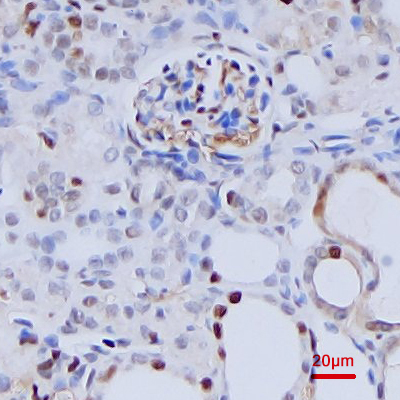

Supplement: Supplementary file 1 [file DataSheet3.ZIP › original data/Fig.6B/2-2.jpg]

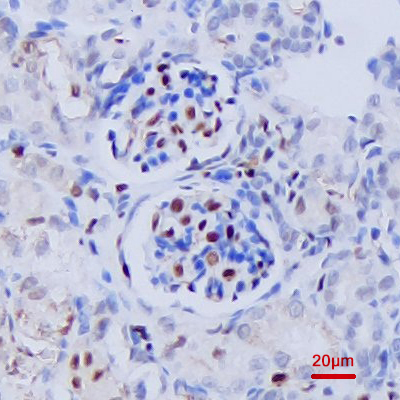

Supplement: Supplementary file 1 [file DataSheet3.ZIP › original data/Fig.6B/2-3.jpg]

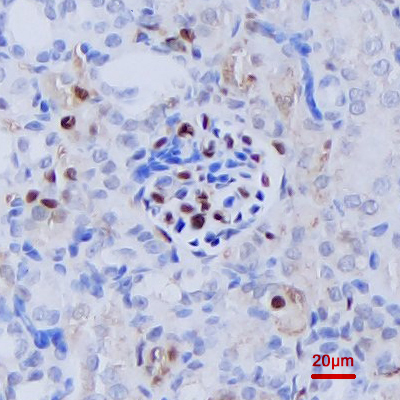

Supplement: Supplementary file 1 [file DataSheet3.ZIP › original data/Fig.6B/3-1.jpg]

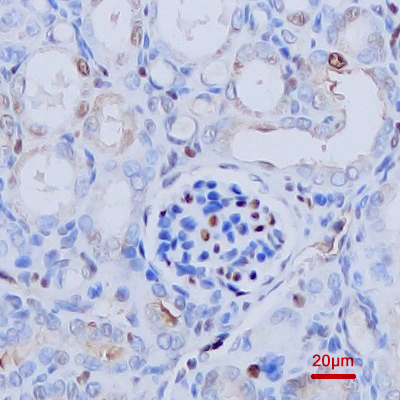

Supplement: Supplementary file 1 [file DataSheet3.ZIP › original data/Fig.6B/3-2.jpg]

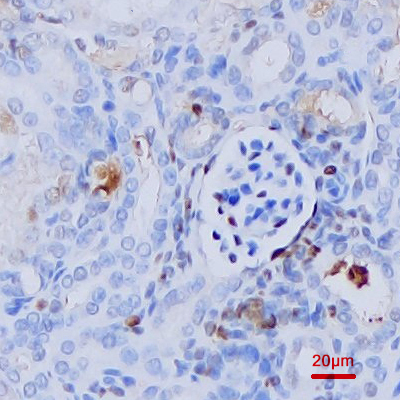

Supplement: Supplementary file 1 [file DataSheet3.ZIP › original data/Fig.6B/3-3.jpg]

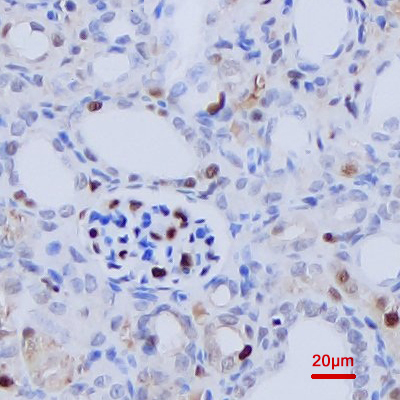

Supplement: Supplementary file 1 [file DataSheet3.ZIP › original data/Fig.6B/4-1.jpg]

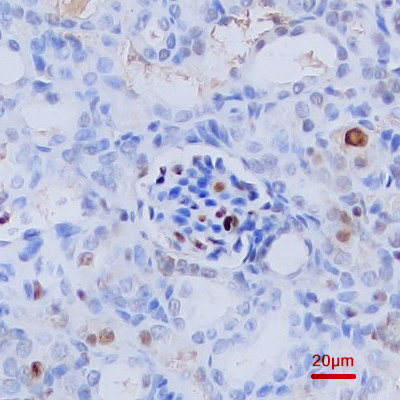

Supplement: Supplementary file 1 [file DataSheet3.ZIP › original data/Fig.6B/4-2.jpg]

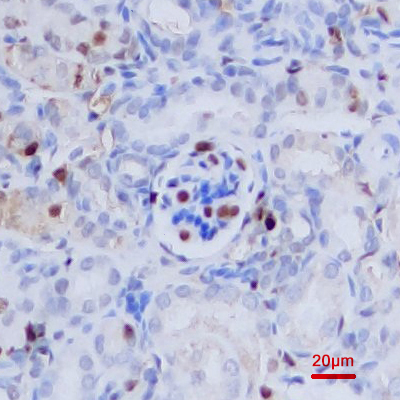

Supplement: Supplementary file 1 [file DataSheet3.ZIP › original data/Fig.6B/4-3.jpg]

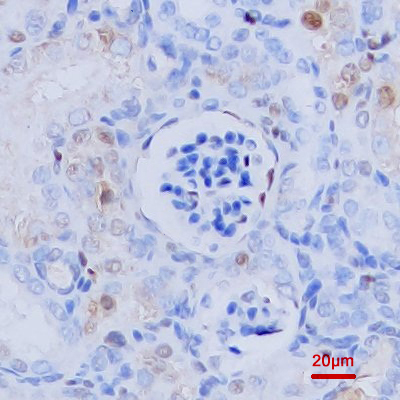

Supplement: Supplementary file 1 [file DataSheet3.ZIP › original data/Fig.6B/5-1.jpg]

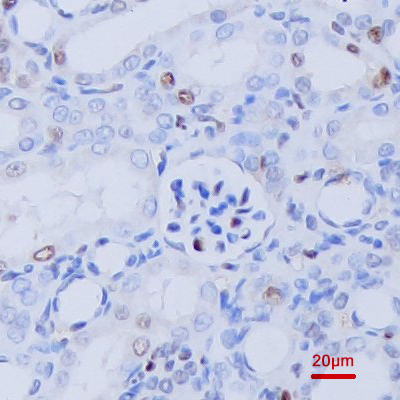

Supplement: Supplementary file 1 [file DataSheet3.ZIP › original data/Fig.6B/5-2.jpg]

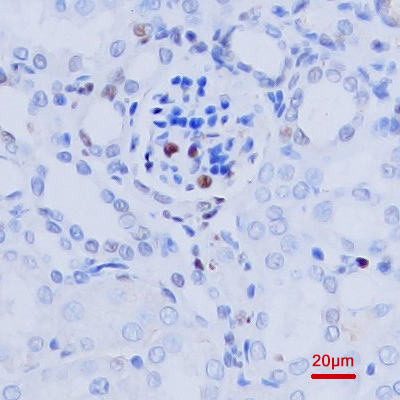

Supplement: Supplementary file 1 [file DataSheet3.ZIP › original data/Fig.6B/5-3.jpg]

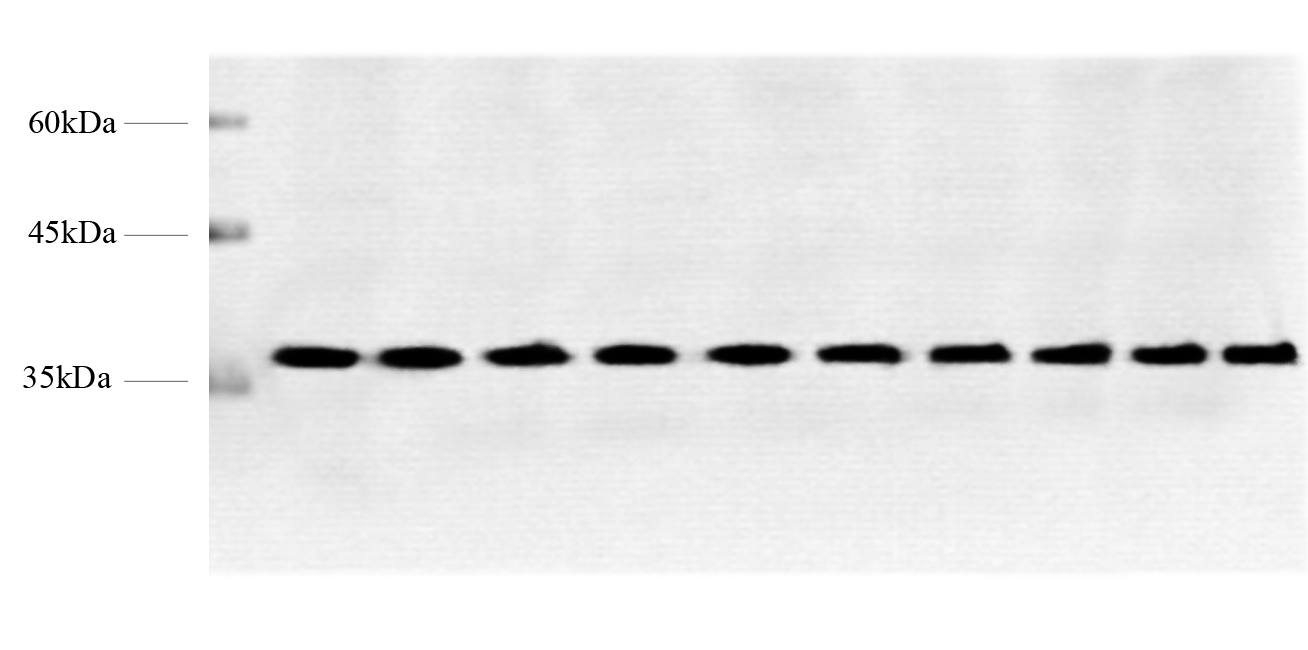

Supplement: Supplementary file 1 [file DataSheet3.ZIP › original data/Fig.6C/GAPDH-1.tif]

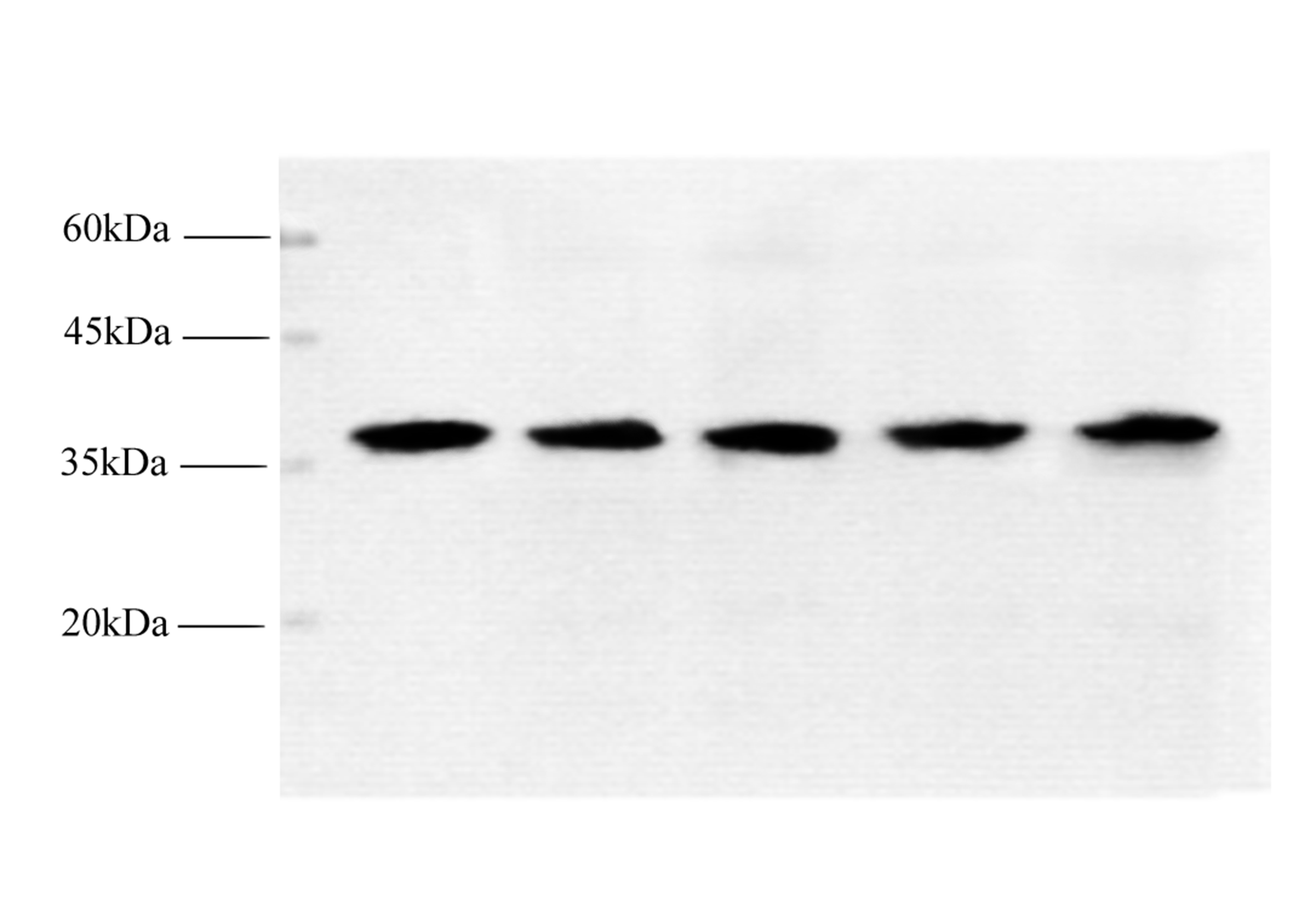

Supplement: Supplementary file 1 [file DataSheet3.ZIP › original data/Fig.6C/GAPDH-2.tif]

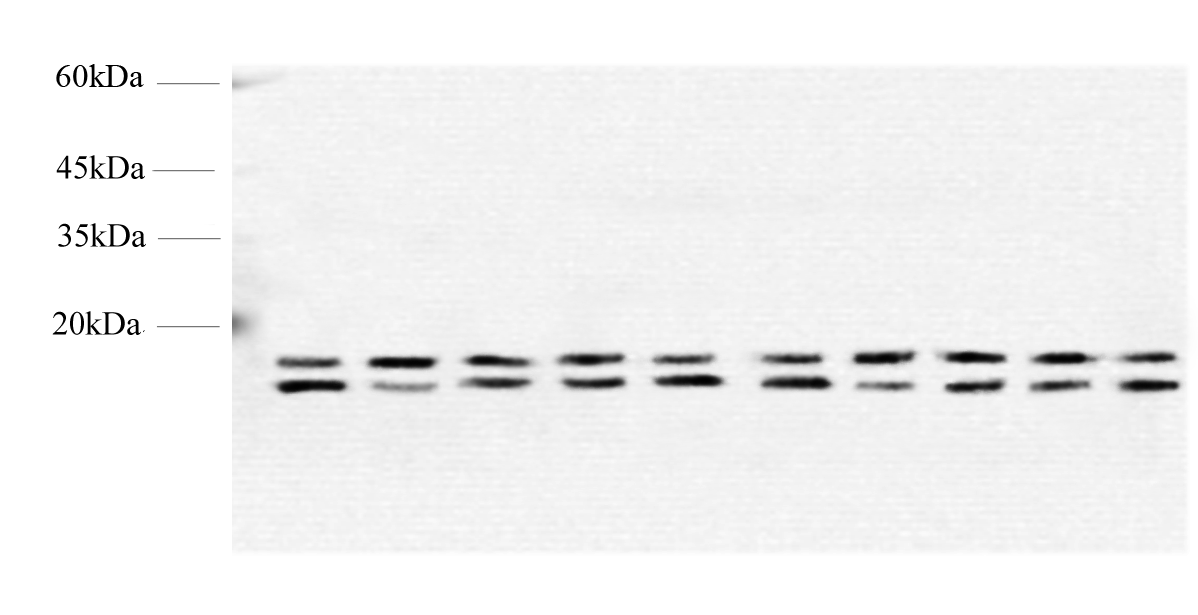

Supplement: Supplementary file 1 [file DataSheet3.ZIP › original data/Fig.6C/LC3-1.tif]

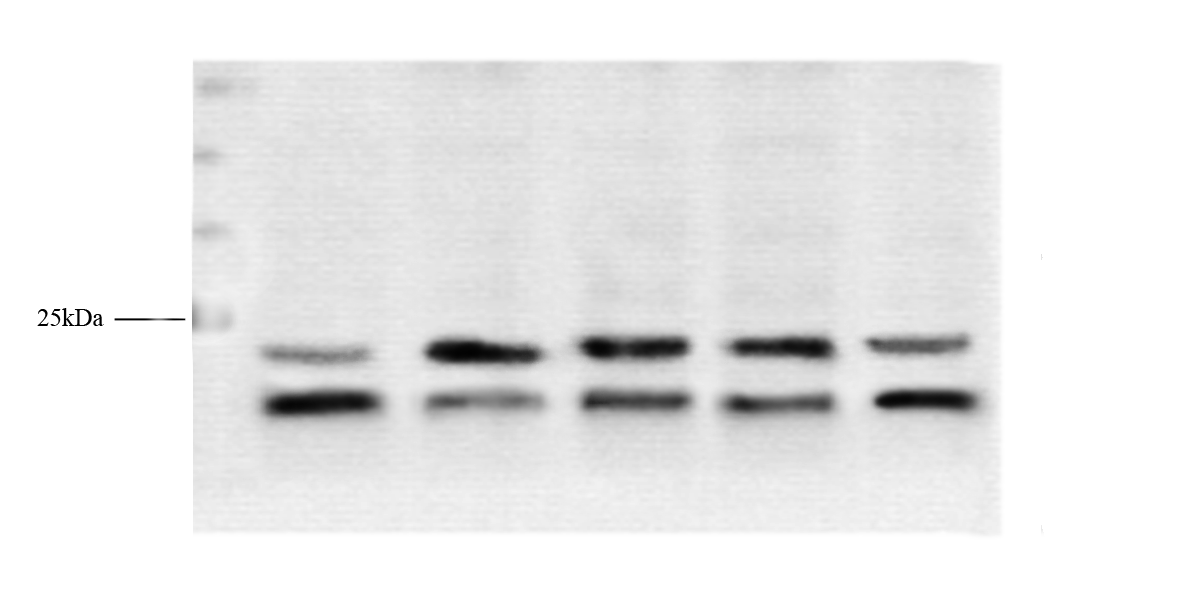

Supplement: Supplementary file 1 [file DataSheet3.ZIP › original data/Fig.6C/LC3-2.tif]

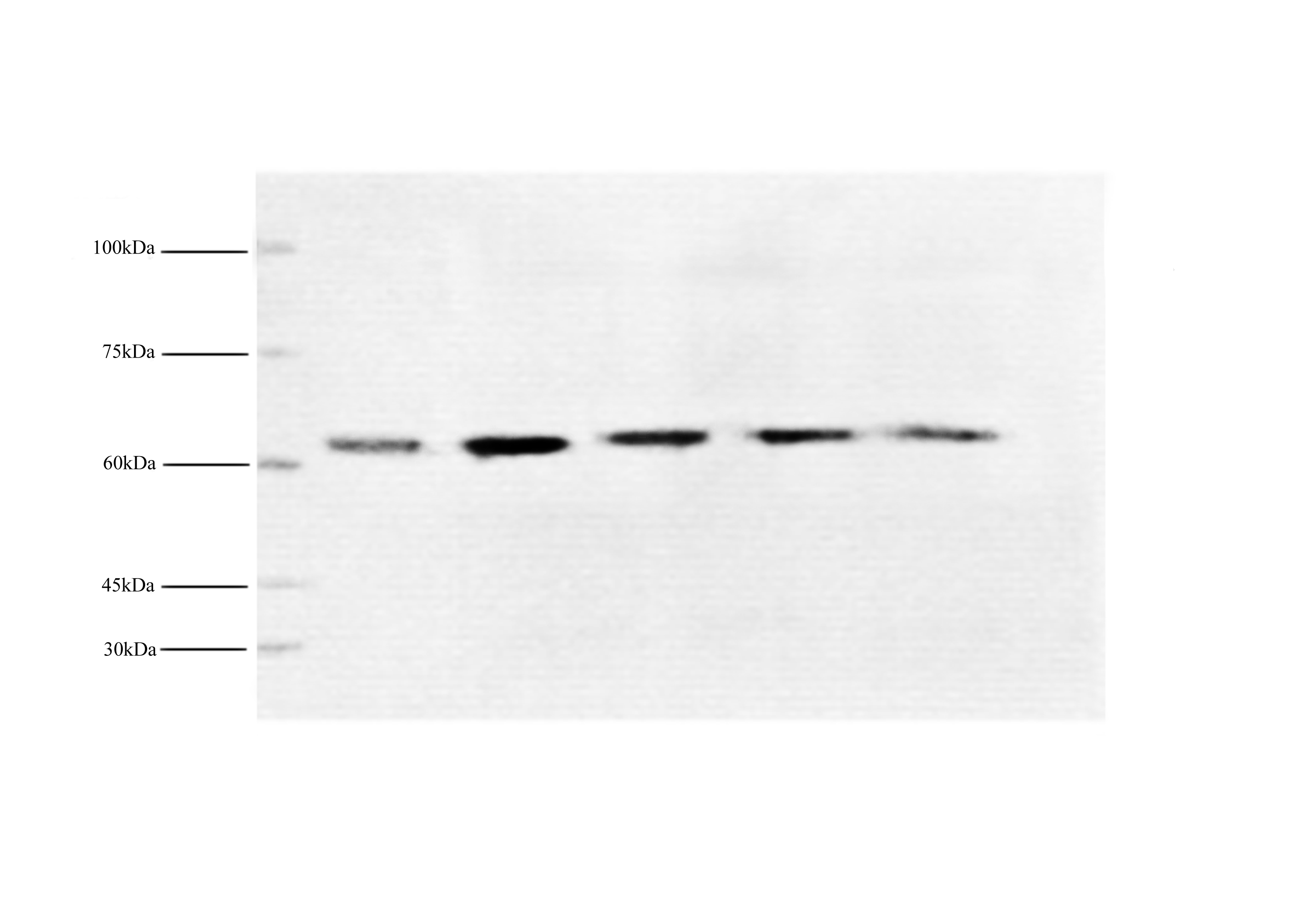

Supplement: Supplementary file 1 [file DataSheet3.ZIP › original data/Fig.6C/P62-2.tif]

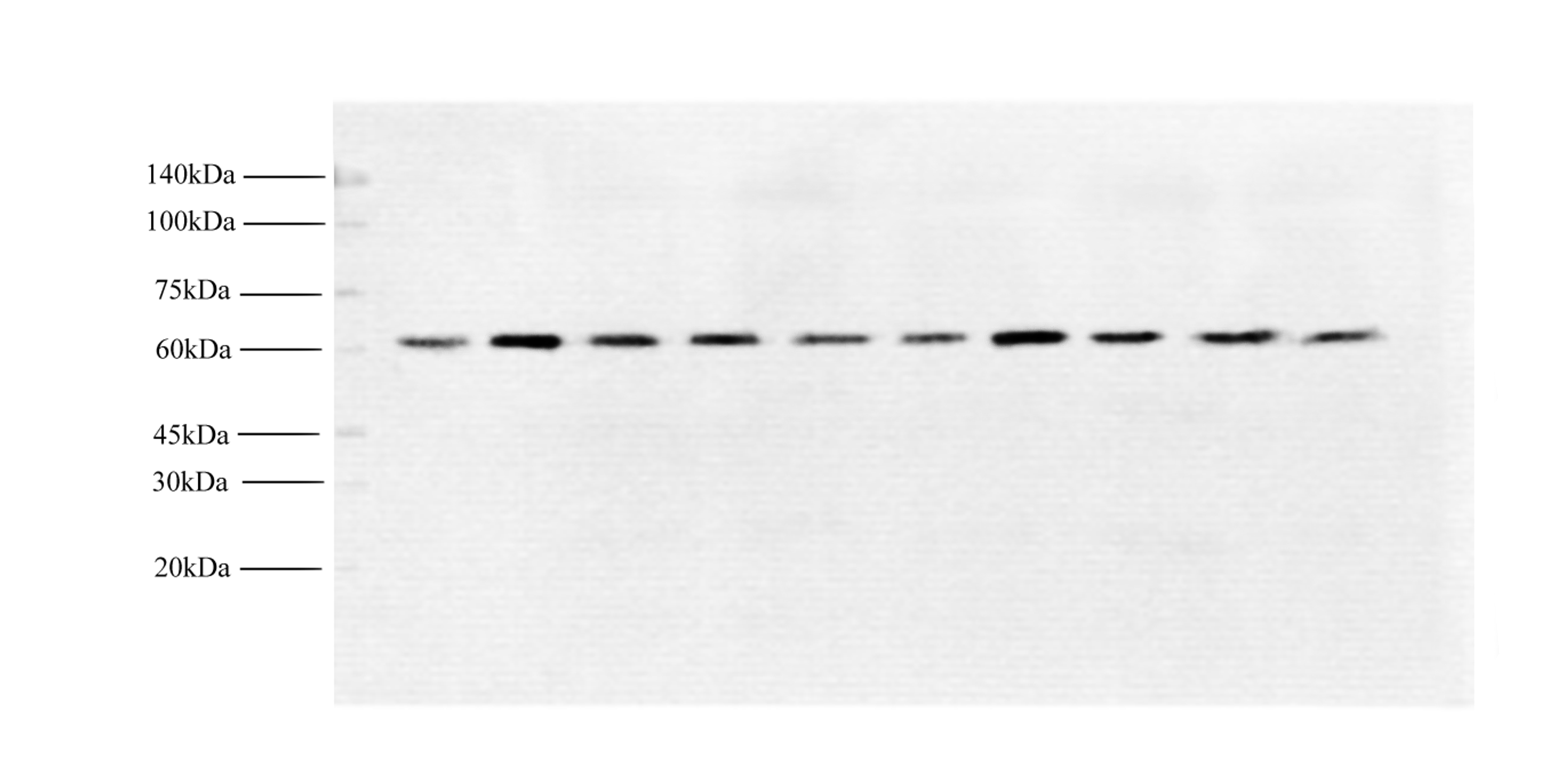

Supplement: Supplementary file 1 [file DataSheet3.ZIP › original data/Fig.6C/p62.tif]

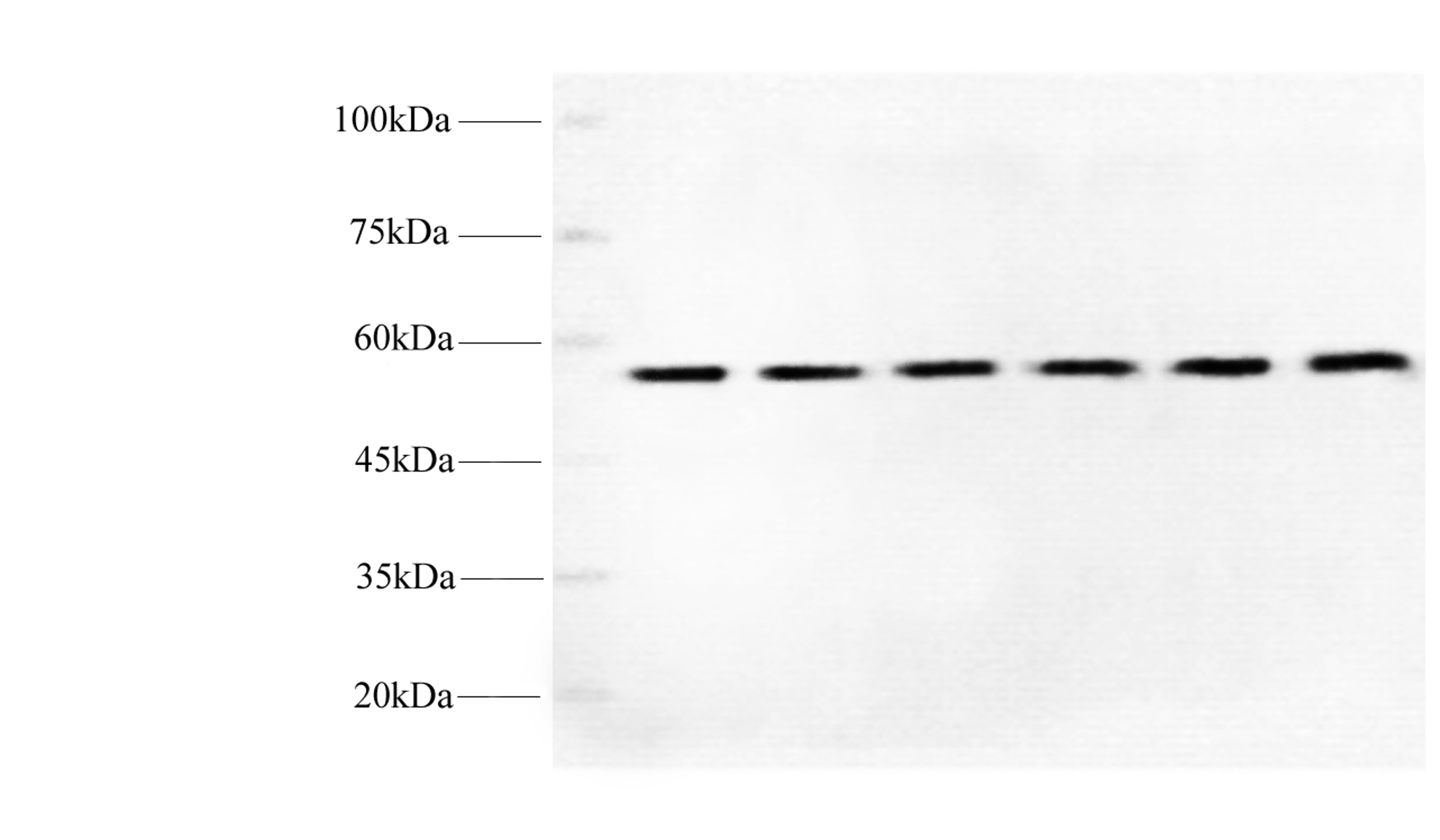

Supplement: Supplementary file 2 [file DataSheet1.ZIP › SUPPLEMENTAL FIGURE/S1A/AKT-1.tif]

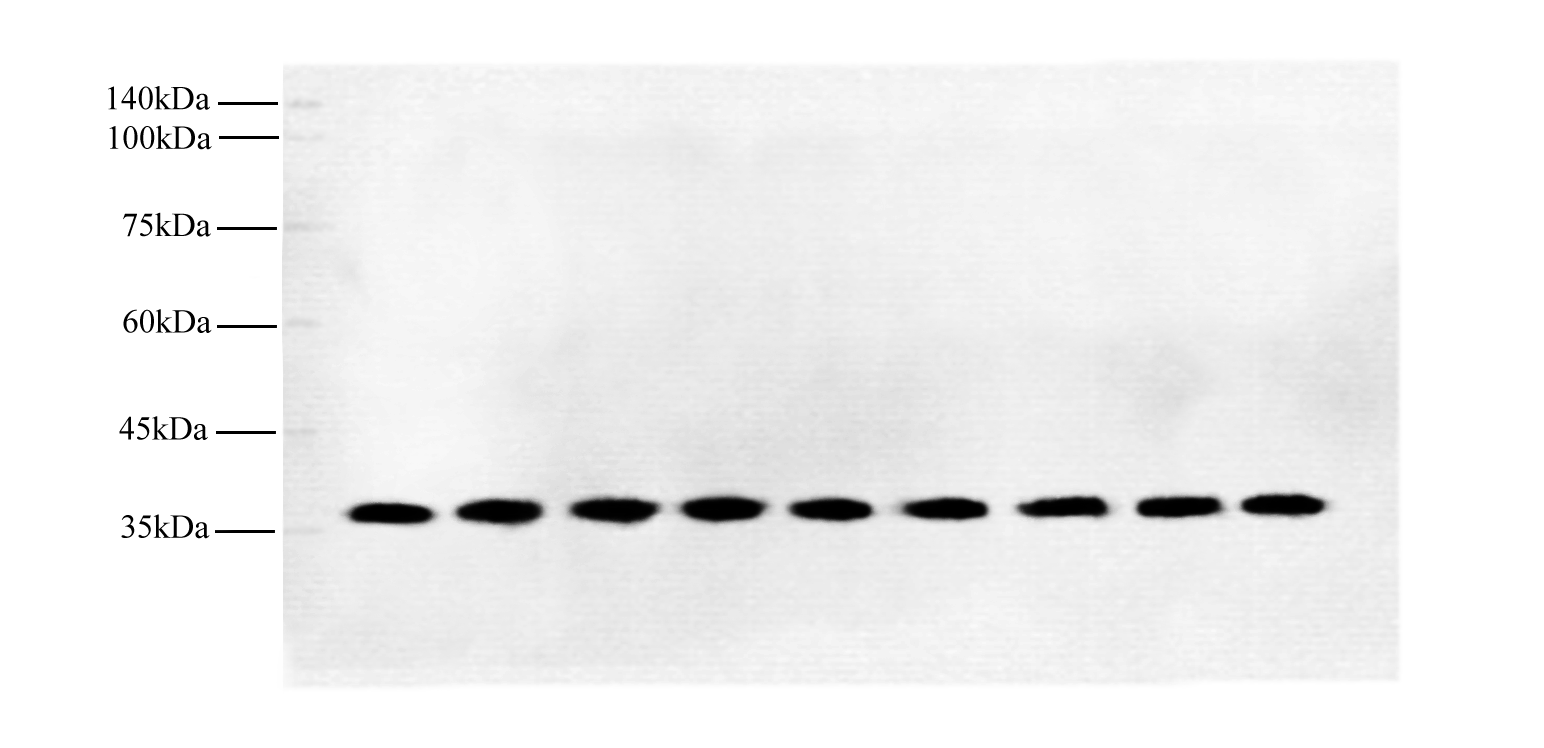

Supplement: Supplementary file 2 [file DataSheet1.ZIP › SUPPLEMENTAL FIGURE/S1A/GAPDH.tif]

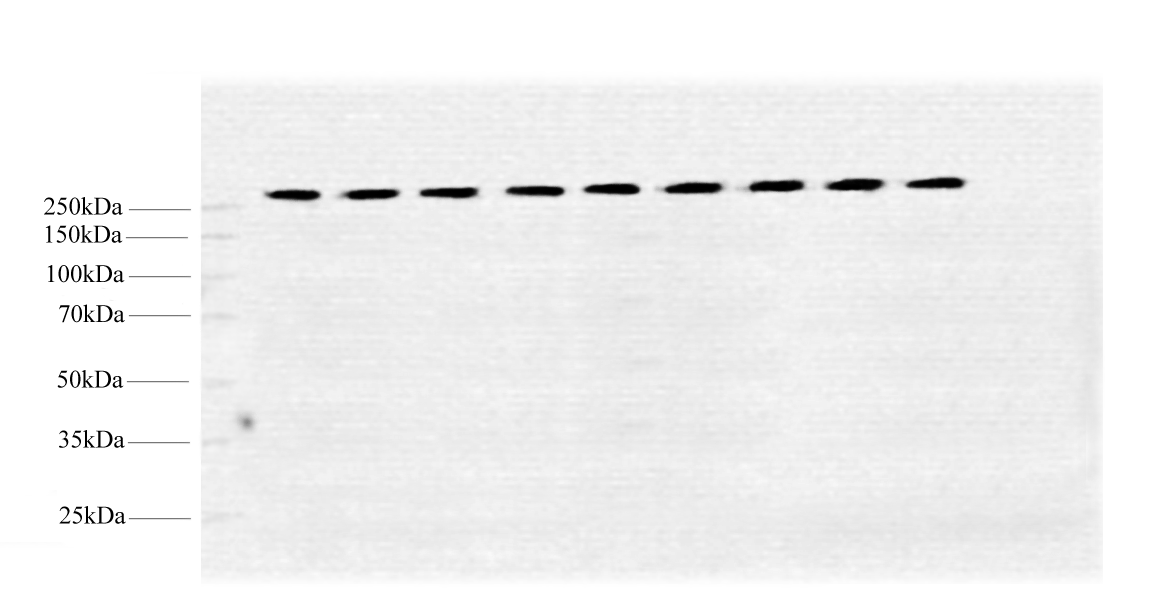

Supplement: Supplementary file 2 [file DataSheet1.ZIP › SUPPLEMENTAL FIGURE/S1A/mTOR.tif]

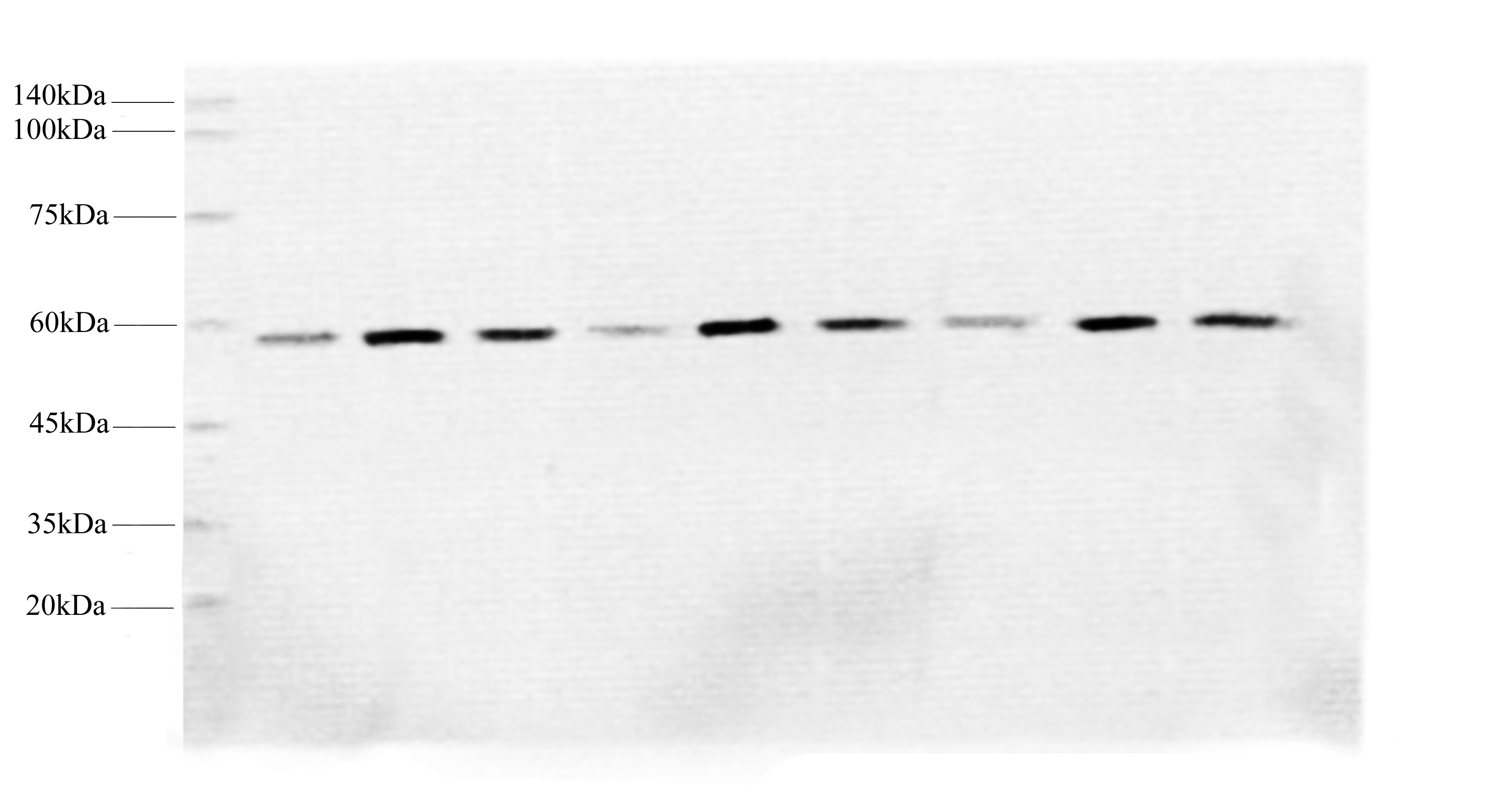

Supplement: Supplementary file 2 [file DataSheet1.ZIP › SUPPLEMENTAL FIGURE/S1A/p-AKT-1.jpg]

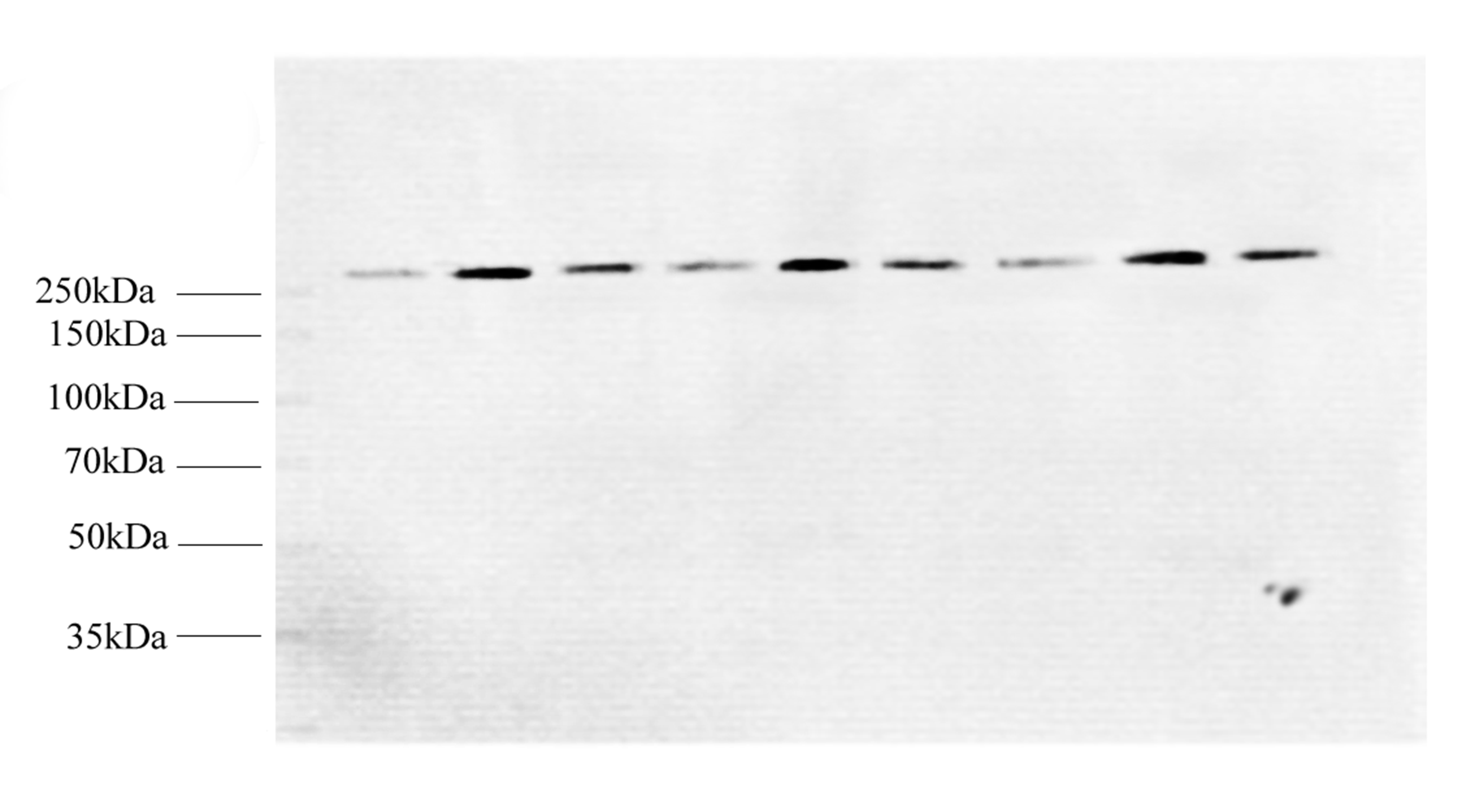

Supplement: Supplementary file 2 [file DataSheet1.ZIP › SUPPLEMENTAL FIGURE/S1A/p-mTOR.tif]

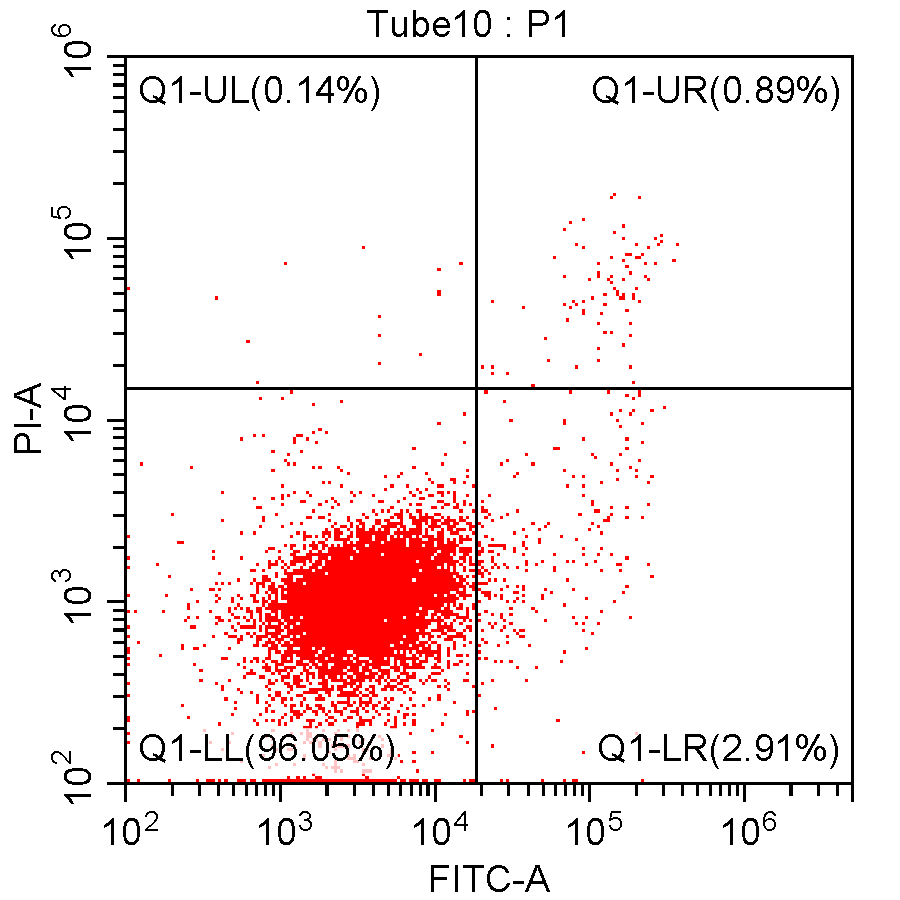

Supplement: Supplementary file 3 [file DataSheet2.ZIP › Flow cytometry/Fig.1C 6 groups/Tube10_Plot1.bmp]

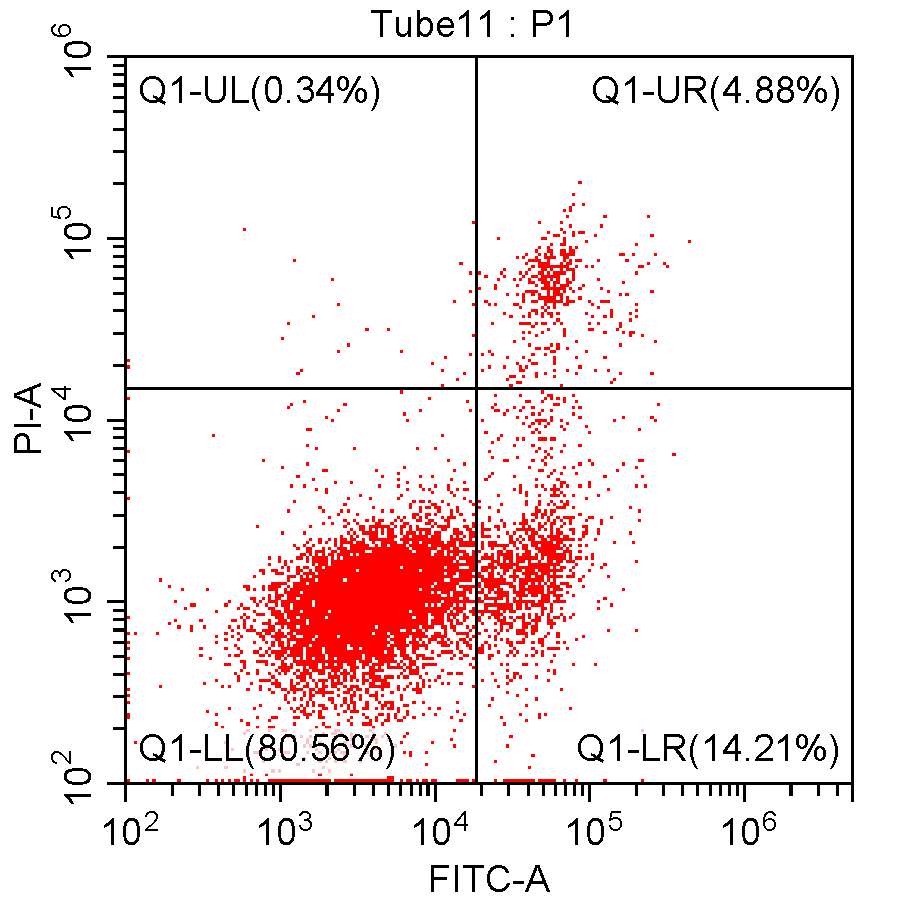

Supplement: Supplementary file 3 [file DataSheet2.ZIP › Flow cytometry/Fig.1C 6 groups/Tube11_Plot1.bmp]

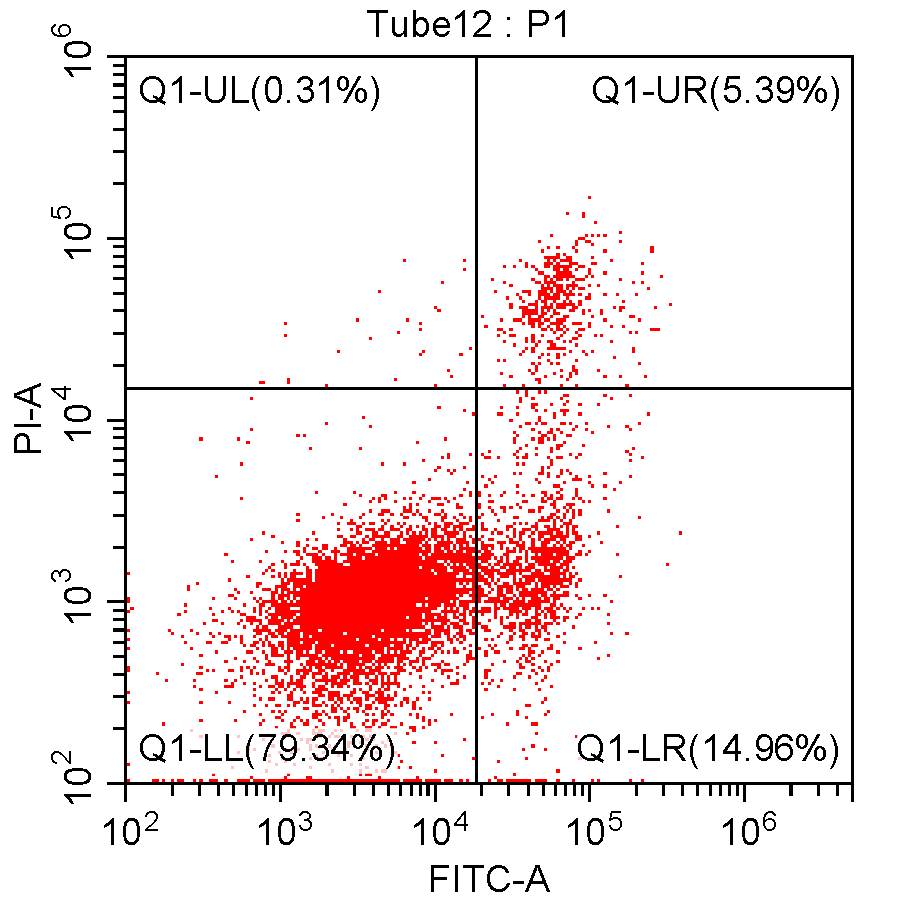

Supplement: Supplementary file 3 [file DataSheet2.ZIP › Flow cytometry/Fig.1C 6 groups/Tube12_Plot1.bmp]

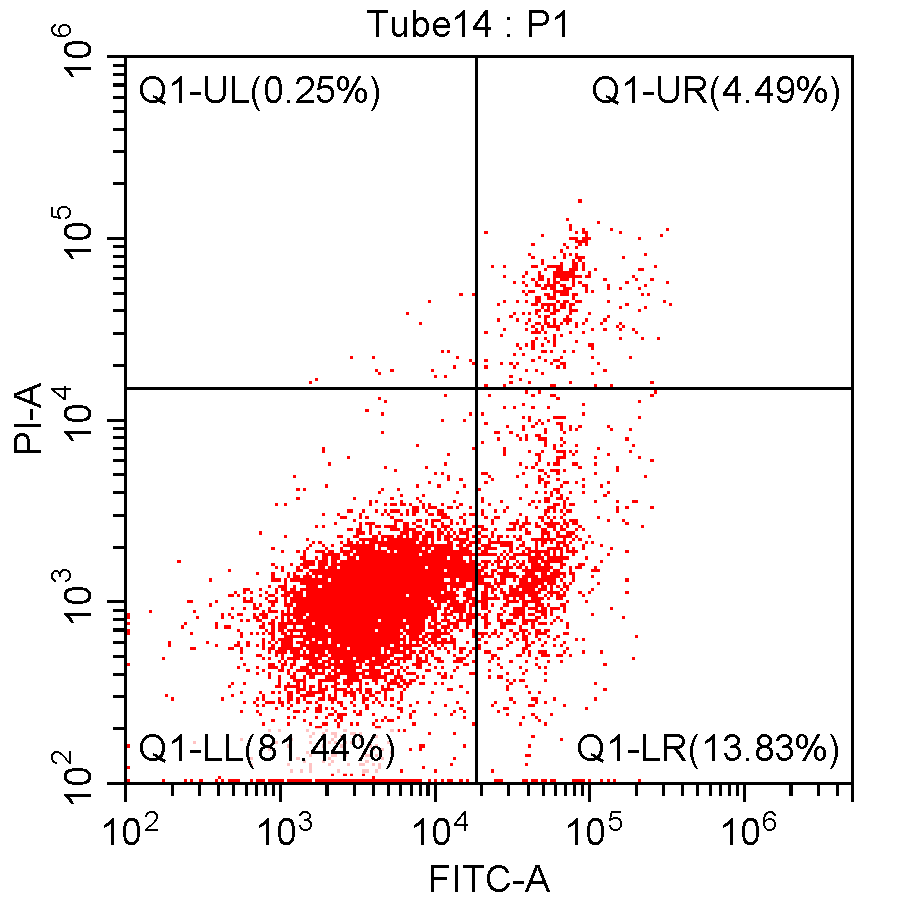

Supplement: Supplementary file 3 [file DataSheet2.ZIP › Flow cytometry/Fig.1C 6 groups/Tube13_Plot1.bmp]

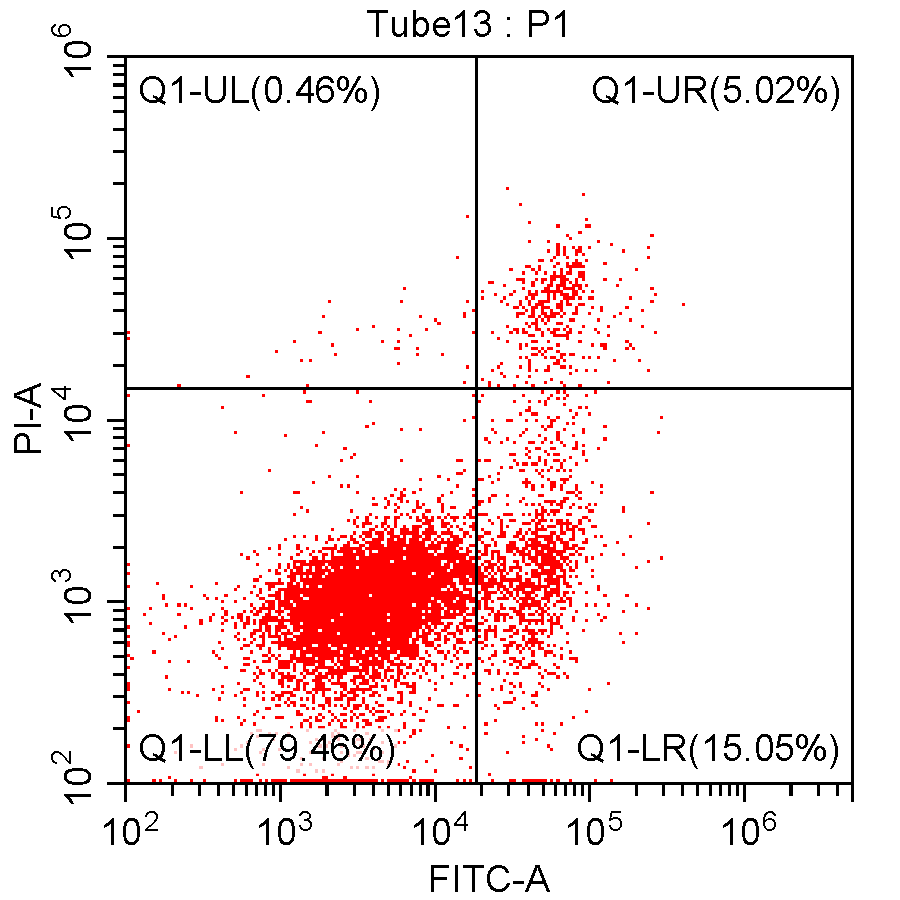

Supplement: Supplementary file 3 [file DataSheet2.ZIP › Flow cytometry/Fig.1C 6 groups/Tube14_Plot1.bmp]

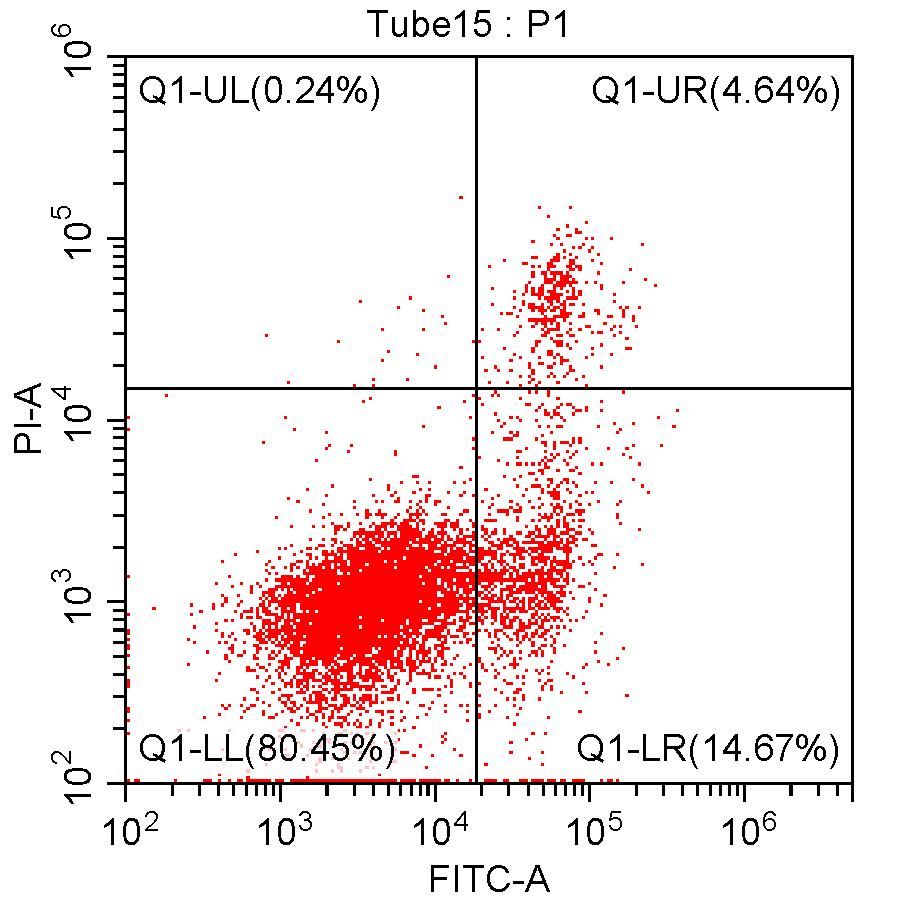

Supplement: Supplementary file 3 [file DataSheet2.ZIP › Flow cytometry/Fig.1C 6 groups/Tube15_Plot1.bmp]

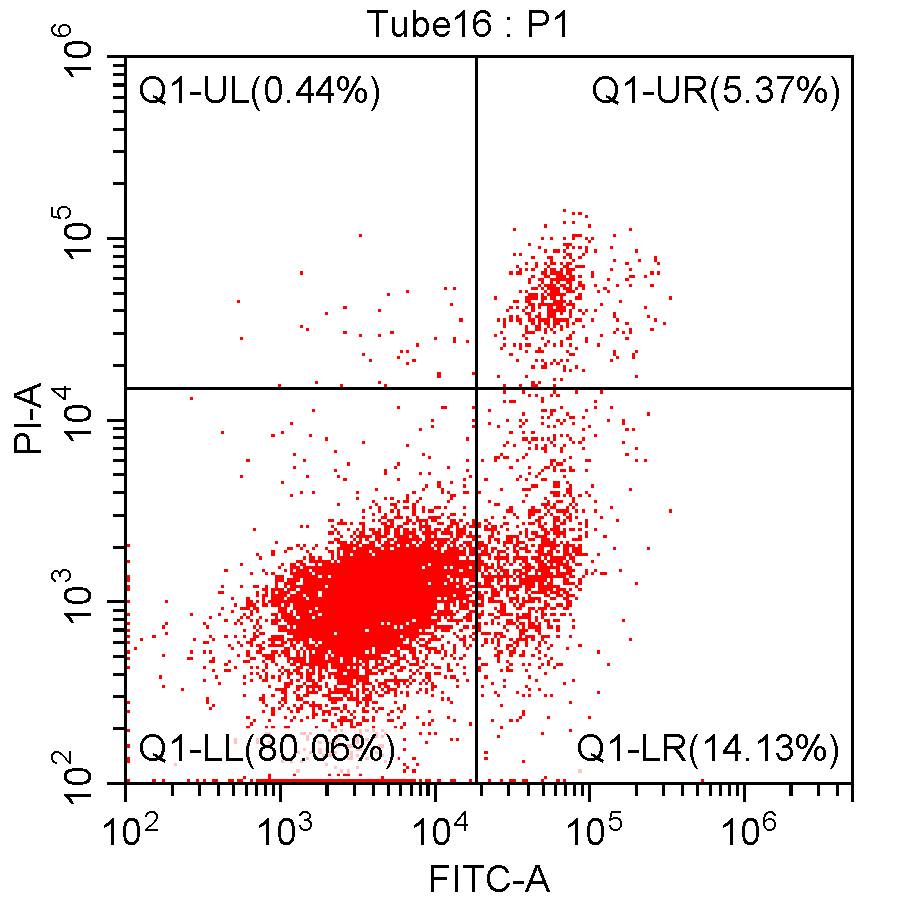

Supplement: Supplementary file 3 [file DataSheet2.ZIP › Flow cytometry/Fig.1C 6 groups/Tube16_Plot1.bmp]

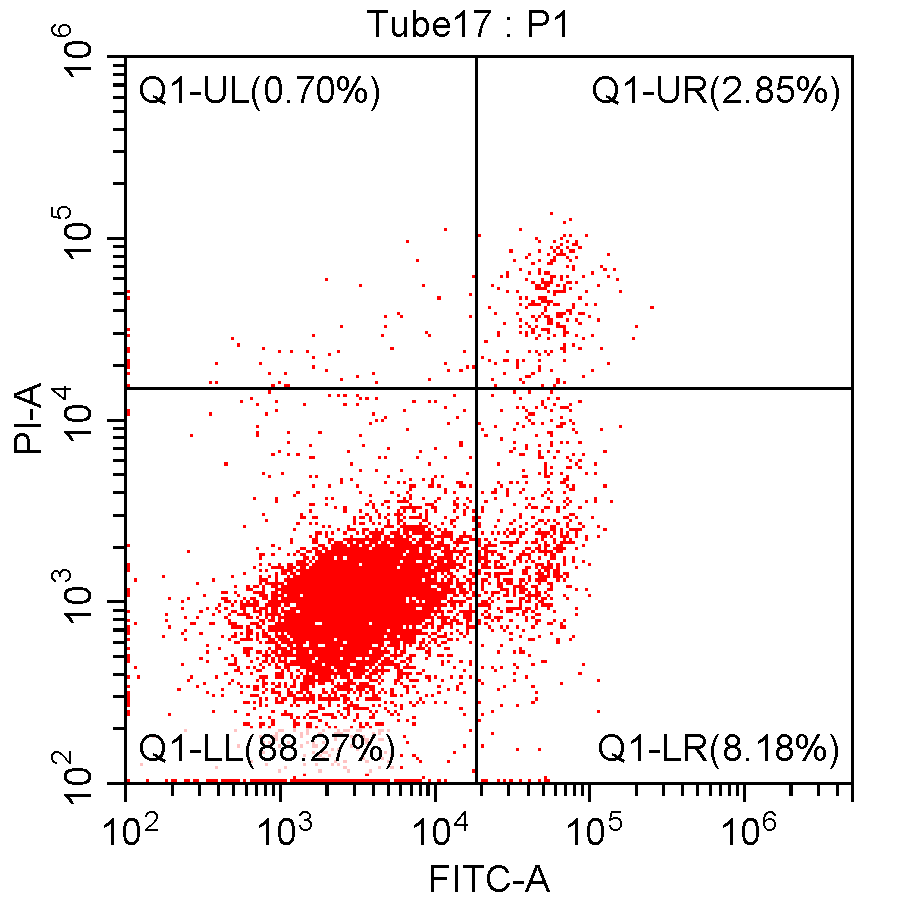

Supplement: Supplementary file 3 [file DataSheet2.ZIP › Flow cytometry/Fig.1C 6 groups/Tube17_Plot1.bmp]

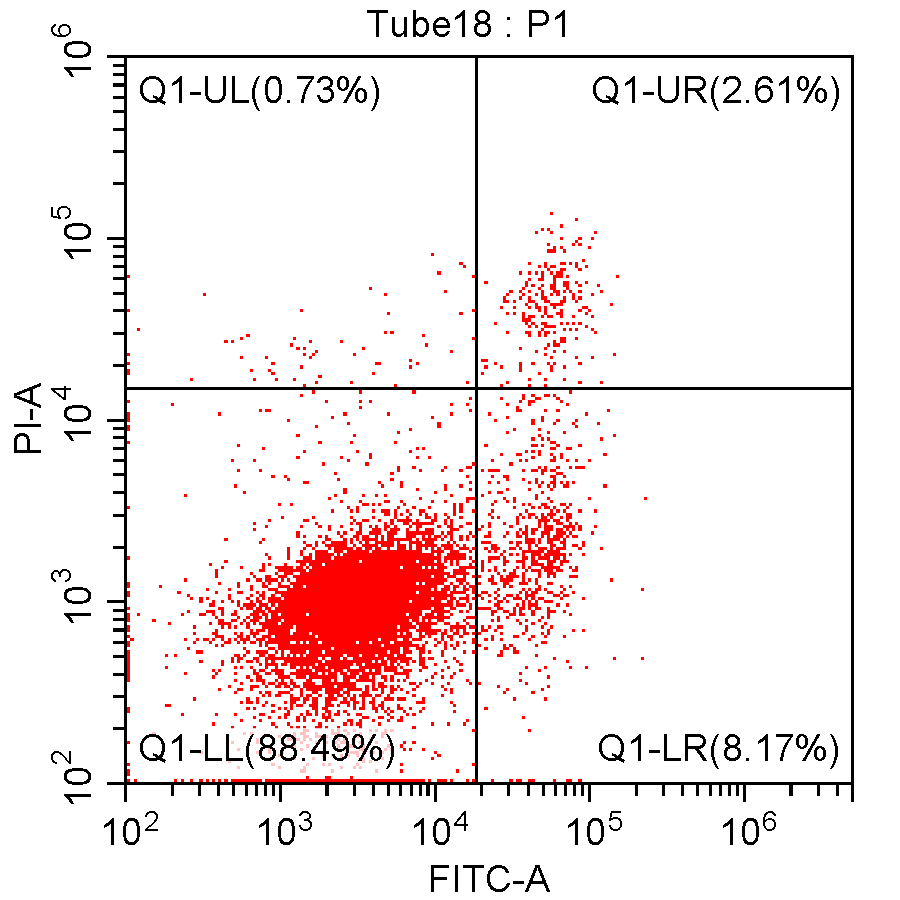

Supplement: Supplementary file 3 [file DataSheet2.ZIP › Flow cytometry/Fig.1C 6 groups/Tube18_Plot1.bmp]

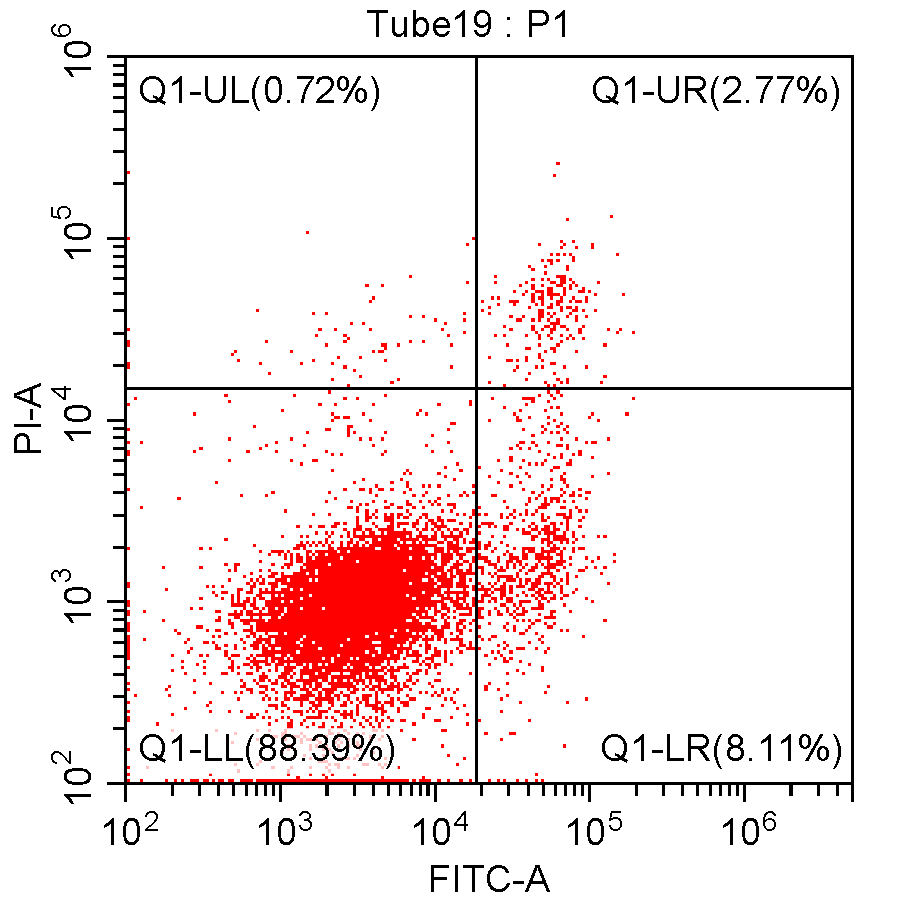

Supplement: Supplementary file 3 [file DataSheet2.ZIP › Flow cytometry/Fig.1C 6 groups/Tube19_Plot1.bmp]

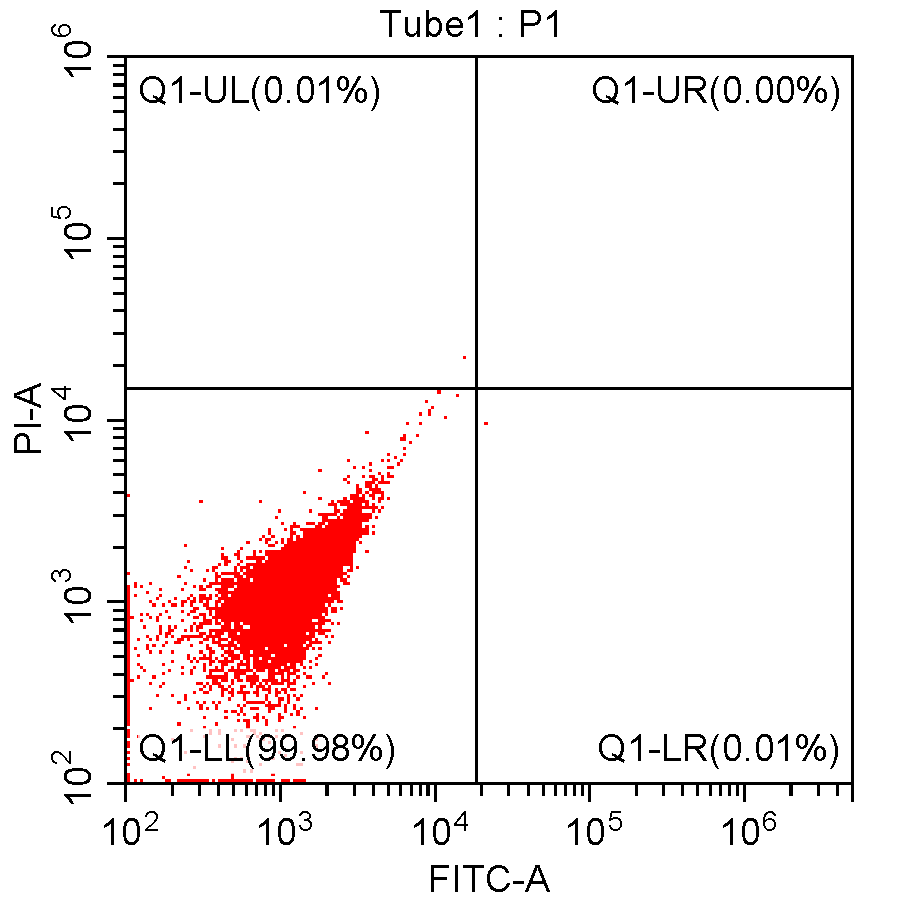

Supplement: Supplementary file 3 [file DataSheet2.ZIP › Flow cytometry/Fig.1C 6 groups/Tube1_Plot1.bmp]

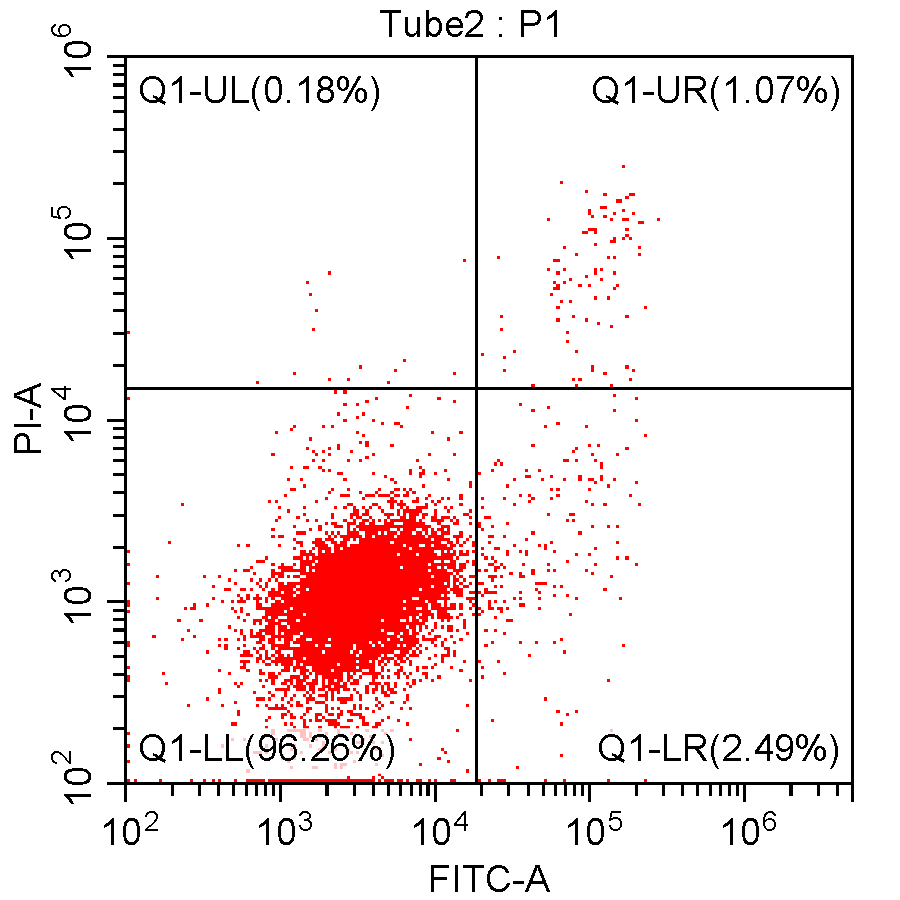

Supplement: Supplementary file 3 [file DataSheet2.ZIP › Flow cytometry/Fig.1C 6 groups/Tube2_Plot1.bmp]

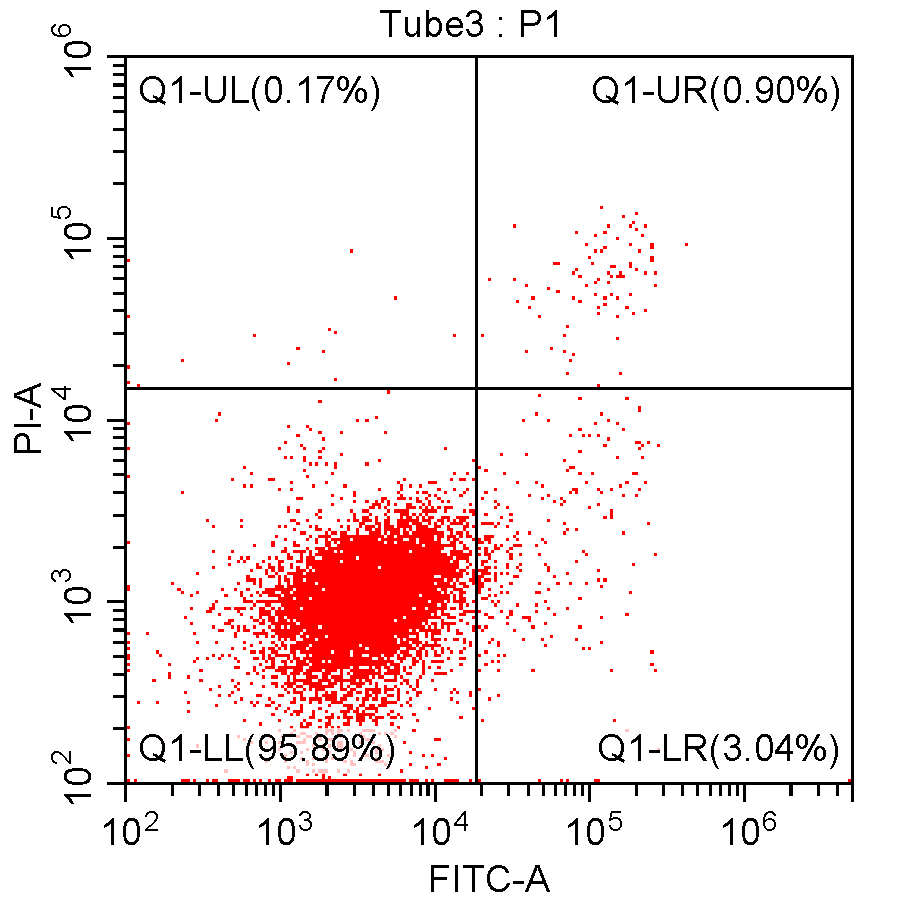

Supplement: Supplementary file 3 [file DataSheet2.ZIP › Flow cytometry/Fig.1C 6 groups/Tube3_Plot1.bmp]

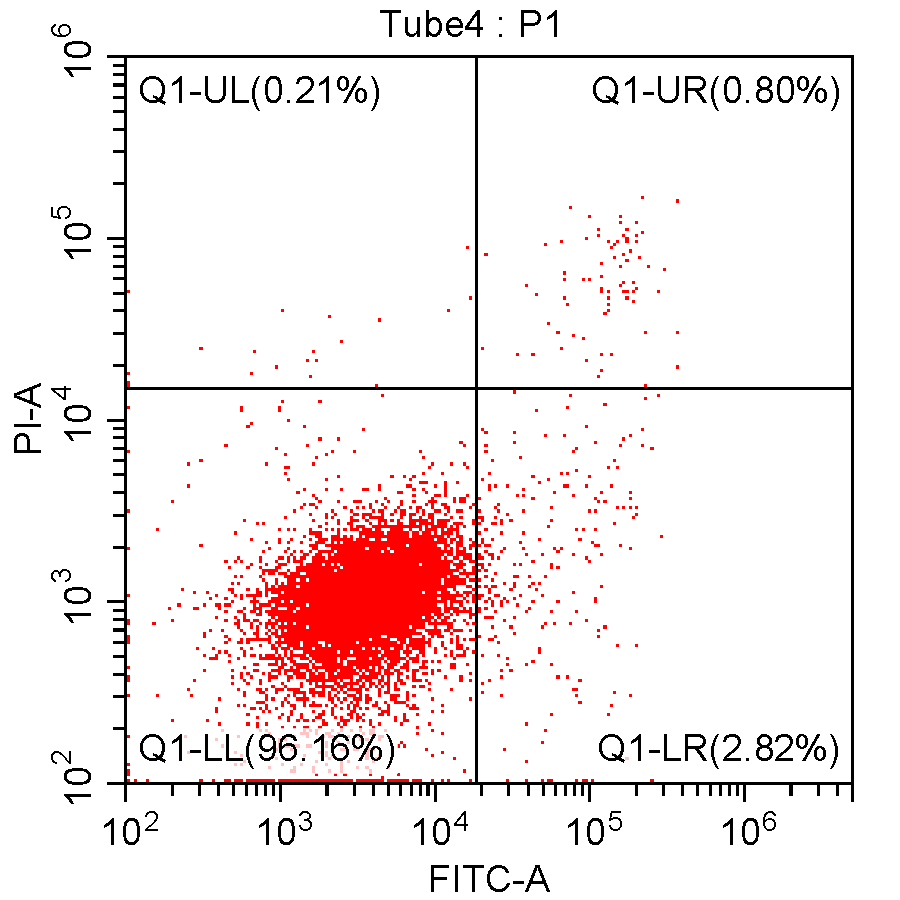

Supplement: Supplementary file 3 [file DataSheet2.ZIP › Flow cytometry/Fig.1C 6 groups/Tube4_Plot1.bmp]

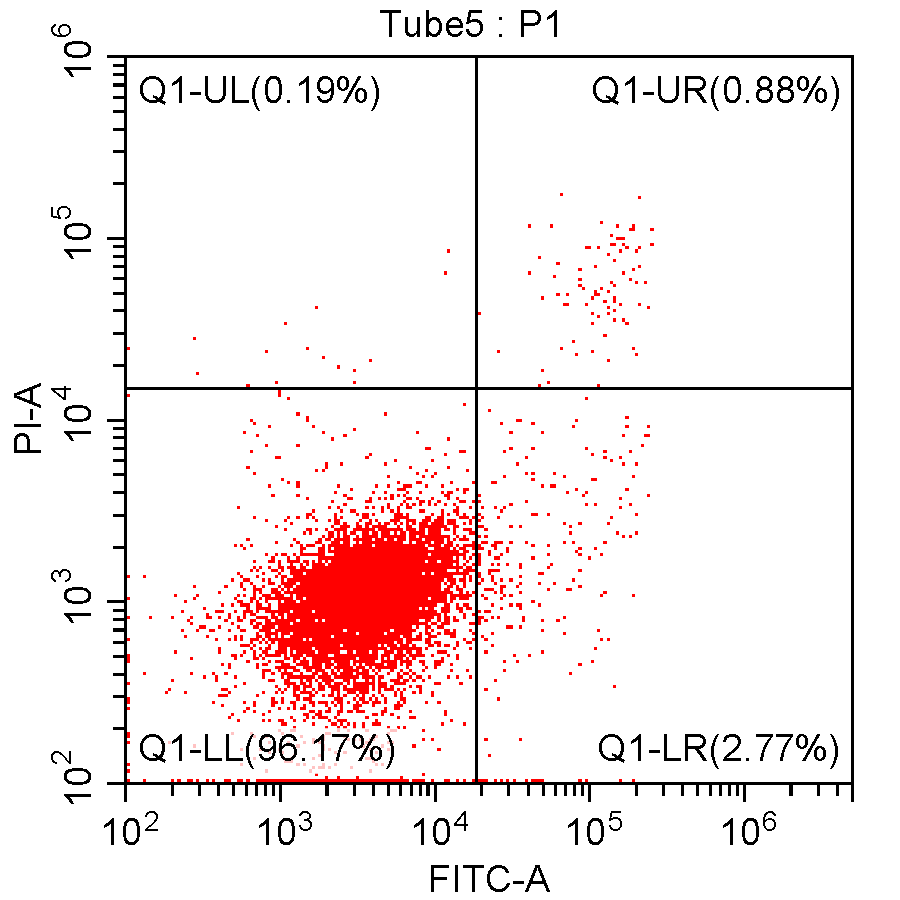

Supplement: Supplementary file 3 [file DataSheet2.ZIP › Flow cytometry/Fig.1C 6 groups/Tube5_Plot1.bmp]

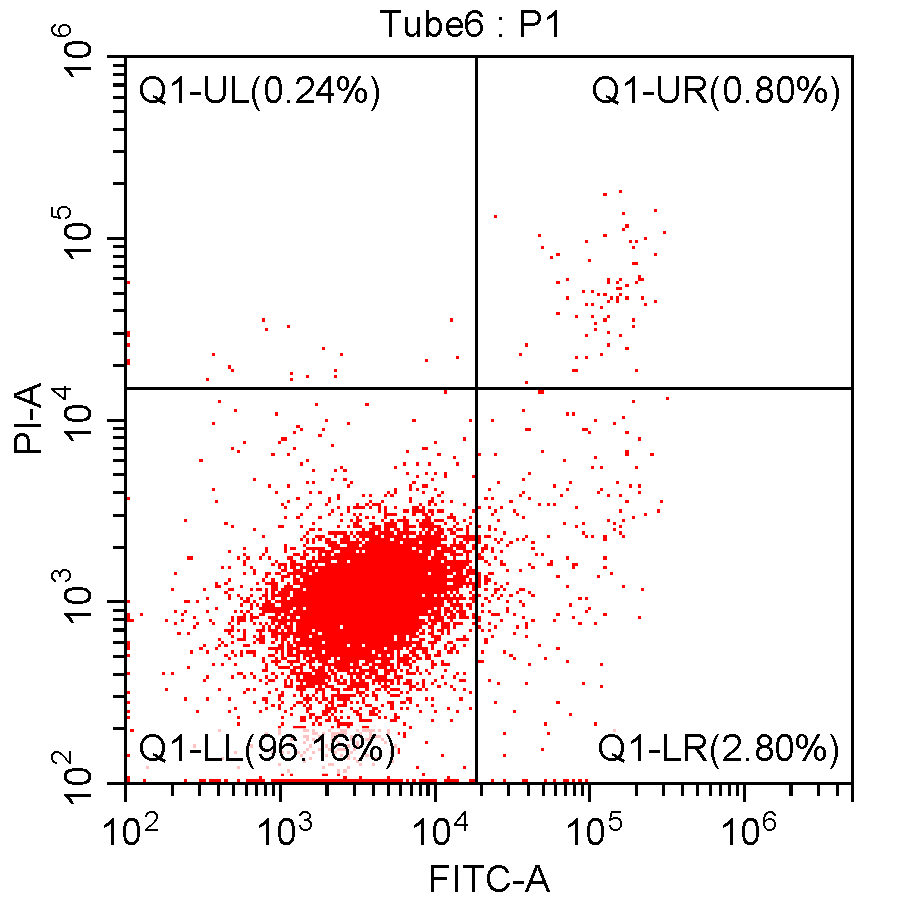

Supplement: Supplementary file 3 [file DataSheet2.ZIP › Flow cytometry/Fig.1C 6 groups/Tube6_Plot1.bmp]

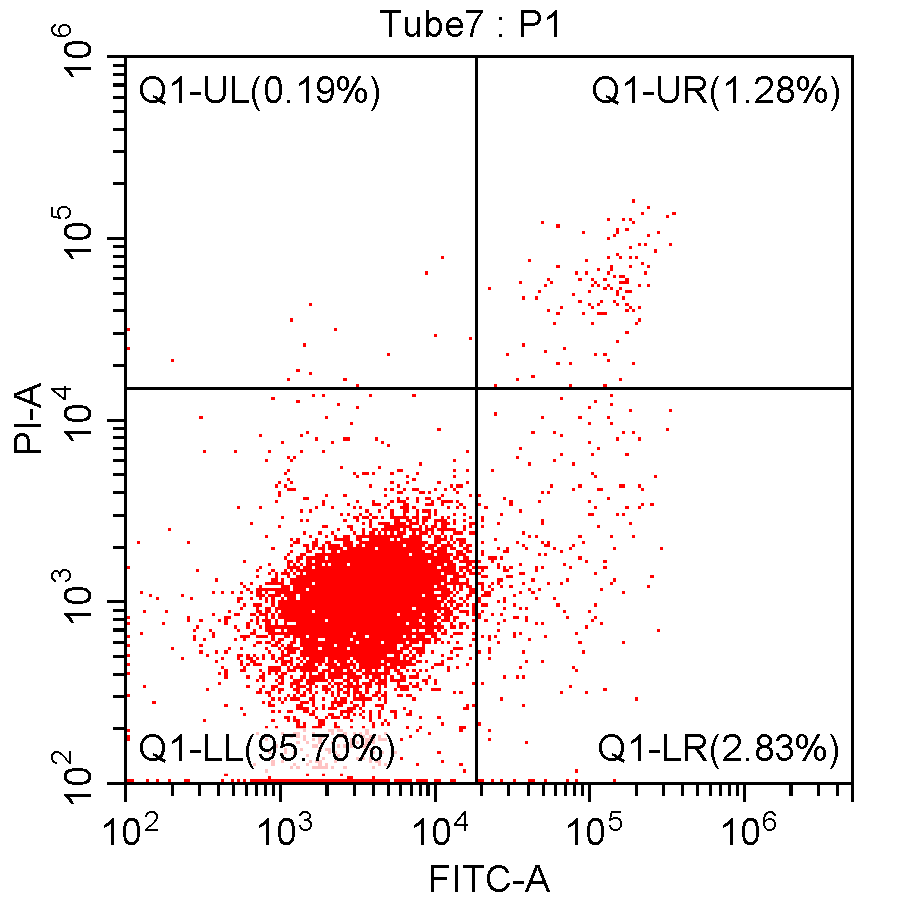

Supplement: Supplementary file 3 [file DataSheet2.ZIP › Flow cytometry/Fig.1C 6 groups/Tube7_Plot1.bmp]

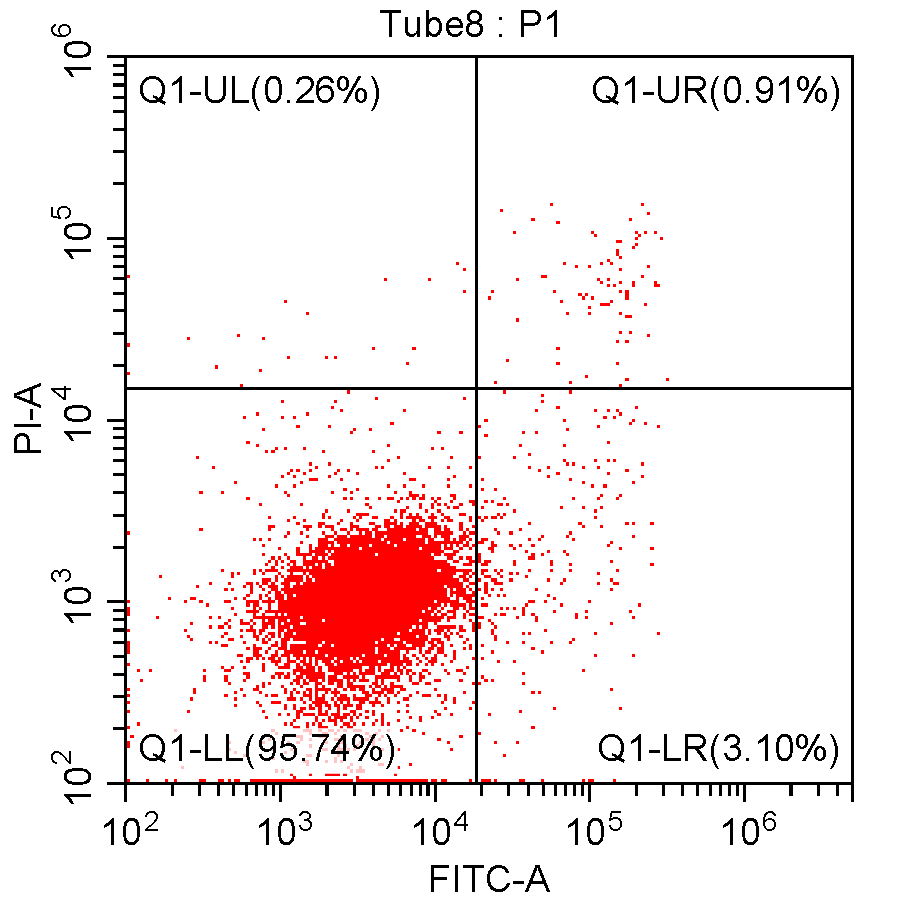

Supplement: Supplementary file 3 [file DataSheet2.ZIP › Flow cytometry/Fig.1C 6 groups/Tube8_Plot1.bmp]

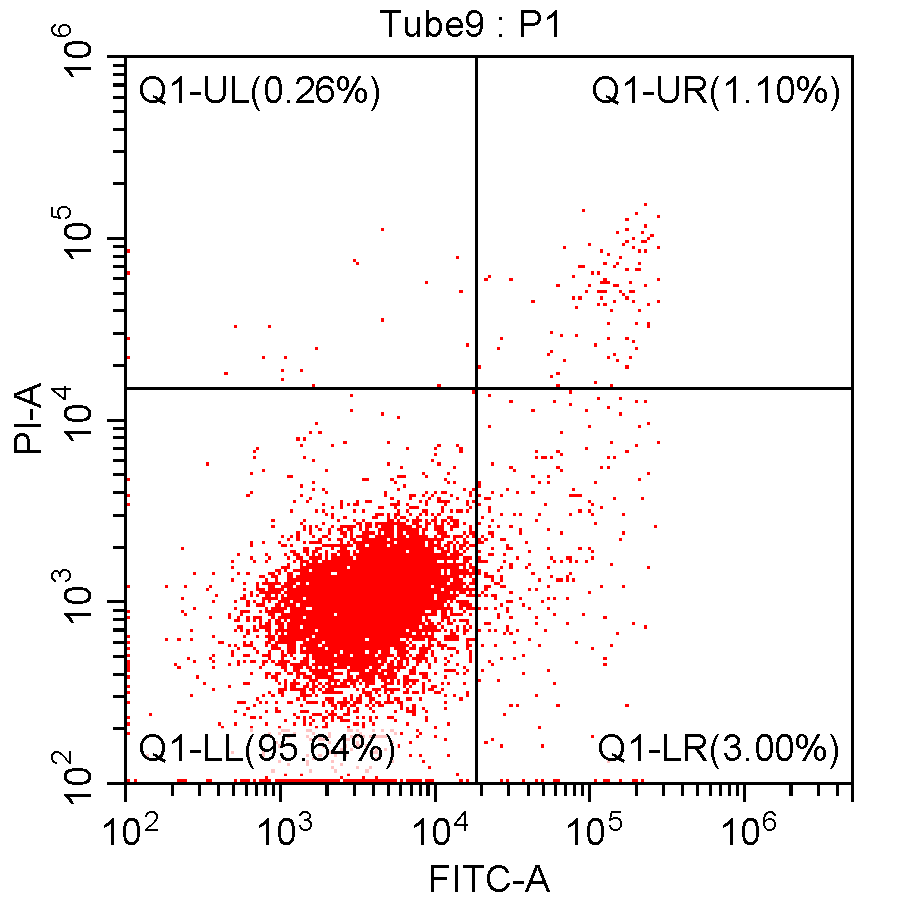

Supplement: Supplementary file 3 [file DataSheet2.ZIP › Flow cytometry/Fig.1C 6 groups/Tube9_Plot1.bmp]

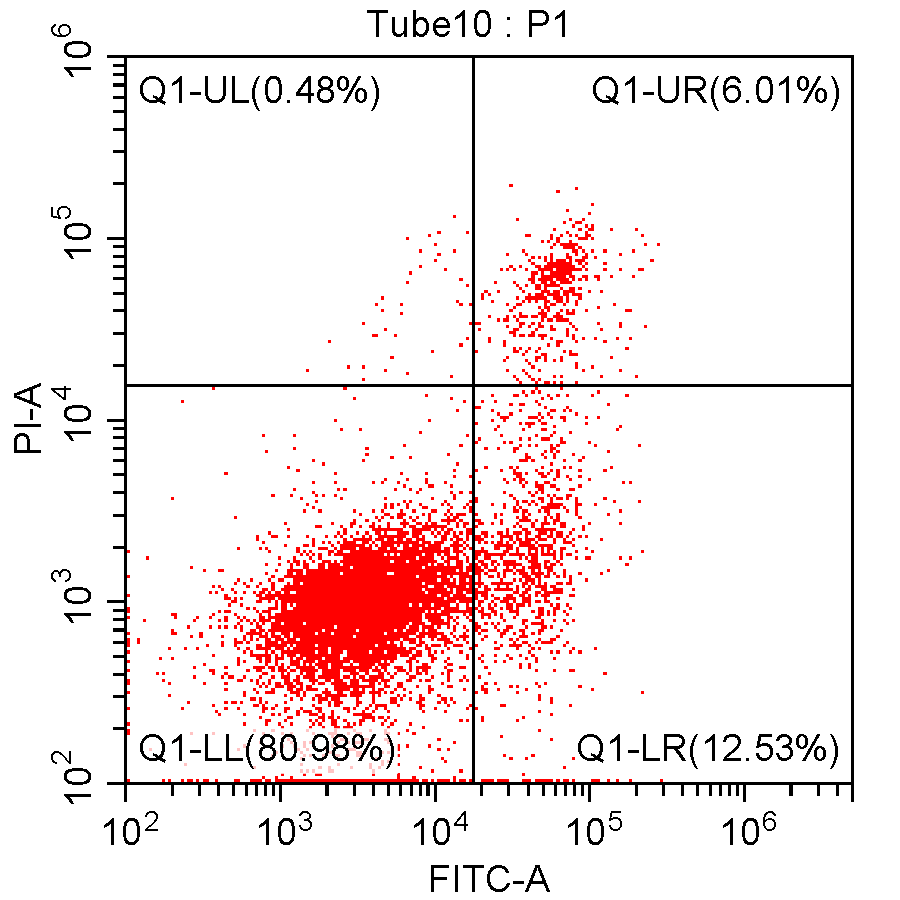

Supplement: Supplementary file 3 [file DataSheet2.ZIP › Flow cytometry/Fig.3D 7groups/Tube10_Plot1.bmp]

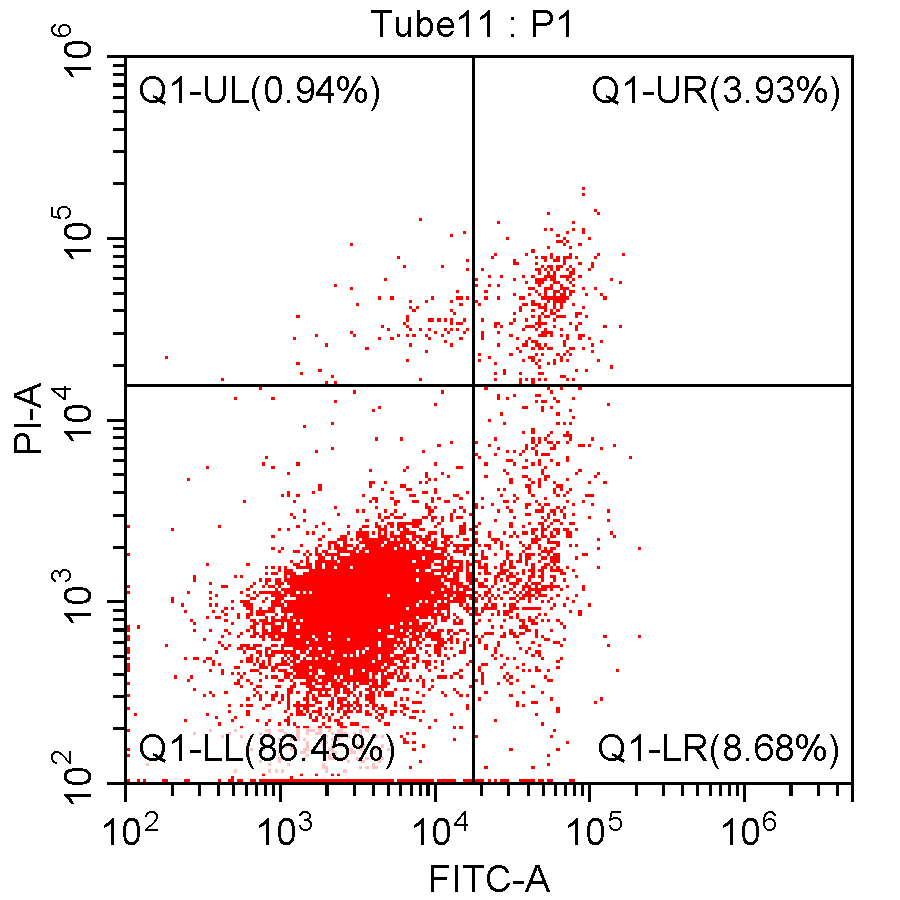

Supplement: Supplementary file 3 [file DataSheet2.ZIP › Flow cytometry/Fig.3D 7groups/Tube11_Plot1.bmp]

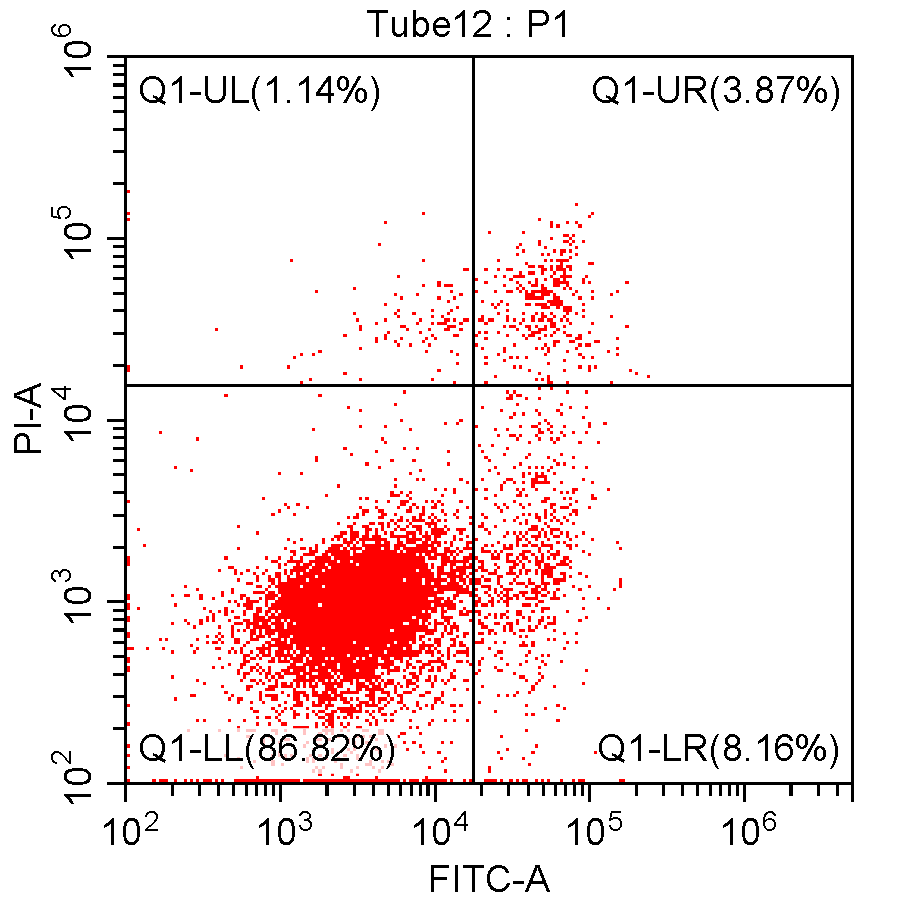

Supplement: Supplementary file 3 [file DataSheet2.ZIP › Flow cytometry/Fig.3D 7groups/Tube12_Plot1.bmp]

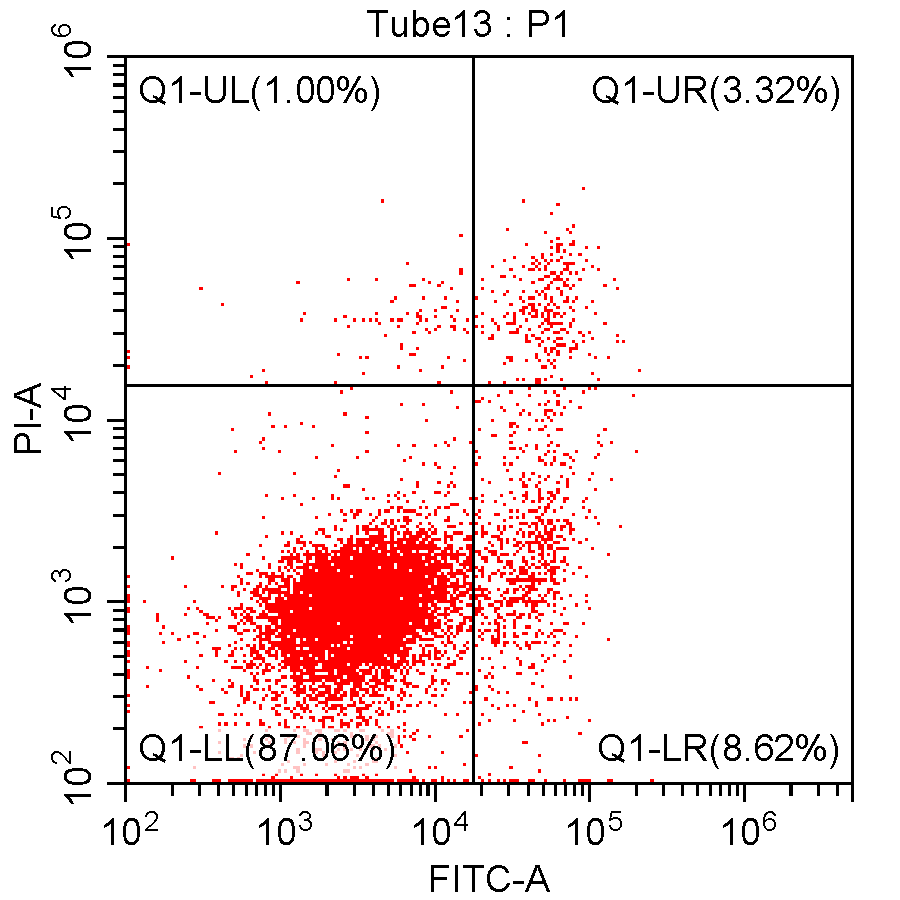

Supplement: Supplementary file 3 [file DataSheet2.ZIP › Flow cytometry/Fig.3D 7groups/Tube13_Plot1.bmp]

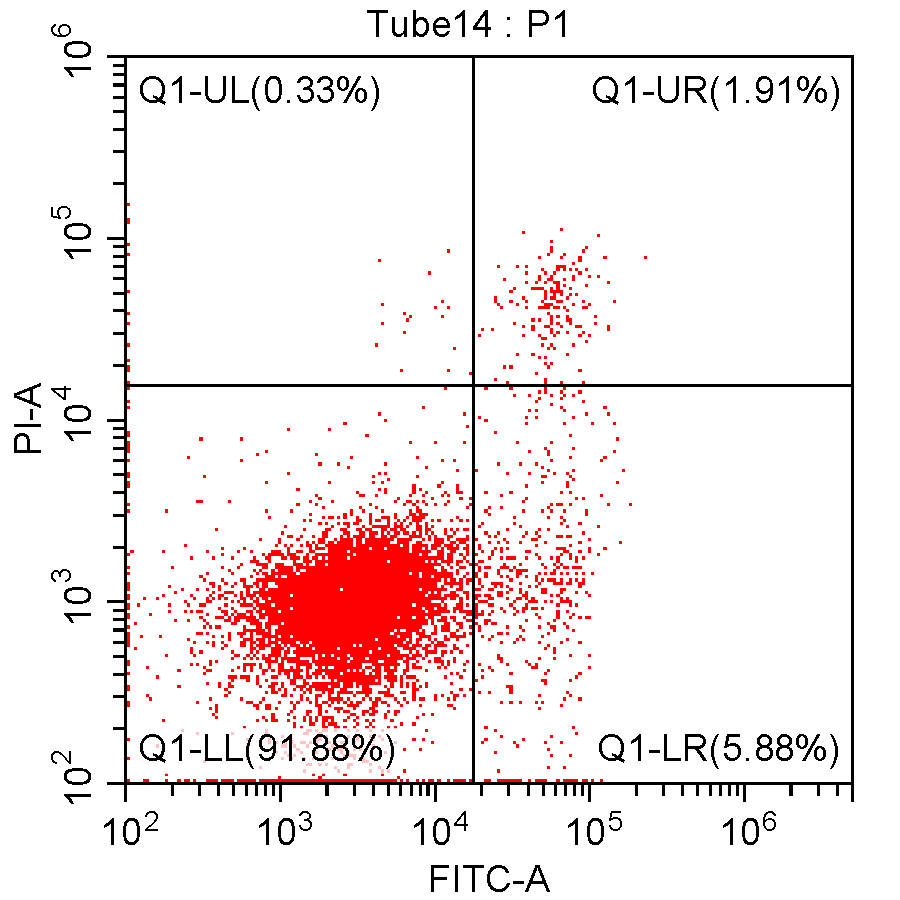

Supplement: Supplementary file 3 [file DataSheet2.ZIP › Flow cytometry/Fig.3D 7groups/Tube14_Plot1.bmp]

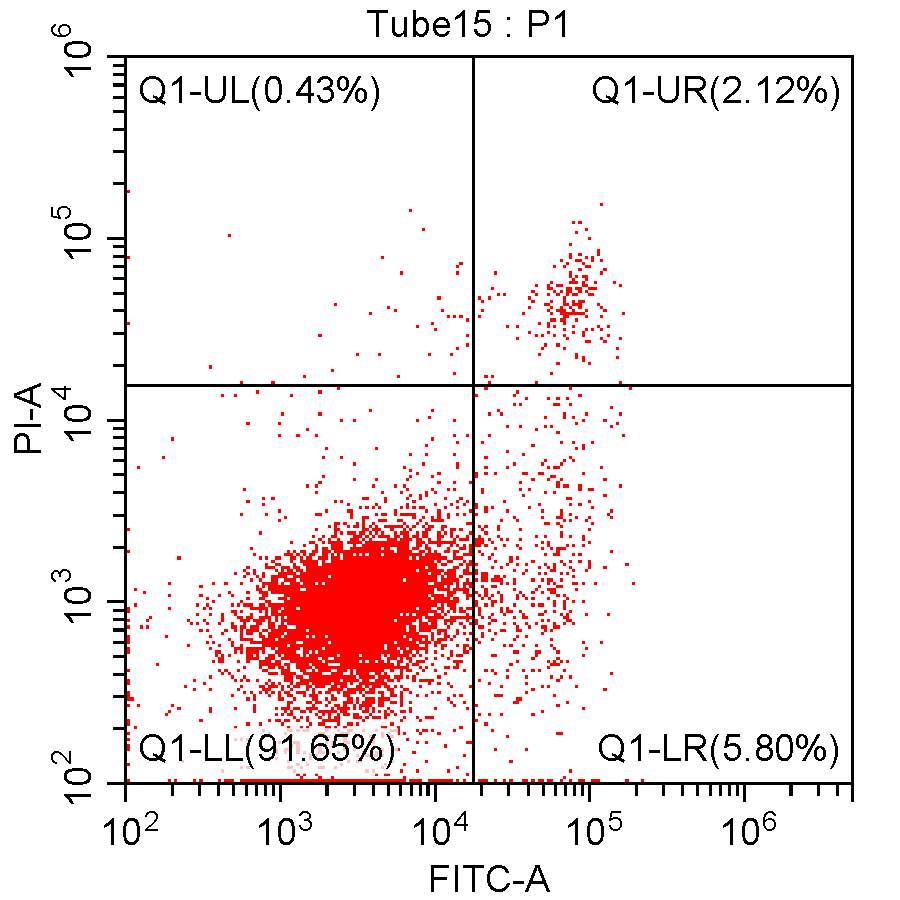

Supplement: Supplementary file 3 [file DataSheet2.ZIP › Flow cytometry/Fig.3D 7groups/Tube15_Plot1.bmp]
