# Supplementary figures and images for: Liuwei Dihuang Pills Inhibit Podocyte Injury and Alleviate IgA Nephropathy by Directly Altering Mesangial Cell-Derived Exosome Function and Secretion (part 2 of 2)
Source: Front Pharmacol. 2022 Jul 11;13:889008. doi: 10.3389/fphar.2022.889008 (PMC9309816; doi:10.3389/fphar.2022.889008)

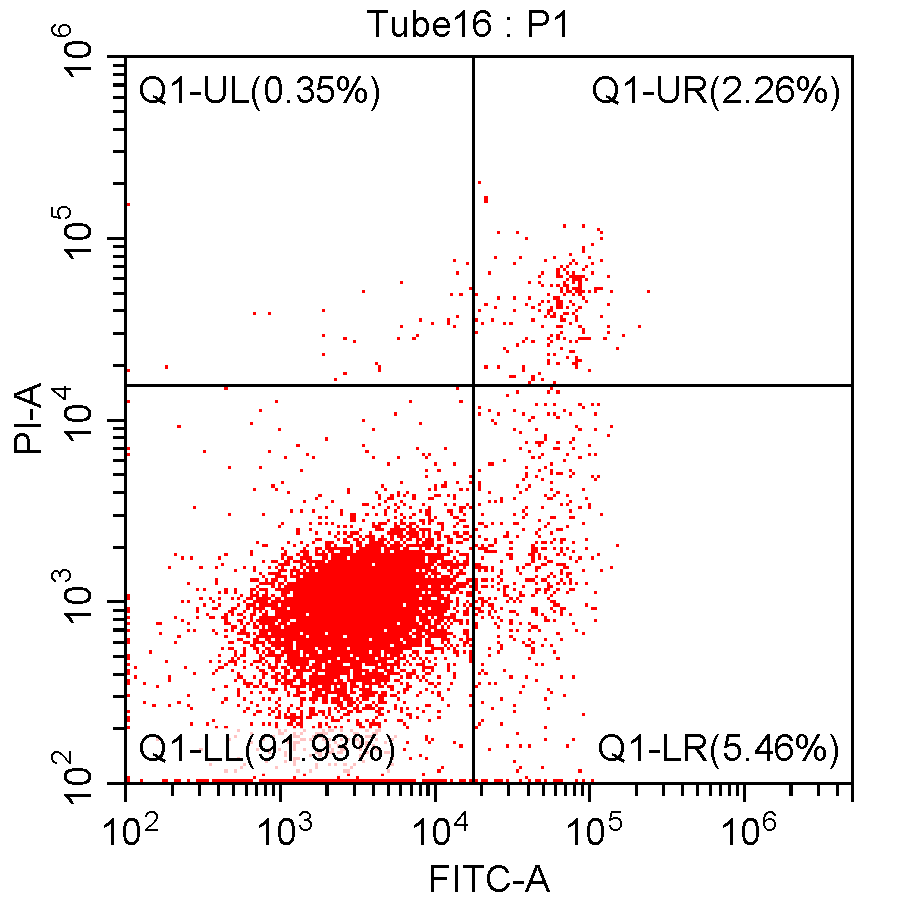

Supplement: Supplementary file 3 [file DataSheet2.ZIP › Flow cytometry/Fig.3D 7groups/Tube16_Plot1.bmp]

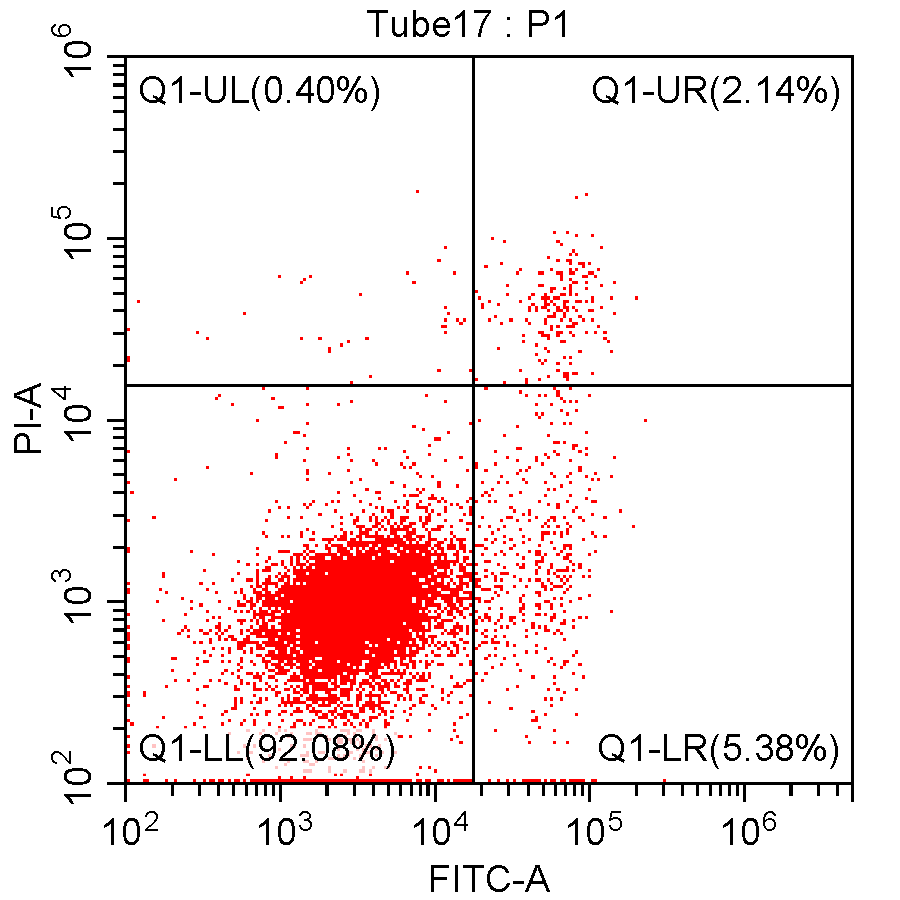

Supplement: Supplementary file 3 [file DataSheet2.ZIP › Flow cytometry/Fig.3D 7groups/Tube17_Plot1.bmp]

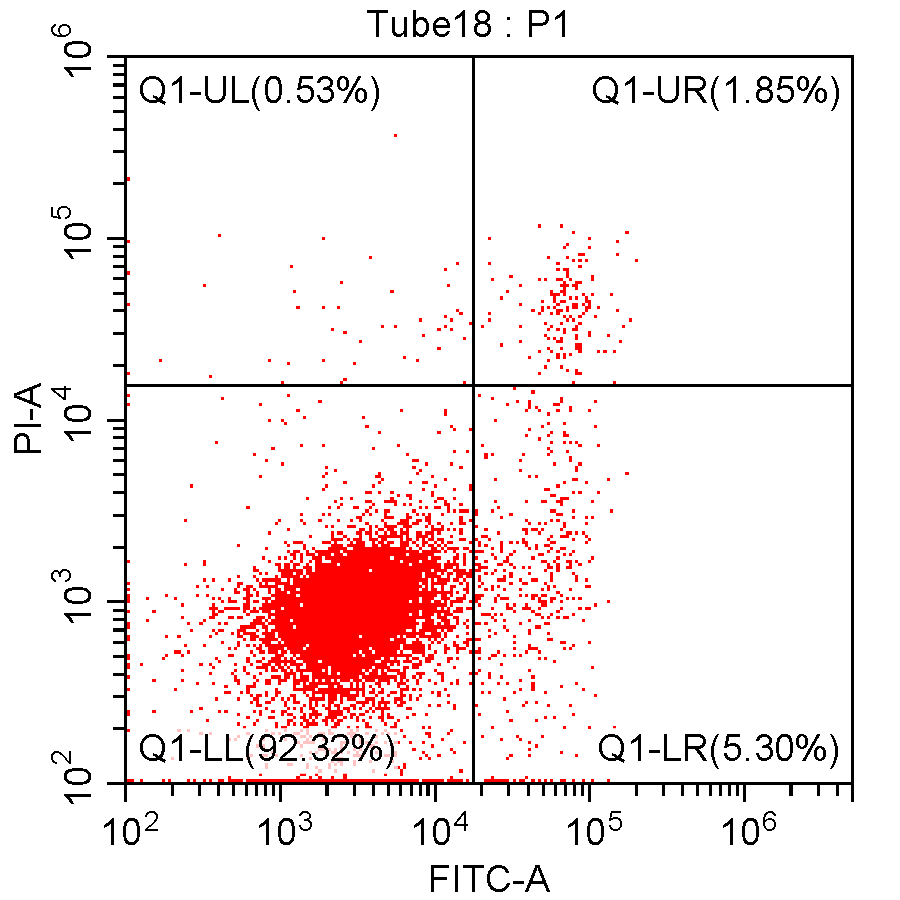

Supplement: Supplementary file 3 [file DataSheet2.ZIP › Flow cytometry/Fig.3D 7groups/Tube18_Plot1.bmp]

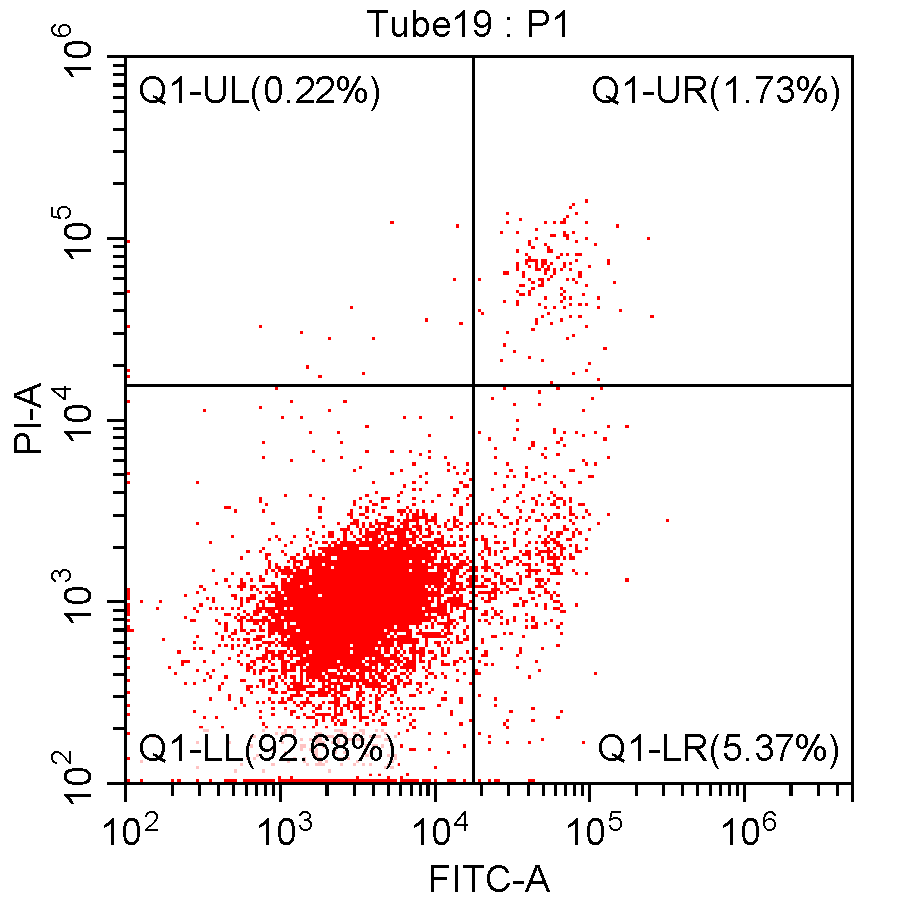

Supplement: Supplementary file 3 [file DataSheet2.ZIP › Flow cytometry/Fig.3D 7groups/Tube19_Plot1.bmp]

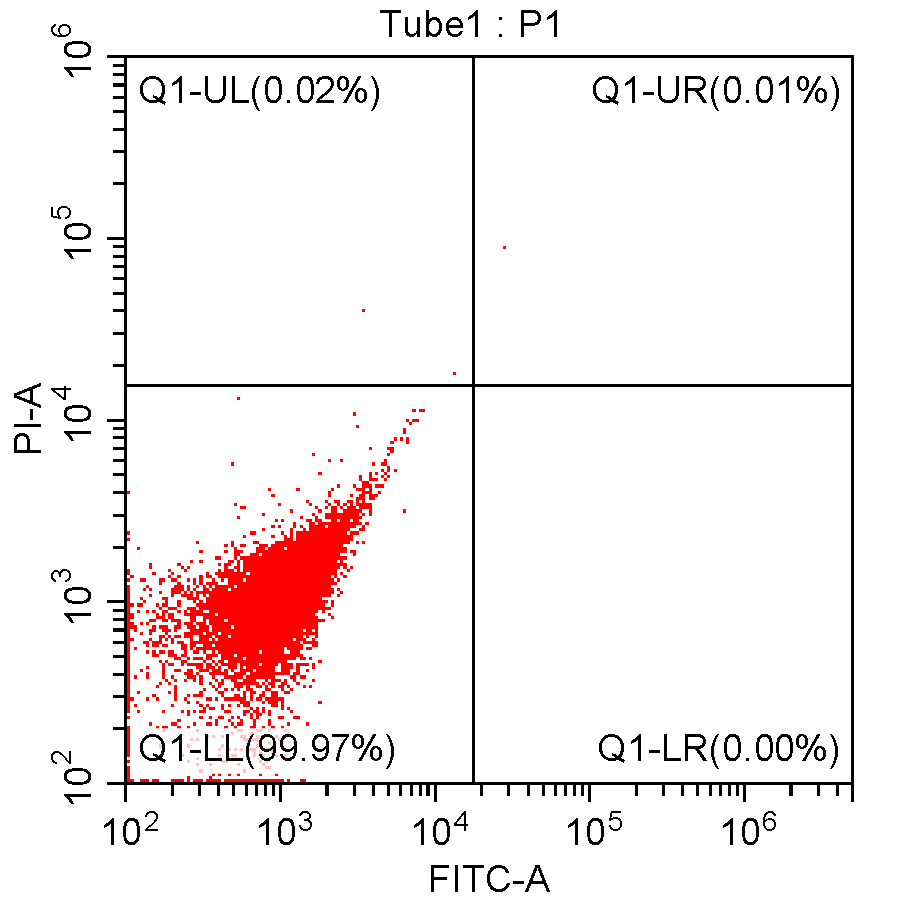

Supplement: Supplementary file 3 [file DataSheet2.ZIP › Flow cytometry/Fig.3D 7groups/Tube1_Plot1.bmp]

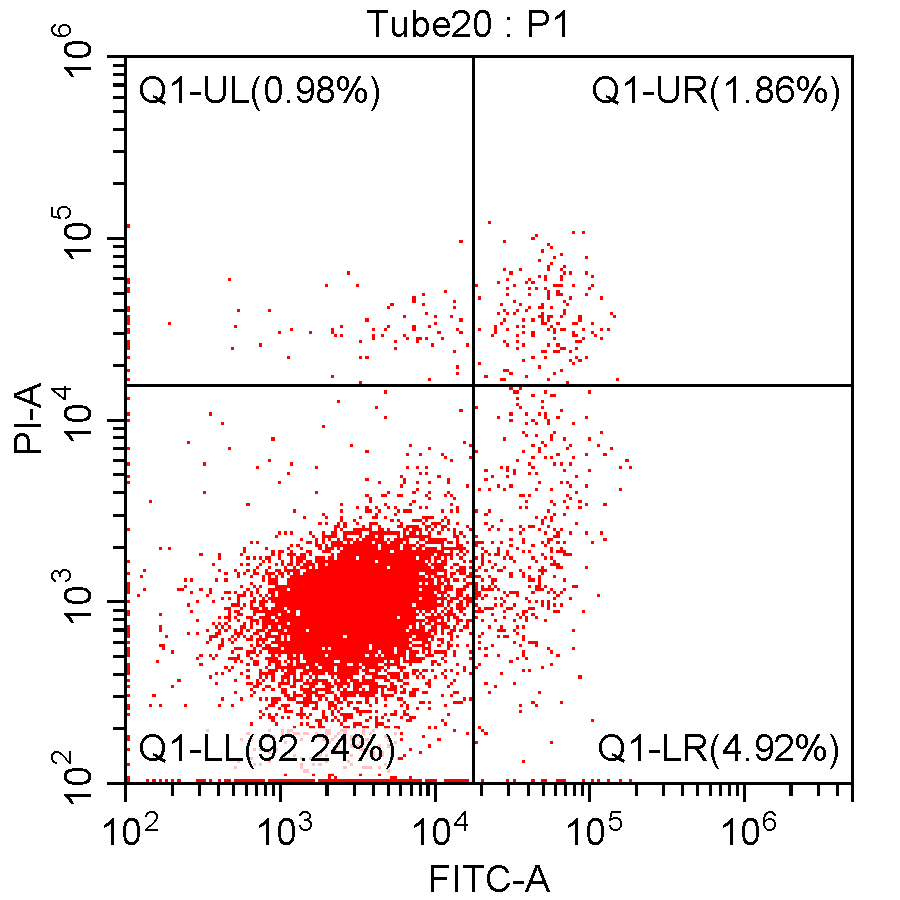

Supplement: Supplementary file 3 [file DataSheet2.ZIP › Flow cytometry/Fig.3D 7groups/Tube20_Plot1.bmp]

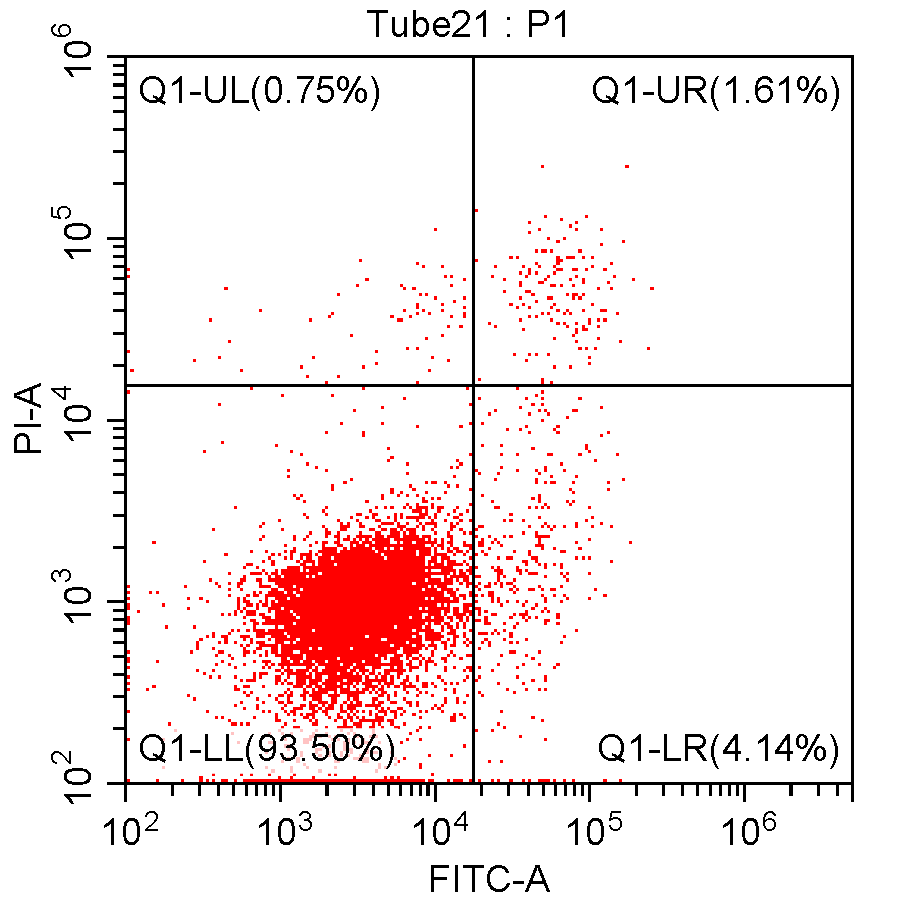

Supplement: Supplementary file 3 [file DataSheet2.ZIP › Flow cytometry/Fig.3D 7groups/Tube21_Plot1.bmp]

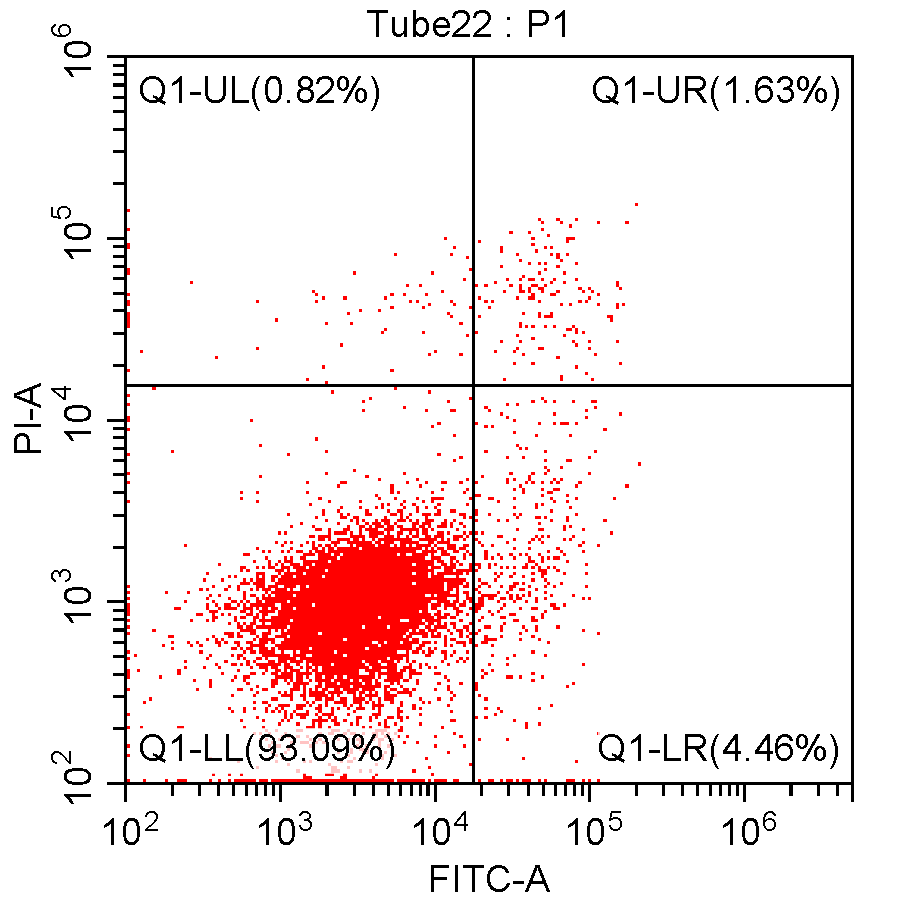

Supplement: Supplementary file 3 [file DataSheet2.ZIP › Flow cytometry/Fig.3D 7groups/Tube22_Plot1.bmp]

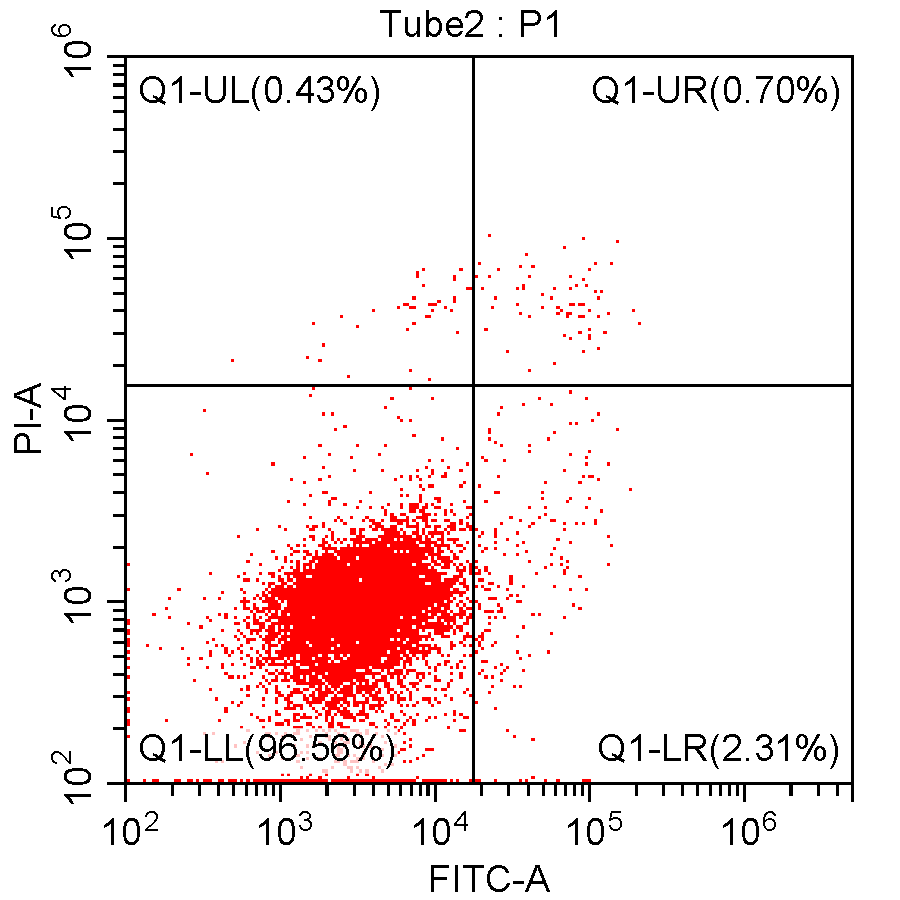

Supplement: Supplementary file 3 [file DataSheet2.ZIP › Flow cytometry/Fig.3D 7groups/Tube2_Plot1.bmp]

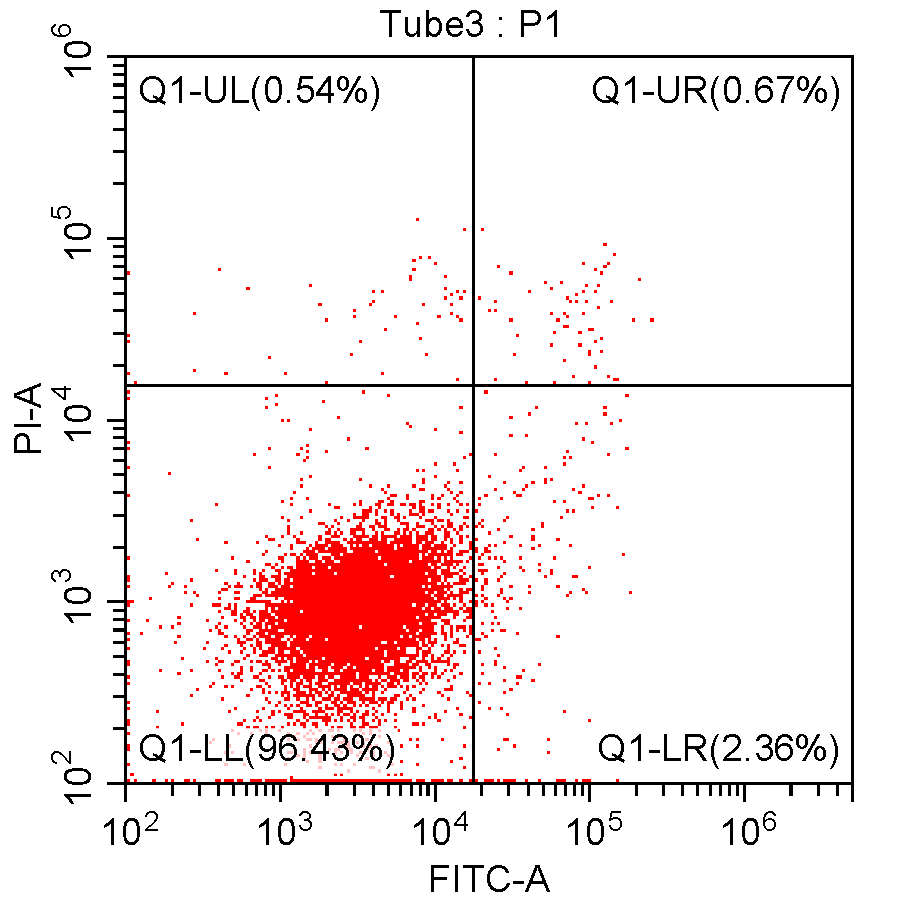

Supplement: Supplementary file 3 [file DataSheet2.ZIP › Flow cytometry/Fig.3D 7groups/Tube3_Plot1.bmp]

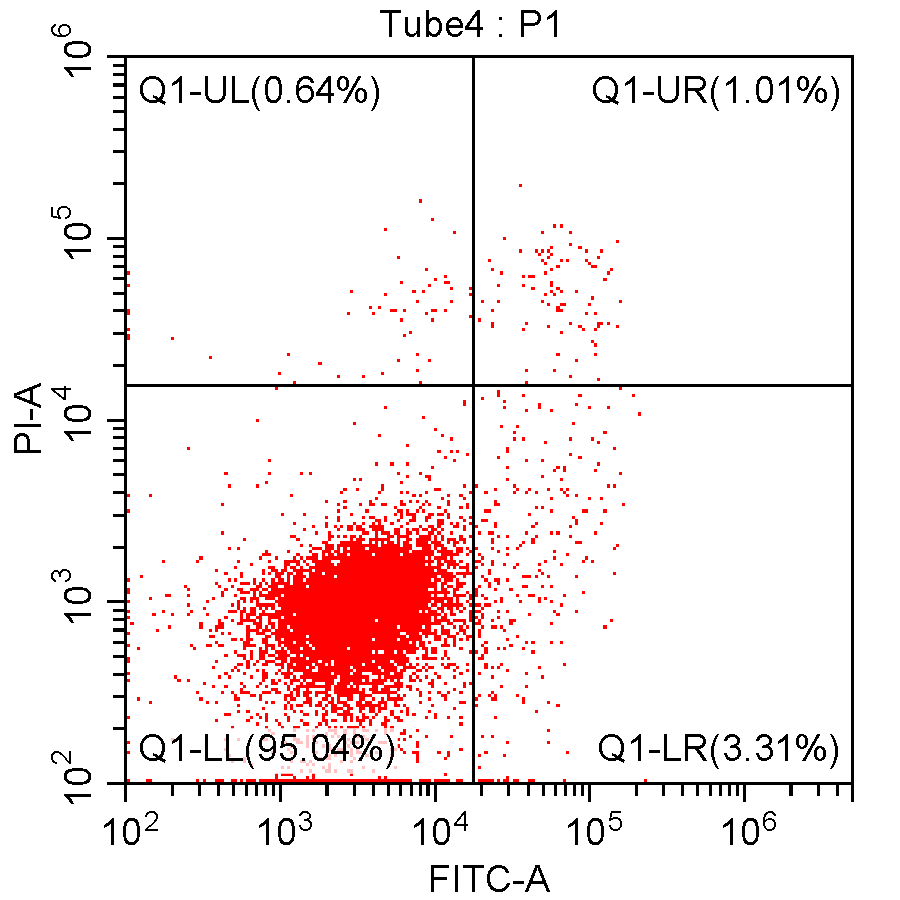

Supplement: Supplementary file 3 [file DataSheet2.ZIP › Flow cytometry/Fig.3D 7groups/Tube4_Plot1.bmp]

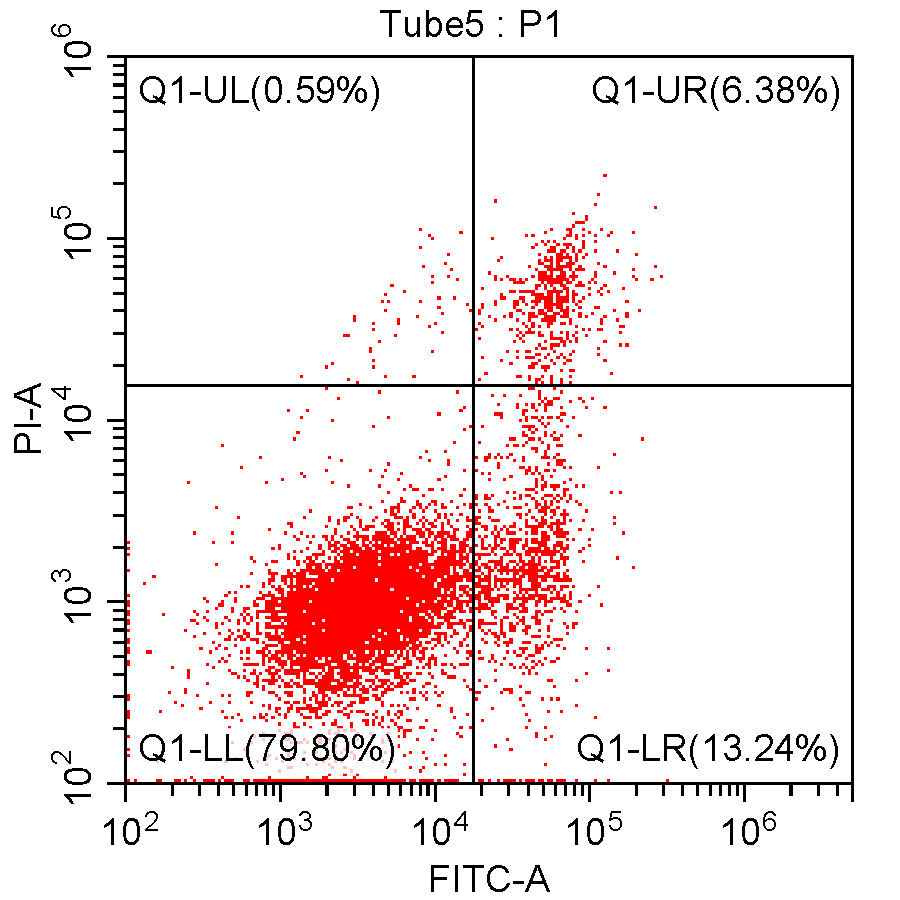

Supplement: Supplementary file 3 [file DataSheet2.ZIP › Flow cytometry/Fig.3D 7groups/Tube5_Plot1.bmp]

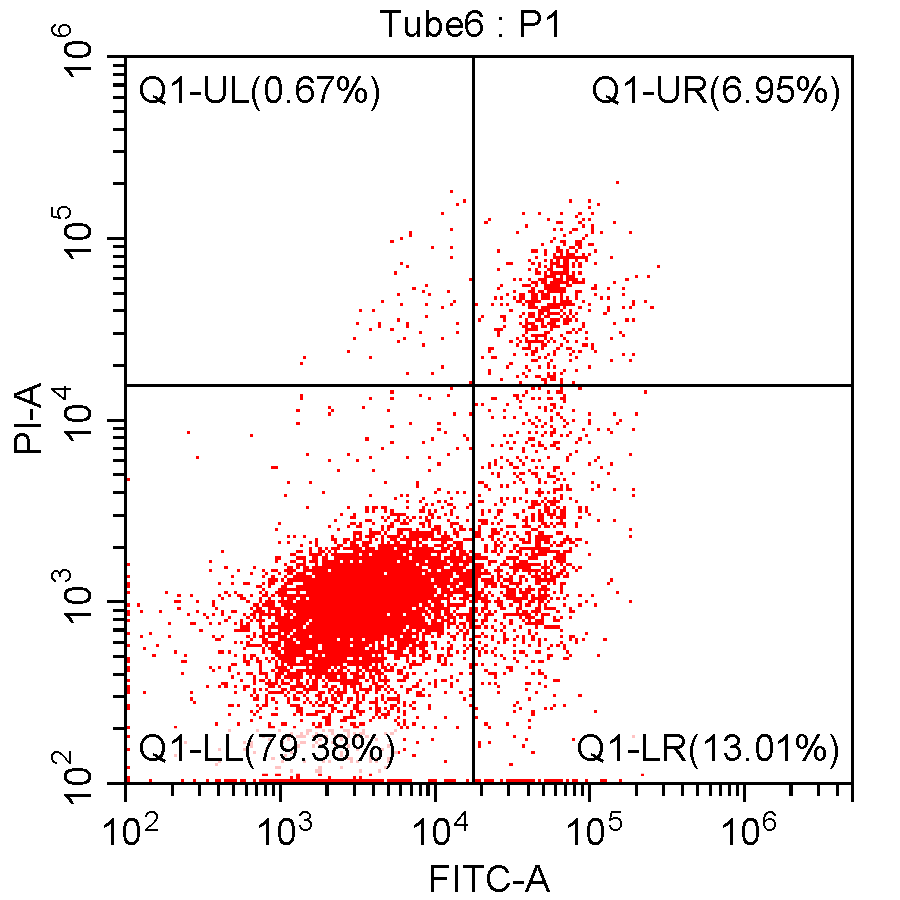

Supplement: Supplementary file 3 [file DataSheet2.ZIP › Flow cytometry/Fig.3D 7groups/Tube6_Plot1.bmp]

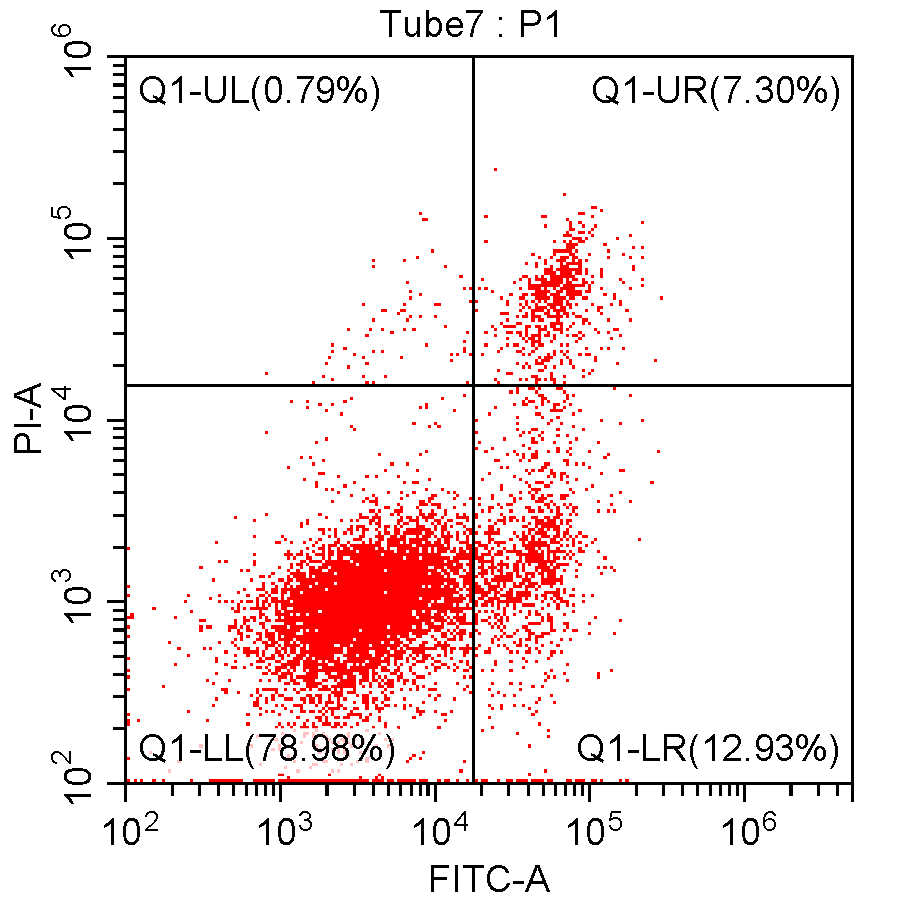

Supplement: Supplementary file 3 [file DataSheet2.ZIP › Flow cytometry/Fig.3D 7groups/Tube7_Plot1.bmp]

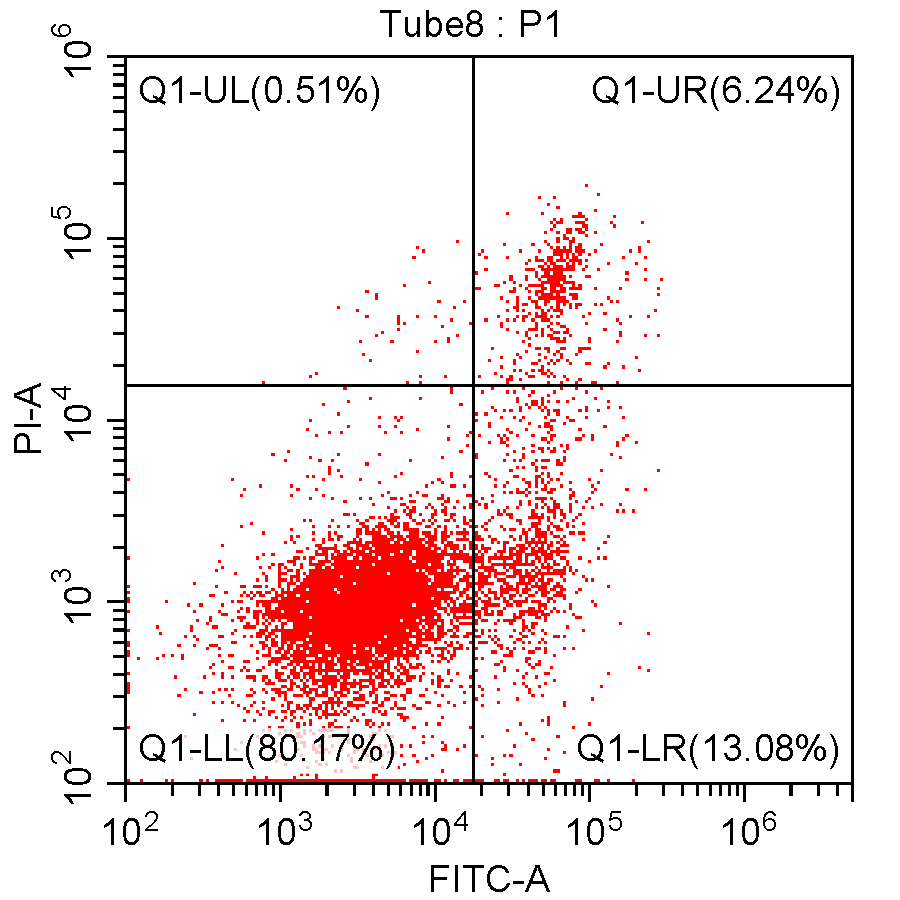

Supplement: Supplementary file 3 [file DataSheet2.ZIP › Flow cytometry/Fig.3D 7groups/Tube8_Plot1.bmp]

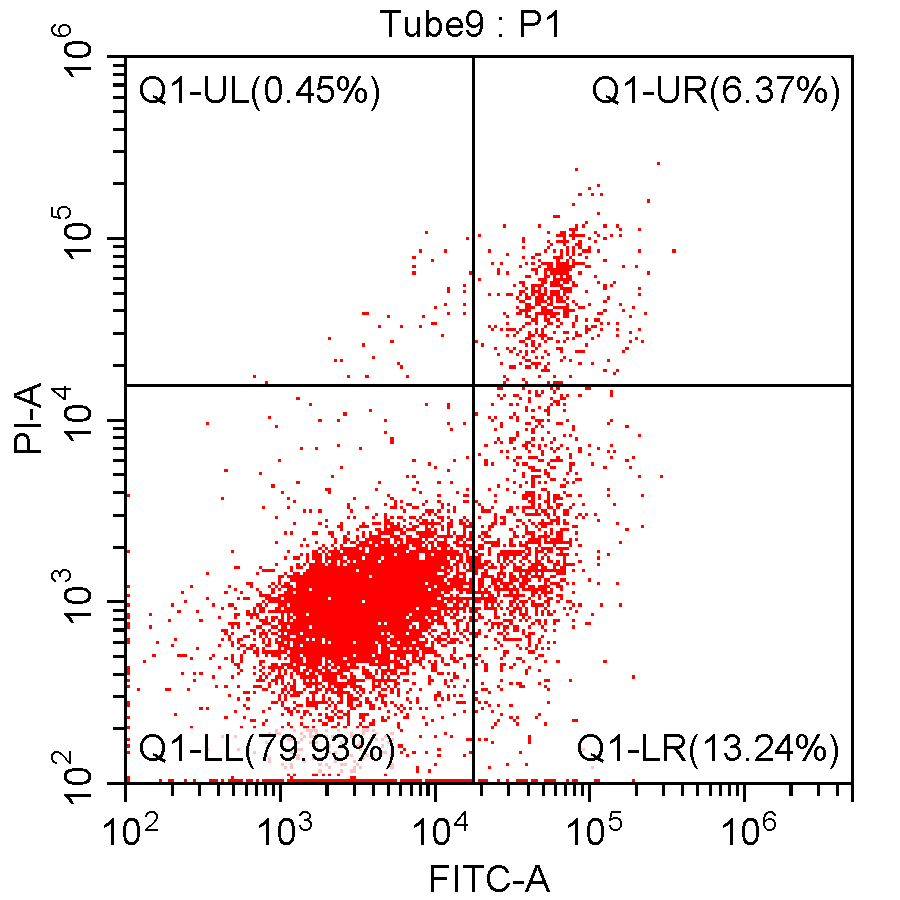

Supplement: Supplementary file 3 [file DataSheet2.ZIP › Flow cytometry/Fig.3D 7groups/Tube9_Plot1.bmp]
