# Supplementary material for: Liuwei Dihuang Pills Inhibit Podocyte Injury and Alleviate IgA Nephropathy by Directly Altering Mesangial Cell-Derived Exosome Function and Secretion
Source: Front Pharmacol. 2022 Jul 11;13:889008. doi: 10.3389/fphar.2022.889008 (PMC9309816; doi:10.3389/fphar.2022.889008)
Supplement: Supplementary file 4 [file Image1.pdf]

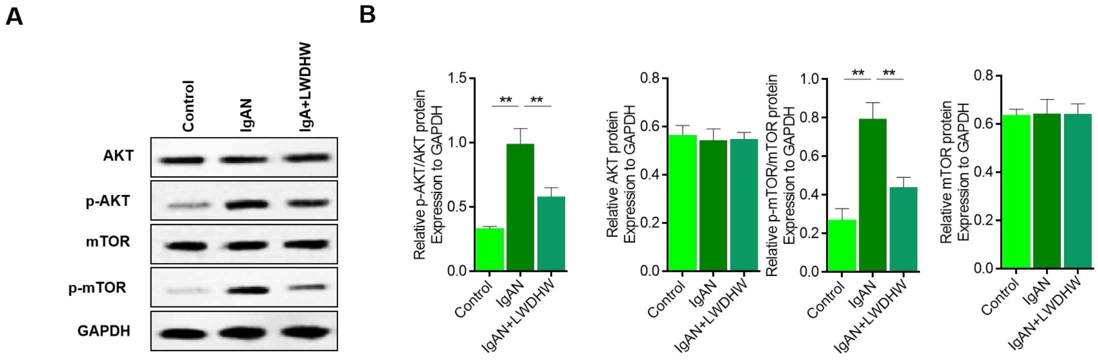

FigureS1. The effect of Liuwei Dihuang Pill on PI3K-Akt-MTOR pathway in IgAN mouse kidney was detected. (A) the expression content and phosphorylation level of Akt and mTOR in kidney tissue of mice in each group were detected by Western Blot method; (B) figure A quantification map. LWDHW means Liuwei Dihuang pills; * * means $p < 0.01$; * * means $p < 0.05$. * * it means $p < 0.01$, and ns means $p > 0.05$.
